# Chemical Bonding in Silicon Carbonyl Complexes

**DOI:** 10.1002/chem.202100493

**Published:** 2021-05-01

**Authors:** Tetiana Sergeieva, Debdeep Mandal, Diego M. Andrada

**Affiliations:** ^1^ Inorganic and Computational Chemistry Group Chemistry Department Saarland University Campus C4.1 66123 Saarbrücken Germany

**Keywords:** carbonyl complexes, chemical bonding, donor-acceptor, energy decomposition analysis, silylenes

## Abstract

Although silylene‐carbonyl complexes are known for decades, only recently isolable examples have been accomplished. In this work, the bonding situation is re‐evaluated to explain the origins of their remarkable stability within the Kohn‐Sham molecular orbital theory framework. It is shown that the chemical bond can be understood as CO interaction with the silylene via a donor‐acceptor interaction: a *σ*‐donation from the *σ*_CO_ into the empty *p‐*orbital of silicon, and a *π*‐back donation from the *sp* ^*2*^ lone pair of silicon into the π*_CO_ antibonding orbitals. Notably, it was established that the driving force behind the surprisingly stable Si−CO compounds, however, is another π‐back donation from a perpendicular bonding R−Si *σ*‐orbital into the π*_CO_ antibonding orbitals. Consequently, the pyramidalization of the central silicon atom cannot be associated with the strength of the *π*‐back donation, in sharp contrast to the established chemical bonding model. Considering this additional bonding interaction not only shed light on the bonding situation, but is also an indispensable key for broadening the scope of silylene‐carbonyl chemistry.

## Graphical abstract

**Revisiting the chemical bond** on silylene‐carbonyl species reveals a donor‐acceptor interaction between the CO and the silylene. The bonding can be conventionally understood as a σ‐donation from the σ_CO_ into the empty p‐orbital of silicon, and a π‐back donation from the sp^2^ lone pair of silicon into the π*_CO_ antibonding orbitals. Nonetheless, an unrecognized π‐back‐donation from the R−Si σ‐orbital into the π*_CO_ antibonding orbital faithfully explains the relationship between structure and stability.

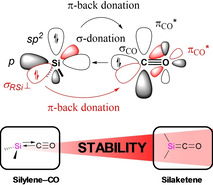

## Introduction

The chemistry of transition metal‐carbonyl complexes has been the focus of attention for more than a century.^[1]^ These complexes evolved from laboratory curiosities into essential precursors for a wide range of chemical transformations.^[2]^ The quest for cheap and environmentally friendly industrial processes has triggered an extensive search for the far less well‐established main group congeners.^[3]^

**Scheme 1:**
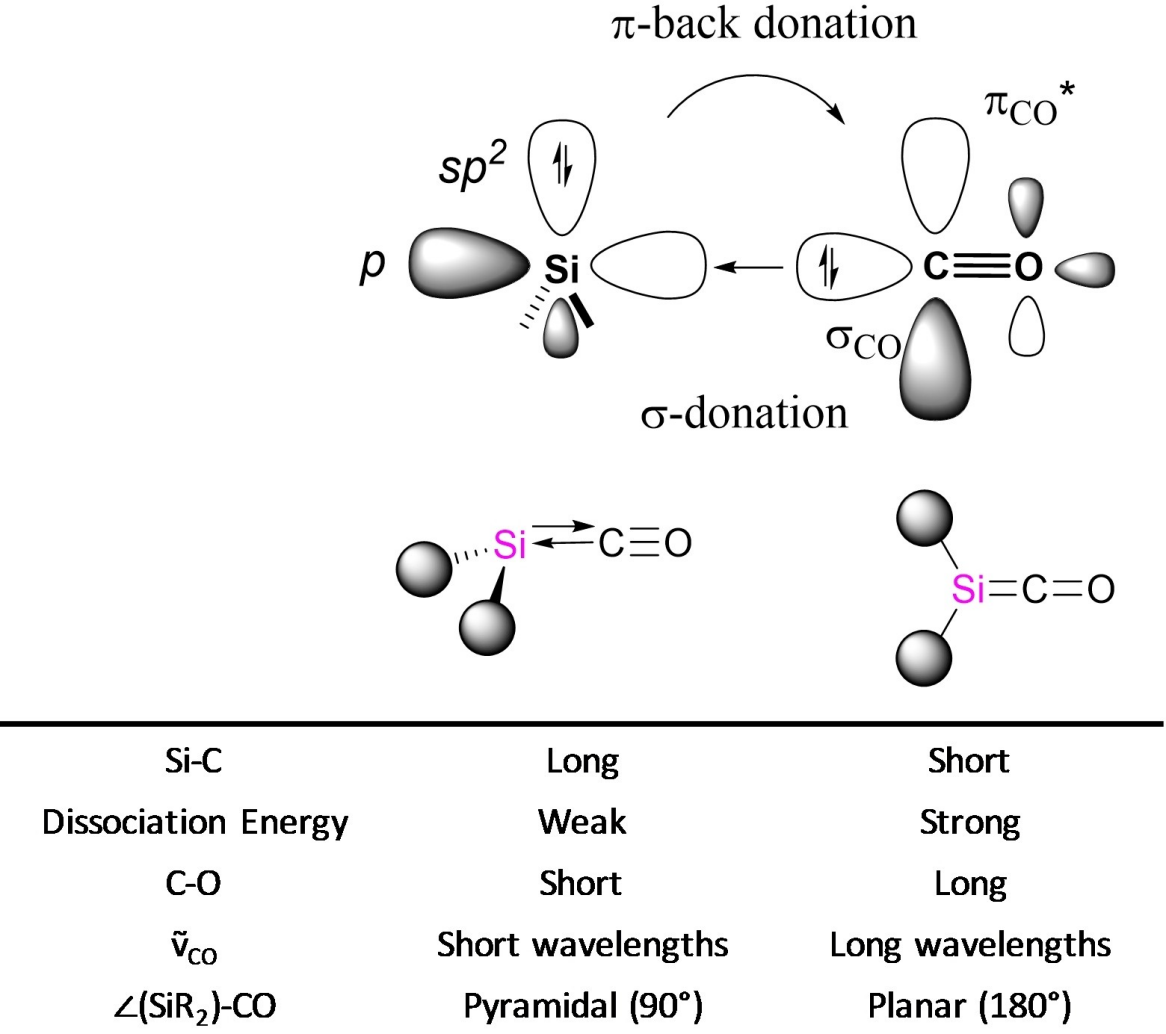
Schematic representation of the bonding interactions in silylene carbonyl complexes. Structural and spectroscopical properties.

Till now, various types of stable carbon‐ and silicon‐containing compounds have been synthesized and structurally characterized that demonstrate the ability to encompass similar chemical bonding and reactivity patterns as transition metals.^[4]^ In particular, group 14‐carbonyl complexes have been pursued for decades. Carbonylation of carbenes have been shown to yield stable ketenes when the singlet triplet gap is sufficiently low.^[5]^ On the contrary, the number of studies on stable silylene‐carbonyl complexes are still limited. In 1988, Chu *et al*. reported the upper limit rate constant for the reaction between SiH_2_ and CO to yield H_2_Si−CO (**1**),^[6]^ later re‐evaluated by Becerra *et al*.^[7]^ Independently, Arrington *et al*. provided evidence that the reaction of SiMe_2_ with CO yields Me_2_Si−CO (**2**) species, only stable below −196 °C.^[8]^ In addition, West *et al*. concluded that Me_2_Si−CO and MesRSi−CO (Mes=mesityl; R=Mes, ^*t*^Bu, or 2,6‐^*i*^Pr_2_C_6_H_3_O) are indeed to be considered as Lewis acid‐base complexes.^[9]^ Later, Jutzi and co‐worker claimed that silicocene Cp*_2_Si can also form carbonyl complexes, which was spectroscopically observed in a liquid xenon high pressure cell at −100 °C.^[10]^ Based on the IR and UV‐vis spectra, the structure was interpreted as a pyramidal silicon atom with a weak Si−CO interaction, in contrast to their stable carbon analogues which adopt a planar structure.^[11]^ The analysis of the bonding situation with quantum chemical methods showed that the Si−CO bond consists of a strong Si←CO *σ*‐donation and weaker Si(*sp* ^*2*^)→CO *π*‐backdonation,^[12]^ which resembles the corresponding transition metal bonding scheme as described by the Dewar‐Chatt‐Duncanson (DCD) model (Scheme 1).^[13]^ According to this chemical bonding model, a stronger Si(*sp* ^*2*^)→CO *π*‐back donation orbital interaction must lead to a shorter Si−C bond lengths, a longer C−O bond length, and hence a bathochromic shift of the CO stretching frequency in the IR spectrum. In the extreme case, the pyramidal arrangement is lost, leading to a trigonal‐planar structure with a strong Si=C bond, which would be better described as a silaketene. Nonetheless, silaketenes have been calculated to be only a transition state on the potential energy surface.[^12a^, ^12b^, ^12c^]

**Scheme 2:**
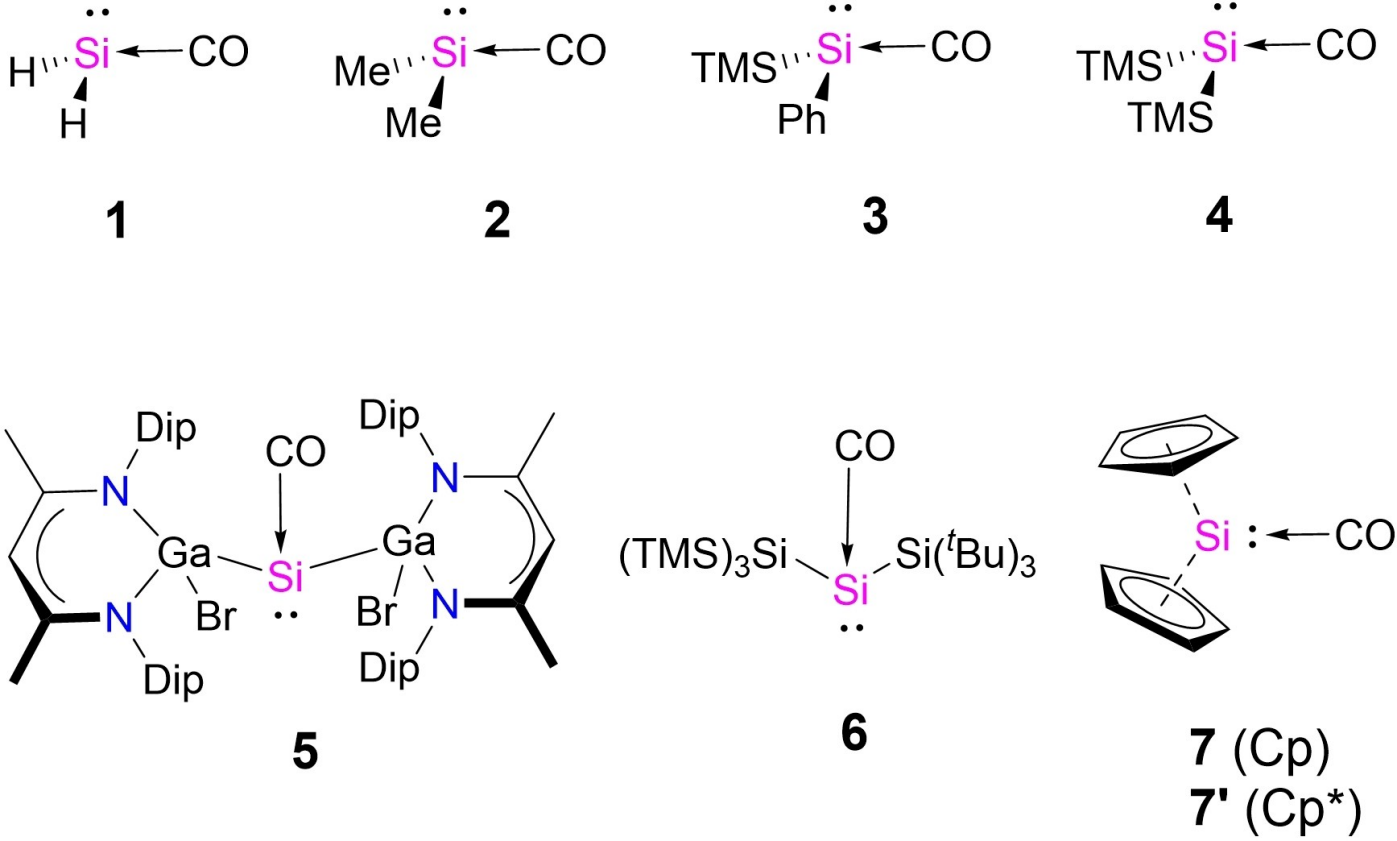
Molecular structures of the silylene‐carbonyl complexes considered in this study. Dip = 1,3‐diisopropylphenyl, TMS = trimethylsilyl; ^*t*^Bu = *tert*‐butyl.

Until very recently, isolable silylene‐carbonyls complexes seemed to be inaccessible in view of poor orbital stabilization which is otherwise known from *d*‐block transition metals.^[14]^ As a consequence, all attempts to accomplish silylene‐carbonyls resulted in reductive cleavage of carbon monoxide.^[15]^ Two landmark experimental studies, however, recently defied this paradigm by using a suitable substituent pattern at the Si center. By shielding the silicon atom with two bulky and strongly electron‐donating gallium‐based *β*‐diketiminate (L(Br)Ga) substituents^[16]^ Schulz, Schreiner and co‐workers isolated silylene‐carbonyl compound (**5**, Scheme 2). Shortly after, Inoue and co‐workers obtained the related complex **6** using bulky silicon‐based substituents.^[17]^ Notably, the significant stability allowed unambiguous characterization by single‐crystal X‐ray diffraction and IR spectrometry. Interestingly, the observed molecular structures exhibit a strong pyramidal arrangement of the central silicon (**5** 94.7°, and **6** 103.7°), indicating a weak *π*‐back donations. However, the C−O bond distances are significantly elongated compared to free CO, i. e. **5** 1.136 Å, **6** 1.153 Å, and free CO 1.128 Å. Indeed, the CO stretching frequency in the IR spectra show a bathochromic shift (*ṽ*=1945 cm^−1^ and 1908 cm^−1^ for **5** and **6**, respectively; vs. free CO, *ṽ*=2143 cm^−1^), in line with a strong donor‐acceptor interaction. Evidently, these novel Si−CO are extraordinary stable but the current chemical bond model lead to an ambiguous interpretation of the structure and spectroscopical properties.

Given that the stabilization of small *p*‐block fragments by dative ligands constitutes a very exciting topic in the current synthetic and theoretical chemistry. In the work presented herein, we perform computational calculations to re‐evaluate the chemical bonding of silylene‐carbonyl complexes, to pinpoint the driving forces behind the binding interaction of these intriguing compounds. We show that, in addition to the already discussed donation and back donation orbital interaction (Scheme 1), there is a non‐negligible second Si→CO *π*‐back donation. We demonstrate that the key effect from the gallium‐ and silicon‐based ligands is to strengthen such orbital interaction.

## Results and Discussion

**Figure 1:**
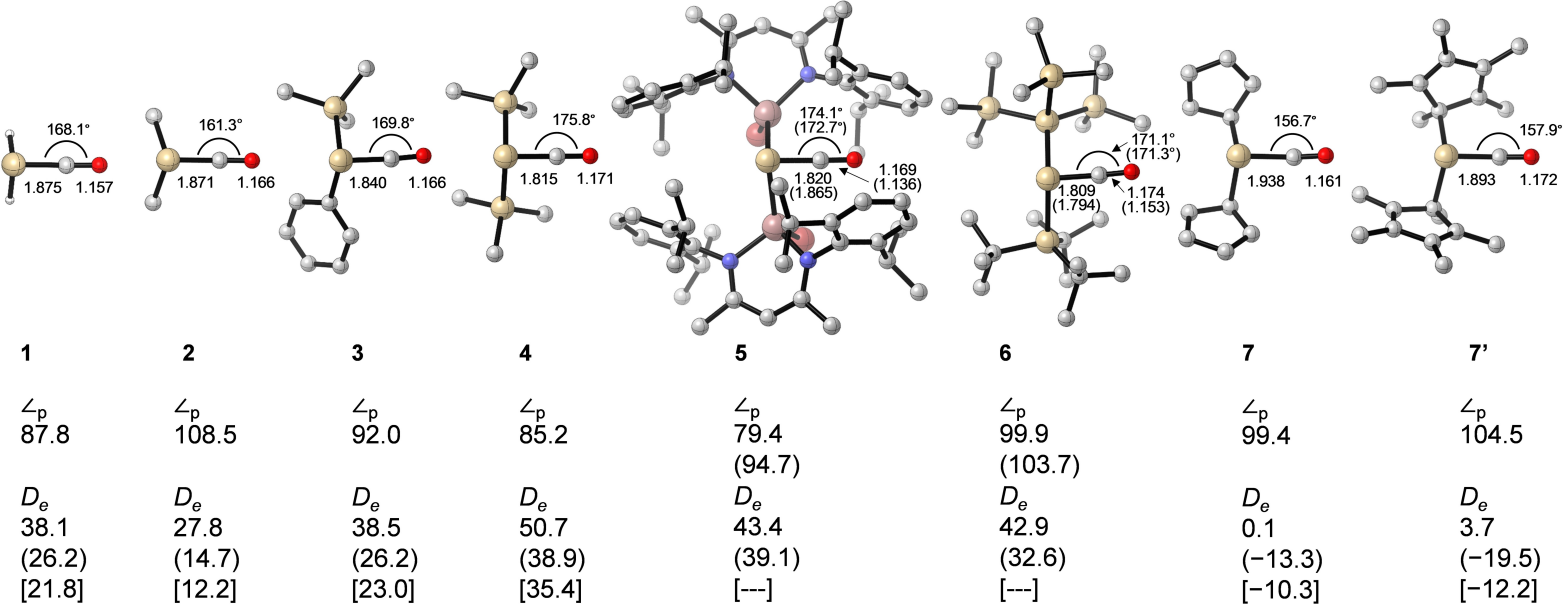
Optimized silylene−carbonyl complexes with selected bond distances in [Å] and bond angles in [°] at BP86‐D3(BJ)/def2‐SVP, experimental data (in parentheses) from refs. [16] and [17]. **7’** was optimized without dispersion corrections (see Supporting Information). The pyramidalization angle (∠_p_) has been taken as the angle between the SiR_2_ plane and SiCO plane. Dissociation energies (*D_e_*) in [kcal/mol] at the BP86‐D3(BJ)/def2‐TZVPP (M06‐2X‐D3/def2‐TZVPP) [LCCSD(T)/cc‐pVTZ] level of theory. Hydrogen atoms except for **1** were omitted for clarity.

Figure 1 shows the calculated equilibrium geometries of the experimentally‐known silylene‐carbonyl complexes outlined in Scheme 2. All structures have a singlet ground state with different degree of pyramidalization of the silicon centers. The calculated values for the Si−C bond length at the BP86‐D3(BJ)/def2‐SVP level are between 1.809 to 1.938 Å, which is in good agreement with previous theoretical calculations,[^12^, ^16^, ^17^] and experimental structures.[^16^, ^17^] Note, however, that silicocene compounds Cp_2_Si−CO (**7**) and Cp*_2_Si−CO (**7’**) behave differently from the other complexes. Our calculations predict a very long Si−C bond lengths 1.938 Å for **7** and 1.893 for **7’**, being the longest bond lengths of the considered series. Moreover, the dissociation energies (*D_e_*) reveal very labile complexes **7** (0.1 kcal/mol) and **7’** (3.7 kcal/mol), which are prone to dissociate. Similar results were obtained at different level of theory (see Table S1 and Figure S1 in the Supporting Information). While for some methods lead to minimum for the silylene‐CO complex, others like M06‐2X functional and MP2 methods suggest no coordination, despite all attempts carried out to find a minimum with the CO coordinated to Si atom. Nonetheless, such observations agree with the small experimental bathochromic shift of **7’** IR spectrum (*ṽ*=2065 cm^−1^), compared with the free CO (*ṽ*=2143 cm^−1^).^[10]^ Worth mentioning, free silicocene present a bent structure where all Si−C(Cp) bond length are different being 2.384 Å the shortest and 2.831 the longest Å for **7**, as described elsewhere.^[18]^ The complexation with CO causes significant geometrical changes observed on the substantial shortening of one of the Si−C(Cp) bond lengths 2.031 Å, while the other Si−C(Cp) becomes longer up to 3.410 Å. Similar geometrical changes have been reported for the coordination of *N*‐heterocyclic carbenes to stannocenes and plumbocenes.^[19]^

Besides, the Si−C bond lengths for the remaining complexes **1**–**6** are between 1.809 and 1.871 Å, and fall in the typical bond lengths of single and double bonds (1.91 Å and 1.74 Å, respectively).^[20]^ All C−O bonds are longer than in free CO (1.123 Å), which corresponds to the experimentally observed bathochromic shift of the CO stretching frequency in the IR spectra (*ṽ*=1945 cm^−1^ and 1908 cm^−1^ for **5** and **6**, respectively; vs. free CO, *ṽ*=2143 cm^−1^). This can easily be understood by *π*‐back donation, which results in a population of the CO's π*‐antibonding orbital and hence a weakening of the bond, as proposed for the vibrational spectra of carbonyl transition metals complexes, as sketched in Scheme 1.^[21]^

The Si−C−O bond angles are deviating from linearity and range from 156 to 175°. In fact, as can be seen from the pyramidalization angle (∠_p_), the bonding plane of the silylene fragment is tilted towards CO thus allowing for stronger Si→CO *π*‐back donation from the *sp* ^*2*^ orbital into the π*_CO_. Based on the chemical bonding depicted in Scheme 1, a wider ∠_p_ angle should lead to shorter Si−CO and longer C−O bond lengths. Such anticipated structural changes, however, can be ruled out by inspection of the simple examples **1**, **2** and **4**. H_2_Si−CO shows a Si−C bond length of 1.875 Å, and a ∠_p_ of 87.8°. Changing hydrogen atoms (**1**) by more electron donating methyl groups (**2**) slightly decreases the Si−CO bond length to 1.871 Å and the central Si atom pyramidal angle becomes wider ∠_p_=108.5°. As expected, longer C−O bond length are obtained 1.157 (**1**) and 1.166 Å (**2**). The replacement of methyl group by trimethylsilyl groups (**4**) decreases the Si−CO bond length (1.815 Å) and increases the C−O bond lengths (1.171 Å). In contrast to the expected pyramidalization, the ∠_p_ angle becomes acute, namely 85.2°. Considering the silicocenes‐carbonyl complexes, the Si pyramidalization is comparable to **2**, but the Si−CO bond lengths are very long and the C−O bond lengths short. Such a simple structure comparison points out towards a missing interaction in the chemical bonding model of Scheme 1.

More considerable discrepancies can be found for dissociation energies. Figure 1 also gathers the bond dissociation energies (*D_e_*) for the Si−CO bond cleavage at the BP86‐D3(BJ)/def2‐TZVPP and LCCSD(T)/cc‐pVTZ level of theory. As anticipated, it does not seem to be a simple correlation between Si−C and C−O bond length and the bond dissociation energies. This is yet another example that a shorter bond length does not necessarily mean a stronger bond.^[22]^ This can be already seen for H_2_Si−CO (**1**) and Me_2_Si−CO (**2**) systems, where the dissociation energy value is 10 kcal/mol smaller for the latter, while the Si−CO is 0.004 Å shorter. The calculations at different levels of theory agree with *D_e_* difference of 10 kcal/mol and Si−CO up to 0.017 Å (Table S1). Indeed, one would expect a higher *D_e_* value for **2** compared to **1** since its structure is more similar to a silaketene structure. This necessarily leads to the conclusion that an additional type of interaction is relevant in such complexes. Notably, when the substituent groups are gallium (**5**) or silicon (**6**) derivatives, the Si−C bond distances are short, and the C−O bond distances long. Indeed, the calculated values suggest a high thermodynamic stability of Si−CO bonds in compounds **5** and **6** (43.4 and 42.9 kcal/mol, respectively).

Next, we analyzed the electronic structures of **1**–**6** by Natural Bond Orbitals (NBO) analysis. Table 1 gathers the calculated natural atomic partial charges (Q) and Wiberg bond orders (P) of the Si−CO and C≡O bonds. The positive charge at Si is strongly related to the electronegativity of the substituents. For instance, in **1** the central Si atoms bears +0.22e, whereas for **2** the Si partial charge increases to +0.77e and for **3** and **4** the charge is +0.40e. The gallium and silicon based substituents lead to lower or even negative charges **5** (−0.21e) and **6** (+0.01e). Notably, the bond orders P(Si−CO) suggest a double‐bond character ranging from 1.21 (**1**) to 1.35 a.u. (**6**). In this context, the P(Si−C) in H_3_Si−CH_3_ is 0.89 a.u., H_2_Si=CH_2_ is 1.83 a.u., and for a triple bonded Si≡C has been reported to be 1.87–2.02 a.u.^[23]^ As become evident, the P(Si−CO) is higher when more *σ*‐donating substituents groups on the central Si. For instance, in the case of Me groups (**2**) is 1.22 a.u., while for TMS groups (**4**) is 1.32 a.u.

**Table 1:** NBO results for **1**–**7’** at BP86‐D3(BJ)/def2‐TZVPP level of theory: partial charges (Q, in e), Wiberg bond order (P, in a.u.), and second order interactions (Δ*E* ^*2*^, in kcal/mol).

	**Q(Si)**	**Q(CO)**	**P(Si−CO)**	**P(C≡O)**	**σ_(Si−CO)_ contribution**	**Δ*E*** ^***2***^ (sp^2^→ π*_CO_)	**Δ*E*** ^***2***^ (σ_RSi_⊥→π*_CO_)
1	+0.22	−0.01	1.21	2.14	Si(24.4 %)–C(75.6 %)	5.2	19.5
2	+0.77	−0.08	1.22	2.11	Si(23.6 %)–C(76.3 %)	20.8	7.8
3	+0.40	−0.11	1.23	2.06	Si(25.0 %)–C(75.0 %)	7.7	25.5
4	+0.40	−0.20	1.32	1.99	Si(26.1 %)–C(73.9 %)	2.5	45.4
5	−0.21	−0.27	1.35	1.91	Si(24.9 %)–C(75.1 %)	<0.5	53.8
6	+0.01	−0.22	1.34	1.96	Si(23.2 %)–C(76.8 %)	<0.5	32.3
7	+0.87	−0.08	1.03	2.12	Si(21.1 %)–C(78.9 %)	7.7	9.0
7’	+0.92	−0.21	1.11	2.00	Si(22.3 %)–C(77.6 %)	6.5	8.9

Table 1 shows that the CO fragment always carries a negative partial charge between −0.01e to −0.27e, which indicates that the CO moiety is the acceptor fragment. The trend observed in P(Si−CO) may come from a stronger interaction between the silylene and CO, which agrees with the increasing partial negative charge on the CO moiety. The bond orders for the Si−C bonds suggest that they are single bonds with *π*‐back bonding. The bond order for the C≡O bond becomes smaller, ranging from 2.14 to 1.96 a.u., as the bond order of the Si−C bond increases. This suggests that the net charge transfer comes from *σ*‐donation and *π*‐back‐donation. Nonetheless, the pyramidalization angle (∠_p_) cannot be directly related to these orbital interactions.

The NBO analysis leads to a Lewis structure with a strongly polarized covalent description of Si−C σ‐bond (σ_(Si−CO)_) and a lone pair on the Si atom. Table 1 presents the polarization of σ_(Si−CO)_ where C contributes with (74–79 %) and Si with the remaining (21–26 %). This bond is formed from *p*‐rich (90 %) hybrid orbital of the Si atom and *s*‐rich (67 %) hybrid orbital of the C atom, as summarized in Table S2. The charge distribution then suggests that the silylene‐carbonyl complexes are best described in terms of the charged fragment Si^+^−(CO)^−^. This is another example of a polarity below the standard criterion for bond orbitals by the NBO method, which ends in a σ(Si−CO) bond. Similar situations have been recently discussed for alkaline earth oxides and imides.^[24]^ Such a representation does not agree with the conventional understanding of the Si−CO chemical bond. Therefore, we have enforced the Lewis representation with a CO *σ* lone pair, but the Non‐Lewis density increases from 0.56e to 1.05e (Table S3).

Despite this, we have assessed the interaction between the silylene and the carbonyl moieties by the second‐order perturbation method within NBO. Interestingly, the strongest interaction among donor‐acceptor Lewis‐type NBOs identified for the *π*‐back donation from the R−Si *σ‐*bonding orbital (σ_RSi_^⊥^) into the π*_CO_ with an exception for compound **2**. The estimation of energetic importance for *σ*_RSi_^⊥^→π*_CO_ interaction determines the stabilization energy associated with this delocalization to range from 7.8 to 53.8 kcal/mol. It has also emerged that *σ*_RSi_^⊥^→π*_CO_ pronounced the most in the series with silicon and gallium substituents reaching the highest values of 45.4 and 53.8 kcal/mol for compounds **4** and **5**, respectively. On the other hand, the expected lone pair donation from the Si atom into the π*_CO_ (*sp* ^*2*^→π*_CO_) is predicted to be weaker for most of the complexes (0.5–7.7 kcal/mol), but especially **2** showed a strong interaction (20.8 kcal/mol), which agrees with the ∠_p_ angle. In order to quantify the CO donation into the *p*‐empty orbital of Si, we have used the enforced Lewis structures. The second‐order perturbation theory predicts a high interaction that counts to 265.5 kcal/mol for **1** (Table S4). This value is too high to consider this perturbation method an accurate approach for the discussion of the interaction.[^24^, ^25^]

More detailed information about the nature of the Si−CO bonds in the silylene‐carbonyl complexes and the best representation of the bonds in terms of Lewis structures are provided by the results of the Energy Decomposition Analysis (EDA) method.^[22]^ EDA has proven to be a useful tool to assess the nature of the chemical bond in main group compounds and transition metal compounds.^[26]^ Nonetheless, a recent discussion has been placed about the path function nature of the energy components.^[26c]^ Within EDA scheme, the bond formation between two (or more) fragments is divided into three steps (for further details, see the Supporting Information.

Table 2 shows the numerical results of the calculations where silylene and CO are both in singlet reference state in reacting fragments. Thus, the *σ_CO_* lone pair interacts with the empty *p*‐orbital of the silylene, and also the silylene lone pair (*sp* ^*2*^) with the π*_CO_ antibonding orbital, as displayed in Scheme 1. The interaction energies Δ*E*_int_ follows the same trend as the dissociation energy since the preparation of the fragments (Δ*E*_prep_) does not carry particular energy penalties for most of the compounds. However, for bulky substituent, the deformation energies of the silylenes are non‐negligible (**5**; 12.8 kcal/mol, and **6**; 9.0 kcal/mol). Δ*E*_int_ showed to be larger for silicon and gallium based substituents than those with hydrogen and methyl groups. In the case of silicocenes, Δ*E*_int_ values are only −16.8 (**7**) and −20.4 kcal/mol (**7**’). Notably, the deformation energies are the 21.3 and 24.7 since the interaction of CO with Si leads to big geometrical distortions when is compared to the free silicocenes. The dissection of the Δ*E*_int_ reveals that the bonding interactions are on average 42 % ionic and 2 % attractive dispersion interactions for the small silylenes, while in **5** and **6** the dispersion interactions have slightly higher relevance, ∼5 %.

**Table 2:** EDA‐NOCV results of the Si−C bond in silylene−carbonyl complexes **1**–**6**at BP86‐D3(BJ)/TZ2P. All values are in kcal/mol.^[a]^

	**1**	**2**	**3**	**4**	**5**	**6**	**7**	**7’**
Δ*E*_int_	−42.2	−32.9	−41.0	−52.5	−54.9	−51.4	−16.8	−20.4
Δ*E*_Pauli_	139.2	195.8	190.5	184.4	202.9	213.1	171.4	234.4
Δ*E*_elstat_^[b]^	−76.3 (42.1 %)	−102.9 (45.0 %)	−100.2 (43.3 %)	−97.6 (41.2 %)	−104.0 (40.3 %)	−110.7 (41.9 %)	−86.0 (45.7 %)	−115.8 (45.5 %)
Δ*E*_disp_^[b]^	−1.9 (1.1 %)	−2.9 (1.3 %)	−5.1 (2.2 %)	−5.9 (2.5 %)	−14.6 (5.7 %)	−9.1 (3.5 %)	−3.7 (1.9 %)	−6.0 (2.3 %)
ΔE_orb_^[b]^	−103.1 (56.8 %)	−122.9 (53.7 %)	−126.2 (54.5 %)	−133.4 (56.3 %)	−139.2 (54.0 %)	144.6 (54.7 %)	−98.6 (52.4 %)	−133.0 (52.2 %)
Δ*E*_orb1_ (*σ*_CO_→ *p*_Si_)^[c]^	−50.5 (49.0 %)	−39.9 (32.5 %)	−47.4 (37.6 %)	−60.8 (45.6 %)	−63.4 (43.8 %)	−50.5 (34.9 %)	−48.5 (49.2 %)	−74.4 (56.0 %)
Δ*E*_orb2_ (*sp* ^*2*^→ π*_CO_)^[c]^	−36.6 (35.5 %)	−70.3 (57.2 %)	−54.5 (43.2 %)	−38.0 (28.5 %)	−39.9 (27.6 %)	−63.5 (43.9 %)	−35.3 (35.8 %)	−33.0 (24.8 %)
Δ*E*_orb3_ (*σ*_RSi_^⊥^→ π*_CO_)^[c]^	−10.0 (9.7 %)	−7.8 (6.3 %)	−16.1 (12.7 %)	−23.9 (17.9 %)	−23.7 (16.4 %)	−18.9 (13.1 %)	−8.8 (9.0 %)	−17.2 (12.9 %)
Δ*E*_orb rest_^[c]^	−5.9 (5.7 %)	−4.9 (4.0 %)	−8.2 (6.5 %)	−10.7 (8.0 %)	−12.3 (8.5 %)	−11.7 (8.1 %)	−6.0 (6.1 %)	−8.4 (6.3 %)
Δ*E*_prep_	4.8	6.1	3.5	2.4	12.8	9.0	21.3	24.7
*D_e_*	37.4	26.8	37.5	50.1	42.1	42.4	−4.4	−4.3

[a] All calculations were performed on the BP86‐D3(BJ)/def2‐SVP optimized structures. [b] The value in parenthesis gives the percentage contribution to the total attractive interactions Δ*E*_elstat_+Δ*E*_orb_+Δ*E*_disp_. [c] The value in parenthesis gives the percentage contribution to the total orbital interaction term.

Deeper insights into the nature of the covalent interaction are available from the combination of EDA with natural orbitals for chemical valence calculations (EDA‐NOCV).^[27]^ This method deconstructs the orbital term (Δ*E*_orb_) into components (Δ*E*_orb_^*ρ(i)*^) that provide an energetic estimation of a given deformation density (*ρ*_*(i)*_), which is related to a particular electron row channel, and consequently the amount of charge transferred, Δ*q*_(i)_=|ν_(i)_|, for the bonding between the interacting fragments. The most interesting results are obtained by breaking down the orbital (covalent) term Δ*E*_orb_, which contributes on average about 55 % to the total attraction of the Si−CO bonds, into the individual pair‐wise orbital interactions.

Table 2 shows that, in the silylene‐carbonyl compounds, the main orbital contributions consist in the *σ*‐donation and a *π*‐back donation as sketched in Scheme 1. The sum of such orbital interaction counts around 70 % of the total orbital interaction. The comparison of the values faithfully explains the Si−C−O angle. The stronger the Si→CO *π*‐back donation the more tilted is the silylene moiety towards the CO. Noteworthy, the dimethylsilylene (**2**) has the weakest *σ*‐donation and the strongest *π*‐back donation of the series. This counter effect explains the lower pyramidalization of the central Si atom. However, the sum of these two interactions is also the highest of the series for **2**, which appears to be in conflict with the significant low dissociation energy (*D*_e_) in Figure 1. A closer inspection of the three most important pairwise contributions to Δ*E*_orb_ shows that there is an additional Si→CO *π*‐back donation, which comes from the plane of the silylene moiety.

**Figure 2:**
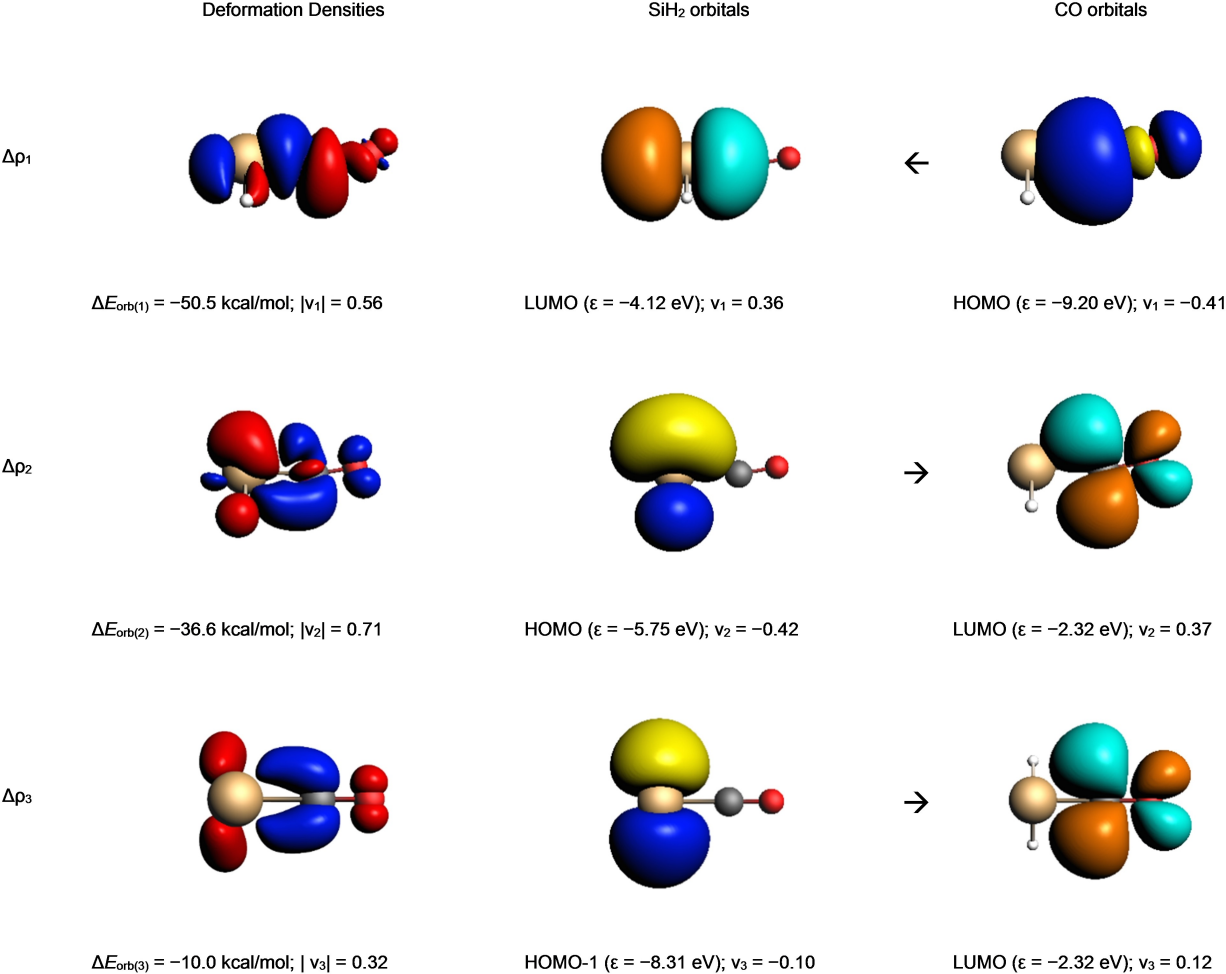
Plot of deformation densities Δ*ρ*_1–3_ (isovalue=0.003) of the pairwise orbital interaction and shape of the most important occupied and vacant orbitals (isovalue=0.03) in silylene−carbonyl complex **1** with the orbital interaction energies Δ*E*_orb_ (in kcal/mol) and their eigenvalues *ν* (in e). For the deformation densities, the direction of the charge flow is red→blue. The eigenvalues *ν* indicate the amount of donated (negative numbers) and accepted charge (positive numbers). The occupied orbitals are shown in yellow and blue for the different phases, while the unoccupied orbitals are in cyan and orange.

Figure 2 displays the shape of the deformation densities *ν*_1_–*ν*_3_, which give the charge flow and the most important fragment orbitals which are involved in the pairwise donor‐acceptor bonding for the complex H_2_Si−CO. The color coding red to blue illustrates the direction of the charge flow. Thus, the result of calculations can be easily understood through visualization. The results for the other systems **2**–**7** are similar and are shown in Figures S4–S11 in the Supporting Information. EDA‐NOCV reveals a pair‐wise interaction between HOMO‐1 for the silylene and the LUMO of CO (π*_CO_). This interaction becomes particularly strong when *σ*‐donating substituents are attached to Si center in the case of compounds **5** and **6**.

**Scheme 3:**
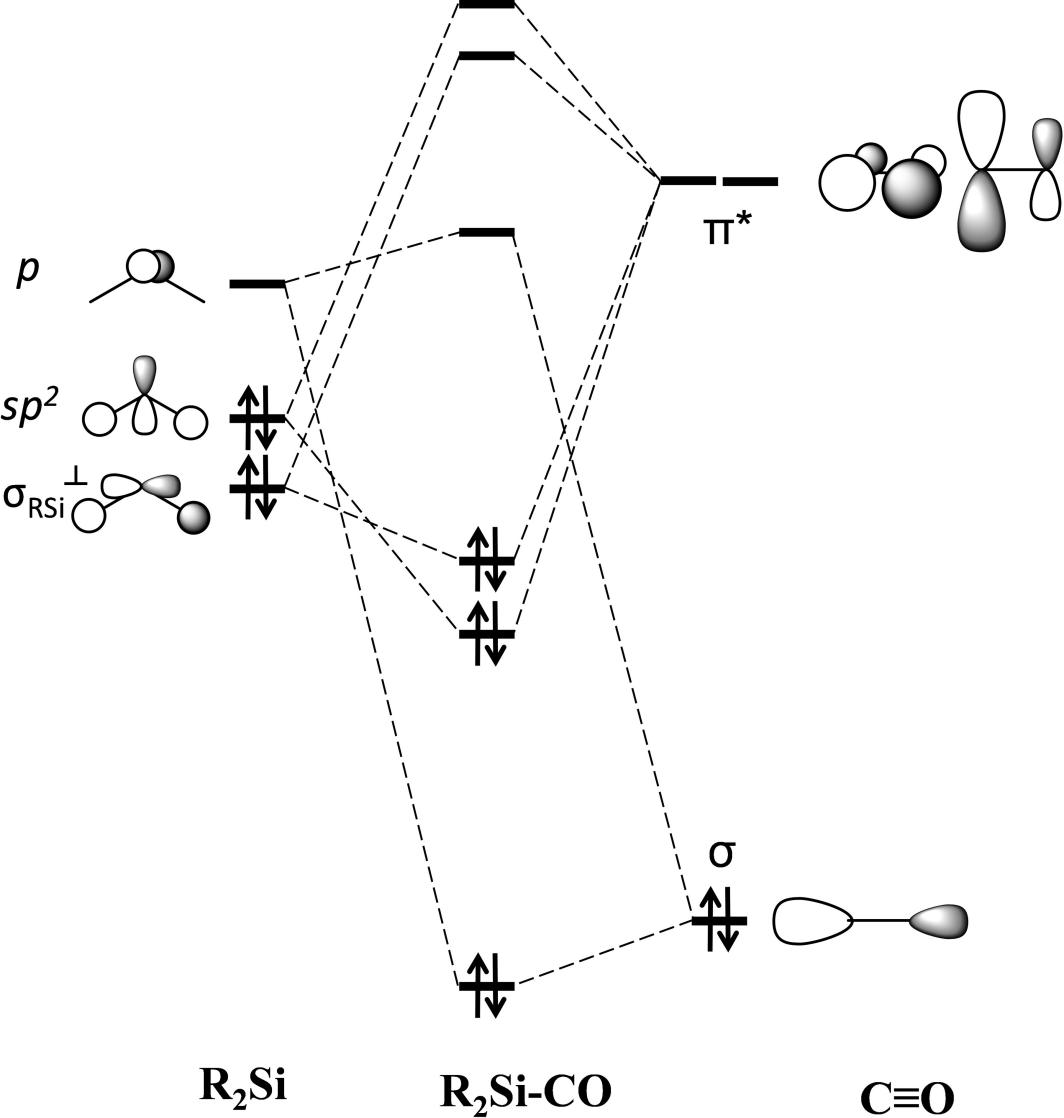
Schematic representation of the MO interactions of silylenes with CO.

Scheme 3 shows the complete orbital diagram of the orbital interactions between the silylene and carbon monoxide fragments. As suggested by the EDA, these interactions can be expected to give the largest contributions to the Δ*E*_orb_ term. Although the stabilization arises from the Si←CO *σ*‐donation and Si→CO *π*‐back donation, it can be reasonably argued that a significant contribution comes from HOMO‐1 orbital (*σ*_RSi_^⊥^) of silylene into the perpendicular π*_CO_. This orbital is expected to decrease in energy with increasing bulkiness of the groups, since broader R−Si−R angles leads to a better overlap of the constituting on Si and R substitutes. However, the stronger *σ*‐donating character of the substituents raises its energy so it contributes up to ∼20 % on the total orbital interaction. Considering such a chemical bonding model essentially explain that strong Si pyramidalization also means strong *π*‐back donation. Therefore, it is expected to have shorter Si−C and longer C−O bond distances. This unrealized Si (*σ*_RSi_^⊥^)→π*_CO_ back donation orbital interaction prevents the Si center from the planar arrangement of the silaketene structure by providing significant stabilization.

**Scheme 4:**
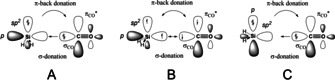
Schematic representation of the three bonding models possible for the silylene−CO complexes. (A) donor‐acceptor bond: *σ*_CO_→*p*_Si_ *σ*‐donation and Si(*sp* ^*2*^)→π*_CO_ *π*‐back donation; (B) electron‐sharing double bond between SiH_2_ and CO in the triplet state; (C) donor‐acceptor bond: *σ*_CO_→Si(*sp* ^*2*^) *σ*‐donation and *p*_Si_→π*_CO_ *π*‐back donation.

To gain more quantitative insight into the relationship between the orbital interaction (Δ*E_orb_*) and the pyramidalization angle (∠_p_), we have performed a relaxed scan from 90° to 180° for compound **1** (see Figure S12 in the Supporting Information). The electronic energy of the planar structure in *C_2v_* symmetry is 15.5 kcal/mol with respect to the optimized structure shown in Figure 1. We have applied EDA‐NOCV alone the pyramidalization angle, considering a possible change of configuration for the silylene and the carbonyl fragments. The three bonding situations A−C, which are shown in Scheme 4, have been examined by changing the configuration of the SiH_2_ and CO. The calculation for model A uses the occupations SiH_2_ (a_1_^8^ a_2_^0^ b_1_^4^ b_2_^2^) and CO (a_1_^6^ a_2_^0^ b_1_^2^ b_2_^2^), as explained in the introduction and summarized in Table 2. For model B, with the electron sharing double bond Si=CO, we employed the fragments in their electronic triplet state SiH_2_ (a_1_^7^ a_2_^0^ b_1_^4^ b_2_^3^) and CO (a_1_^5^ a_2_^0^ b_1_^2^ b_2_^3^). Finally, for model C, we occupied the *p* orbital of Si and unoccupied the *sp* ^*2*^ giving a configuration SiH_2_ (a_1_^6^ a_2_^0^ b_1_^4^ b_2_^4^) and CO (a_1_^6^ a_2_^0^ b_1_^2^ b_2_^2^). The comparison between these chemical bonding models can be related to the orbital interaction term within the EDA scheme since it is related to the relaxation of the intermediate state into the final molecular configuration. The lower the absolute value of Δ*E_orb_*, the better is the representation of the chemical bonding.^[26]^ This criterion has been taken to underline the bonding situation of many systems, despite the limitations that have been recently highlighted.^[26]^

**Figure 3:**
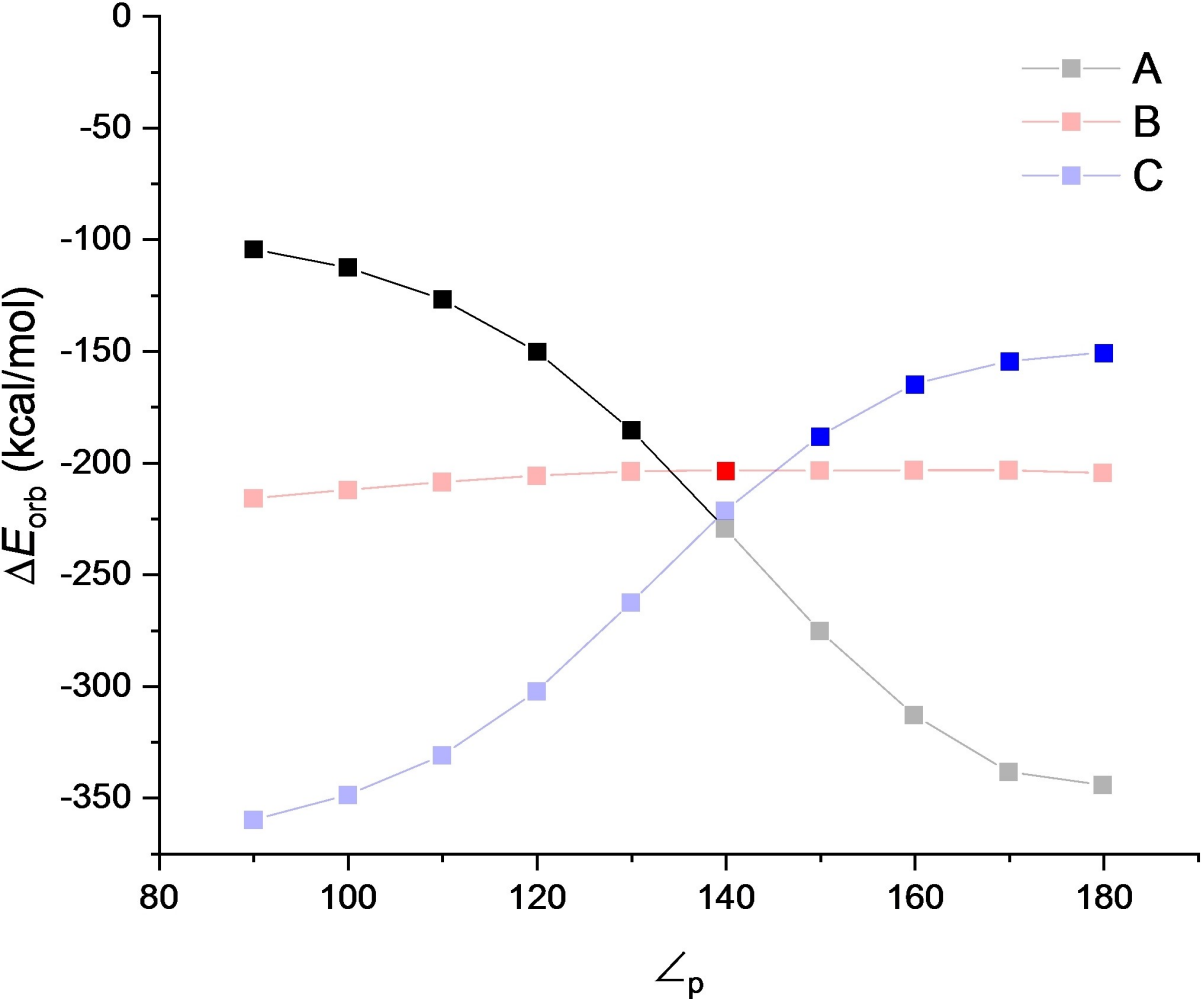
Calculated EDA (BP86‐D3(BJ)/TZ2P) orbital term Δ*E_orb_* [kcal/mol] values for the interaction between SiH_2_ and CO with different electronic states and different pyramidalization angle (∠_p_). Full symbols represent bonding models.

**Figure 4:**
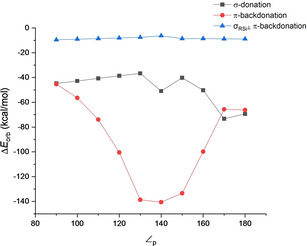
Dissection of the orbital term Δ*E_orb_* into *σ*‐donation, *π*‐backdonation and σ_RSi_^⊥^ π‐backdonation for different pyramidalization angle (∠_p_) of compound **1**.

Figure 3 shows the calculated Δ*E_orb_* for the three chemical bonding situations along the pyramidalization angle. The numerical values are summarized in Tables S5–S7 in the Supporting Information. The EDA calculations indicate that the bonding model A is the best representation when ∠_p_ is between 90° to 130°, which model C is better for ∠_p_ between 150° to 180°, and model B is representative only at ∠_p_ 140°. The dissection of the orbital term taking into account this change of configuration is depicted in Figure 4. As the pyramidalization angle becomes wider (from 90° to 140°), the (Si)*sp* ^*2*^→ π*_CO_ increases since the overlap between the orbitals is favored. Once the configuration changes at 150° the π‐backdonation involves the (Si)*p*→ π*_CO_, which decreases to reach 180°. On the contrary, σ‐donation initially (90°‐140°) decreases since the pose of the fragments precludes the interaction between the *σ_CO_* and the Si(*p*). After 140°, the interaction becomes more favorable due to a better interaction between the *σ_CO_* and now Si(*sp* ^*2*^) orbitals. Finally, the Si (*σ*_RSi_^⊥^)→π*_CO_ orbital interaction constantly decreases from 90° to 180° with a change of ca. 4 kcal/mol. This small change in comparison with the other interaction is related to the flexibility of compound **1** since the H−Si−H angle 96° when ∠_p_ is 90°, while at ∠_p_ is 180°, the H−Si−H angle is 130°. Such structural changes diminish the effect of pyramidalization on the Si (*σ*_RSi_^⊥^)→π*_CO_ orbital interaction.

**Scheme 5:**
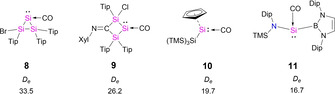
Predictive Si‐CO species. Dissociation energies (*D_e_*) in [kcal/mol] at the BP86‐D3(BJ)/def2‐TZVPP//BP86‐D3(BJ)/def2‐SVP level of theory. Dip = 1,3‐diisopropylphenyl, Tip = 2,4,6‐triisopropylphenyl, Xyl = 2,6‐dimethylphenyl.

As a consequence of this study, there are key features to broaden the scope of stable silylene‐carbonyl compound examples. Bulky groups must be considered to provide kinetic stability to the silylene center, aside from the needed weight to keep it in the solid‐state or solution. Nonetheless, groups like cyclopentadienes would lead to big deformation penalties, while those with electronegative character would lead to weak orbital interaction. This can be exemplified with literature‐known compounds like **10** ^[28]^ or **11** ^[15d]^ in Scheme 5. The results reflect previously observed trends in the series **1**–**7** and support the importance of Si atoms in the structure of the ligand. The calculated bond dissociation energies (*D_e_*) for the Si−CO bond cleavage are 19.7 kcal/mol and 16.7 kcal/mol for **10** and **11**, respectively. Then, to increase the interaction between the CO and the silylene there must be strong *σ*‐donating groups attached to the Si center. This is the case of compounds **8** ^[29]^ and **9**,^[30]^ which display stronger interactions with CO, given the enhanced interaction by the Si−Si σ‐bonds.

## Conclusion

The extraordinary stability of the recently accomplished on Si−CO species is a direct consequence of the strong *σ*‐donation character of the substitution pattern, silicon or gallium. However, the accepted bonding description consisting of a *σ*‐donation from the *σ_CO_* orbital into the empty *p*‐orbital of Si, and a *π*‐backdonation from the Si *sp* ^*2*^ lone pair into the π*_CO_ orbital is not complete enough to describe the structural changes and bond dissociation energy trends. A fundamental flaw is observed when the pyramidalization angle is related to the strength of Si→CO *π*‐backdonation. Our computational study based on energy decomposition analysis reveals that there is another previously unrecognized perpendicular *π*‐backdonation (Si(*σ*_RSi_^⊥^)→π*_CO_) term that plays a crucial role on interaction between silylene and carbon monoxide. This chemical bond model can clearly explain the observed structures and serve as a guideline for further designing novel stable Si−CO species.

## Computational Details

All geometries were optimized without symmetry constraint within the DFT (density functional theory) framework using the BP86 functional^[31]^ in combination with the Grimme Dispersion corrections using the Becke‐Johnson damping function D3(BJ)^[32]^ and the Ahlrichs def2‐SVP basis function.^[33]^ These calculations were performed using the Gaussian 16 C01 software.^[34]^ The stationary points were located with the Berny algorithm^[35]^ using redundant internal coordinates. Analytical Hessians were computed to determine the nature of stationary points^[36]^ and to calculate the thermal corrections and entropy effects using the standard statistical‐mechanics relationships for an ideal gas.^[37]^ Additional calculations were performed using B3LYP,^[38]^ PBE0,^[39]^ wB97xd,^[40]^ and M06‐2X functionals,^[41]^ and at the MP2 level of theory.

We carried out the local couple cluster (LCCSD(T))^[42]^ levels of theory calculations by employing MOLPRO 2019.1^[43]^ software program package. Density fitting (DF) approximations have been used in this local method.^[44]^ The cc‐pVDZ basis set was used for carbon, silicon and hydrogen.^[45]^ In the density fitting calculations reported in this paper, we used the cc‐pVTZ/JKJIT and cc‐pVTZ/MP2FIT auxiliary fitting basis sets in the DF‐HF and DF‐LMP2 calculations, respectively. The LCCSD(T) calculations were carried out using Pipek‐Mezey localized orbitals.^[44]^ The domains were determined with the use of natural population analysis criteria, with TNPA=0.03.

The Wiberg Bond Indices (WBI)^[46]^ and NPA^[47]^ atomic partial charges have been calculated at the BP86‐D3(BJ)/def2‐TZVPP level using GENNBO7.0 programs.^[48]^

The bonding situation was investigated by the means of the Energy Decomposition Analysis (EDA) method.^[49]^ Within this method, the intrinsic interaction energy Δ*E*_*i*nt_ between two or fragments A and B, in the particular electronic reference state and in the frozen geometry AB, is divided into four main components [Eq 1].

(1) $$\Delta E{}_{\mathrm{int}}=\Delta E{}_{\mathrm{elstat}}+\Delta E{}_{\mathrm{Pauli}}+\Delta E{}_{\mathrm{orb}}+\Delta E{}_{\mathrm{disp}}\;$$

The term Δ*E_elstat_* corresponds to the classical electrostatic interaction between the unperturbed charge distributions of the prepared atoms (or fragments) and it is usually attractive. The Pauli repulsion Δ*E_Pauli_* is the energy change associated with the transformation from the superposition of the unperturbed wave functions (Slater determinant of the Kohn‐Sham orbitals) of the isolated fragments to the wave function *Ψ_0_* =*NÂ[Ψ_A_Ψ_B_]*, which properly obeys the Pauli principle through explicit antisymmetrization (*Â* operator) and renormalization (N=constant) of the product wave function. It comprises the destabilizing interactions between electrons of the same spin on either fragment. The orbital interaction Δ*E_orb_* accounts for charge transfer and polarization effects.^[50]^ In the case that the Grimme dispersion corrections^[32]^ are computed the term Δ*E_disp_* is added to the Equation (1). Further details on the EDA method can be found in the literature.^[51]^

The addition of Δ*E_prep_* to the intrinsic interaction energy Δ*E_int_* gives the total energy ΔE, which is ‐ by definition with opposite sign ‐ the bond dissociation energy *D_e_* [Eq. 3]:

(2) $$\Delta E\left(\text{-}D{}_{e}\right)=\Delta E{}_{int}+\Delta E{}_{prep}\;$$

The EDA−NOCV method combines the EDA with the natural orbitals for chemical valence (NOCV) to decompose the orbital interaction term Δ*E_orb_* into pairwise contributions. The deformation of the electron density *Δρ_k_* (r) originates from the mixing of the orbitals pairs *Ψ_k_* and *Ψ*_*‐k*_ [Eq. 3],

(3) $$\Delta \rho_{\mathrm{orb}}=\sum_{k}\Delta \rho_{\mathrm{orb}}\left(r\right)=\sum_{k=1}^{N/2}v_{k}\left[-\Psi_{-k}^{2}\left(r\right)+\Psi_{k}^{2}\left(r\right)\right]\;$$

In the EDA−NOCV scheme the orbital interaction term, ${\Delta E}_{orb}$ , is given by Equation 4,

(4) $$\Delta E_{\mathrm{orb}}=\sum_{k}\Delta E_{k}^{\mathrm{orb}}=\sum_{k=1}^{N/2}v_{k}\left[{-F}_{-k,k}^{\mathrm{TS}}+F_{k,k}^{\mathrm{TS}}\right]$$

in which $F_{-k,-k}^{TS}$ and $F_{k,k}^{TS}$ are diagonal transition state Kohn‐Sham matrix elements corresponding to NOCVs with the eigenvalues −ν_*k*_ and ν_*k*_, respectively. The ${\Delta E}_{k}^{orb}$ term for a particular type of bond is assigned by visual inspection of the shape of the deformation density Δρ_k_. The latter term is a measure of the size of the charge deformation and it provides a visual notion of the charge flow that is associated with the pairwise orbital interaction. The EDA−NOCV scheme thus provides both qualitative and quantitative information about the strength of orbital interactions in chemical bonds.

The EDA‐NOCV calculations were carried out with ADF2019.103.^[51b]^ The Slater type basis sets for all elements have triple‐ζ quality augmented by two sets of polarizations functions and one set of diffuse function (TZ2P). Core electrons were treated by the frozen‐core approximation. This level of theory is denoted BP86‐D3(BJ)/TZ2P.^[52]^ Scalar relativistic effects have been incorporated by applying the zeroth‐order regular approximation (ZORA).^[53]^

## Conflict of interest

The authors declare no conflict of interest.

## Supporting information

As a service to our authors and readers, this journal provides supporting information supplied by the authors. Such materials are peer reviewed and may be re‐organized for online delivery, but are not copy‐edited or typeset. Technical support issues arising from supporting information (other than missing files) should be addressed to the authors.

Supporting Information
