# Supplementary material for: Chemical Bonding in Silicon Carbonyl Complexes
Source: Chemistry. 2021 May 1;27(41):10601–9. doi: 10.1002/chem.202100493 (PMC8360033; doi:10.1002/chem.202100493)
Supplement: Supplementary file 1 — Supporting Information [file CHEM-27-10601-s001.pdf]

Chemistry–A European Journal

Supporting Information

Chemical Bonding in Silicon Carbonyl Complexes

Tetiana Sergeieva, Debdeep Mandal, and Diego M. Andrada*

Contents

Thermodynamics........S3
Silicocene-carbony complex 7'........S4
Natural Bond Orbital (NBO)........S5
Energy Decomposition Analysis (EDA)........S9
Bonding Analysis on the relaxed potential energy surface........S17
xyz coordinate (Å) and Energy (in Hartree)........S24

Thermodynamics

Table S1. Dissociation energy (D_e) and Gibbs Energies (ΔG) for compounds **1-7'**. All values are in kcal/mol.

	**1**		**2**		**3**		**4**		**5**		**6**		**7**		**7'**	
	D_e	ΔG	D_e	ΔG	D_e	ΔG	D_e	ΔG	D_e	ΔG	D_e	ΔG	D_e	ΔG	D_e	ΔG
BP86-D3(BJ)/def2-SVP	38.9	27.3	29.4	16.1	39.3	26.6	51.3	38.0	45.6	34.2	44.8	30.4	1.6	−9.8	9.1	−6.1
BP86-D3(BJ)/def2-TZVPP[a]	38.1	26.5	27.8	14.5	38.5	25.8	50.7	37.4	43.4	32.0	42.9	28.6	0.1	−11.3	3.7	−11.5
WB97XD/def2- SVP	28.7	17.0	18.5	5.5	28.5	15.9	39.8	26.8	40.4	28.0	36.8	23.1	−4.3	−15.7	−4.7	−21.6
B3LYP-D3(BJ)/def2-SVP	31.2	19.5	21.7	8.7	31.3	18.8	43.0	30.0	41.8	30.4	39.1	25.5	−0.3	−11.9	−1.2	−16.0
PBE0-D3(BJ)/def2-SVP	35.2	23.5	25.1	11.9	35.3	22.8	47.4	34.2	44.6	32.4	41.7	27.6	−3.3	−14.3	−5.2	−20.3
M06-2X/def2-SVP	27.0	15.5	16.4	3.6	26.7	14.7	38.9	25.1	41.1	28.2	33.5	29.1	-[b]	-[b]	−14.2	−27.4
M06-2X-D3/def2-SVP	27.0	15.5	16.4	3.7	26.9	14.8	39.1	25.4	41.4	29.0	33.8	21.4	-[b]	-[b]	−16.4	−31.7
MP2/def2-SVP	24.2	12.5	15.0	2.1	27.4	14.8	40.0	27.0	-	-	36.6	-	2.0	−4.6	-[b]	-[b]

[a] Single point on the optimized geometries at BP86-D3(BJ)/def2-SVP.

[b] No stable silylene-carbonyl complex has been found.

Silicocene-carbony complex 7’

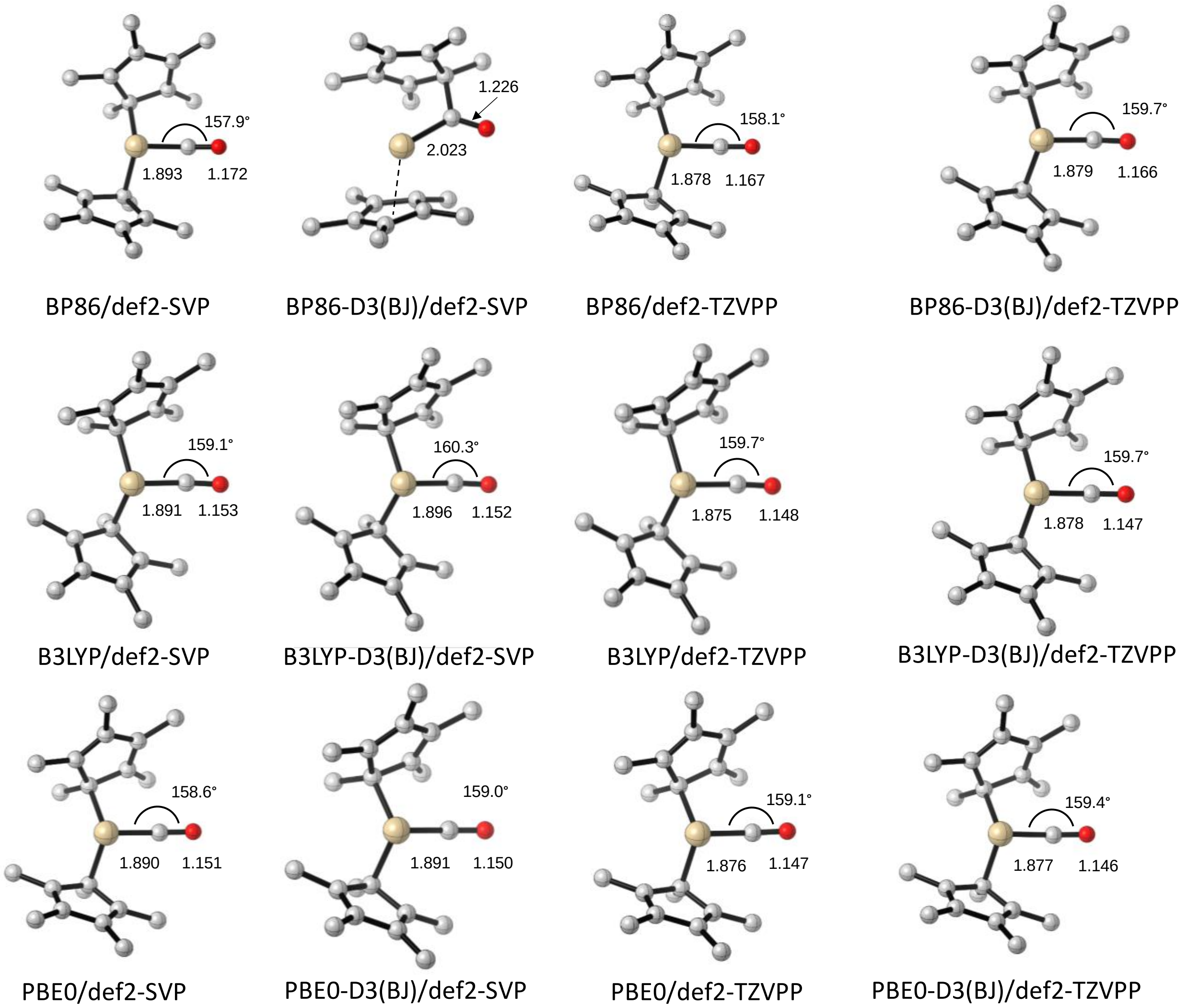

Figure S1. Optimized **7’** compounds at different level of theory with selected bond distances in [Å] and bond angles in [°]. Hydrogen atoms were omitted for clarity.

Natural Bond Orbital (NBO)

Orbital	Occ.	Contribution from atoms to the orb.	Atomic orbitals
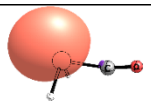	1.71	LP (Si)	*s* (69.76%) *p* (30.10%) *d* (0.13%)
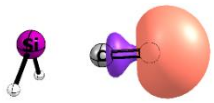	1.98	LP (O)	*s*(54.51%) *p*(45.32%) *d* (0.16%)
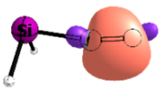	2.00	C (31.06%) –O (68.94%)	C: *s* (33.38%) *p* (66.48%) *d* (0.09%)<br>O: *s*(42.63%) *p* (56.61%) *d* (0.73%)
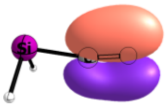	2.00	C (27.23%) –O (72.77%)	C: *s* (1.41%) *p* (98.46%) *d* (0.13%)<br>O: *s*(2.84%) *p* (96.70%) *d* (0.44%)
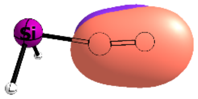	2.00	C (28.33%) –O (71.67%)	C: *s* (0.00%) *p* (99.88%) *d* (0.12%)<br>O: *s*(0.00%) *p* (99.54%) *d* (0.43%)
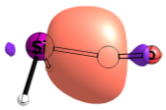	1.98	C (75.58%) –Si (24.42%)	C: *s* (66.72%) *p* (33.23%) *d* (0.03%)<br>Si: *s*(8.63%) *p* (90.23%) *d* (1.12%)
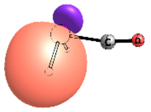	1.88	Si (41.67%) –H (58.33%)	Si: *s* (10.86%) *p* (88.52%) *d* (0.62%)<br>H: *s*(99.57%) *p* (0.41%) *d* (0.02%)
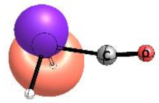	1.88	Si (41.67%) –H (58.33%)	Si: *s* (10.86%) *p* (88.52%) *d* (0.62%)<br>H: *s*(99.57%) *p* (0.41%) *d* (0.02%)
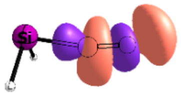	0.04	C (68.94%) –O (31.06%)	C: *s* (33.38%) *p* (66.48%) *d* (0.09%)<br>O: *s*(42.63%) *p* (56.61%) *d* (0.73%)
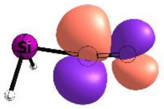	0.34	C (72.77%) –O (27.23%)	C: *s* (1.41%) *p* (98.46%) *d* (0.13%)<br>O: *s*(2.84%) *p* (96.70%) *d* (0.44%)
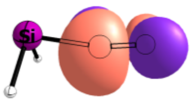	0.10	C (72.77%) –O (27.23%)	C: *s* (0.00%) *p* (99.88%) *d* (0.12%)<br>O: *s*(0.00%) *p* (99.54%) *d* (0.43%)
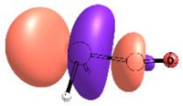	0.01	C (24.42%) –Si (75.58%)	C: *s* (66.72%) *p* (33.23%) *d* (0.03%)<br>Si: *s*(8.63%) *p* (90.23%) *d* (1.12%)

Figure S2. NBO results of **1** at the BP86-D3(BJ)/def2-TZVPP//BP86-D3(BJ)/def2-SVP level of theory using default resonance structure (Structures 1). Hydrogen atoms were omitted for clarity.

Orbital	Occ.	Contribution from atoms to the orb.	Atomic orbitals
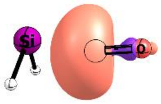	1.51	LP (C)	*s(71.70%)p (28.24%)d (0.03%)*
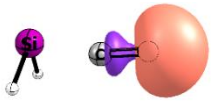	1.98	LP (O)	*s*(54.51%) *p* (45.32%) *d* (0.16%)
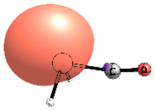	1.71	LP (Si)	*s* (69.76%) *p* (30.10%) *d* (0.13%)
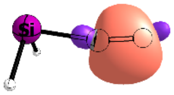	2.00	C (28.53%) –O (71.47%)	C: *s(11.62%)p (88.24%)d (0.12%)*<br>O: *s(18.84%)p (80.58%)d (0.55%)*
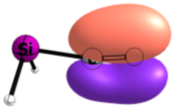	2.00	C (28.33%) –O (71.67%)	C: *s(0.00%)p (99.88%)d (0.12%)*<br>O: *s(0.00%)p (99.54%)d (0.43%)*
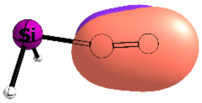	2.00	C (29.93%) –O (70.07%)	C: *s(18.21%)p (81.66%)d (0.10%)*<br>O: *s(26.65%)p (72.70%)d (0.62%)*
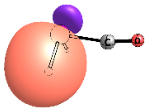	1.88	Si (41.80%) –H (58.20%)	Si: *s(11.86%)p (87.55%)d (0.58%)*<br>H: *s(99.57%)p (0.41%)d (0.02%)*
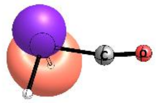	1.88	Si (41.80%) –H (58.20%)	Si: *s(11.86%)p (87.55%)d (0.58%)*<br>H: *s(99.57%)p (0.41%)d (0.02%)*
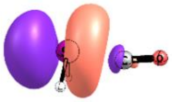	0.49	LV(Si)	*s*(6.63%) *p* (92.15%) *d* (1.19%)
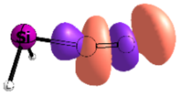	0.21	C (71.47%) –O (28.53%)	C: *s(11.62%)p (88.24%)d (0.12%)*<br>O: *s(18.84%)p (80.58%)d (0.55%)*
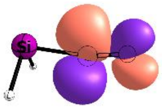	0.10	C (71.67%) –O (28.33%)	C: *s(0.00%)p (99.88%)d (0.12%)*<br>O: *s(0.00%)p (99.54%)d (0.43%)*
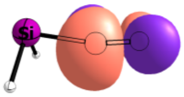	0.18	C (70.07%) –O (29.93%)	C: *s(18.21%)p (81.66%)d (0.10%)*<br>O: *s(26.65%)p (72.70%)d (0.62%)*

Figure S3. NBO results of **1** at the BP86-D3(BJ)/def2-TZVPP//BP86-D3(BJ)/def2-SVP level of theory using chosen resonance structure (Structures 2) Hydrogen atoms were omitted for clarity.

Table S2. Contribution from atoms to the Si-C orbital for compounds **1-7’** calculated at BP86-D3(BJ)/def2-TZVPP//BP86-D3(BJ)/def2-SVP level of theory.

Compound	Contribution from atoms to the orb.	Atomic orbitals
1	Si(24.4%)–C(75.6%)	C: s (66.72%) p (33.23%) d (0.03%)<br>Si: s(8.63%) p (90.23%) d (1.12%)
2	Si(23.6%)–C(76.3%)	C: s(66.50%) p (33.40%) d (0.06%)<br>Si: s(9.26%) p (90.02%) d (0.70%)
3	Si(25.0%)–C(75.0%)	C: s(67.09%) p (32.80%) d (0.06%)<br>Si: s(9.77%) p (89.32%) d (0.90%)
4	Si(26.1%)–C(73.9%)	C: s(67.50%) p (32.37%) d (0.08%)<br>Si: s(10.51%) p (88.42%) d (1.05%)
5	Si(24.9%)–C(75.1%)	C: s(69.33%) p (30.53%) d (0.10%)<br>Si: s(9.82%) p (88.82%) d (1.34%)
6	Si(26.1%)–C(73.4%)	C: s(65.02%) p (34.88%) d (0.07%)<br>Si: s(15.48%) p (83.37%) d (1.13%)
7	Si(21.1%)–C(78.9%)	C: s(67.83%) p (32.08%) d (0.07%)<br>Si: s(6.35%) p (92.75%) d (0.88%)
7’	Si(22.3%)–C(77.6%)	C: s(67.87%) p (32.00%) d (0.10%)<br>Si: s(9.01%) p (90.18%) d (0.80%)

Table S3. NBO results for two selected Lewis structures at the BP86-D3(BJ)/def2-TZVPP//BP86-D3(BJ)/def2-SVP level of theory.

	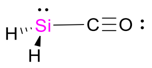	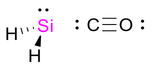
Core	13.99992 (99.999% of 14)	13.99992 (99.999% of 14)
Valence Lewis	15.43078 (96.442% of 16)	14.95084 (93.443% of 16)
Total Lewis	29.43070 (98.102% of 30)	28.95075 (96.503% of 30)
Valence non-Lewis	0.51813 (1.727% of 30)	0.99816 (3.327% of 30)
Rydberg non-Lewis	0.05117 (0.171% of 30)	0.05109 (0.170% of 30)
Total non-Lewis	0.56930 (1.898% of 30)	1.04925 (3.497% of 30)

Table S4. NBO Two-electron interactions and associated second-order perturbational energies (ΔE(2) in kcal mol^{-1}) at the BP86-D3(BJ)/def2-TZVPP//BP86-D3(BJ)/def2-SVP level of theory for **1** using two different Lewis structures.

	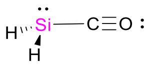	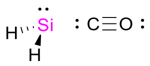
$\sigma_{CO} \rightarrow p_{Si}$	---	265.5
$sp^2 \rightarrow \pi^*_{CO}$	5.2	10.7
$\sigma_{RSi}^{\perp} \rightarrow \pi^*_{CO}$	19.5	11.8

Energy Decomposition Analysis (EDA)

Deformation Desities	R_2Si orbitals	CO Orbitals
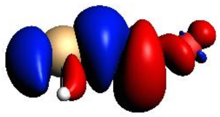 $\Delta E_{orb(1)} = -50.5$ kcal/mol<br>$\lvert \nu_2 \rvert = 0.56$	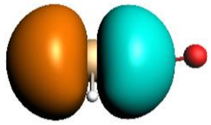 LUMO = −4.12 eV<br>$\nu_1 = 0.36$	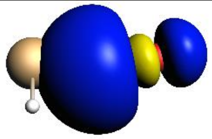 HOMO = −9.20 eV<br>$\nu_1 = -0.41$
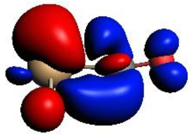 $\Delta E_{orb(2)} = -36.6$ kcal/mol<br>$\lvert \nu_2 \rvert = 0.71$	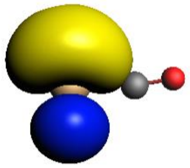 HOMO = −5.75 eV<br>$\nu_2 = -0.42$	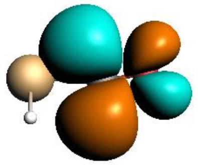 LUMO = −2.32 eV<br>$\nu_2 = 0.37$
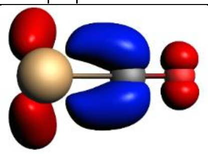 $\Delta E_{orb(3)} = -10.0$ kcal/mol<br>$\lvert \nu_3 \rvert = 0.32$	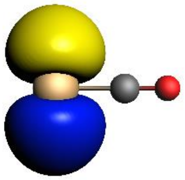 HOMO-1 = −8.31 eV<br>$\nu_3 = -0.10$	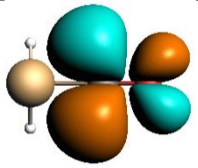 LUMO = −2.32 eV<br>$\nu_3 = 0.12$

Figure S4. Plot of deformation densities $\Delta\rho_{1\text{-}3}$ (isovalue = 0.003) of the pairwise orbital interaction and shape of the most important occupied and vacant orbitals (isovalue = 0.03) in silylene carbonyl complexes **1** with the orbital interaction energies ΔE_{orb} (in kcal/mol) and their eigenvalues ν (in e). The direction of the charge flow is red→blue. The eigenvalues ν indicate the amount of donated (negative numbers) and accepted charge (positive numbers). The occupied orbitals are shown in yellow and blue for the different phases, while the unoccupied orbitals are in cyan and orange.

Deformation Desities	R_2Si orbitals	CO Orbitals
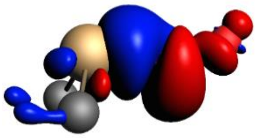<br>$\Delta E_{orb(1)} = -39.9$ kcal/mol<br>$\lvert\nu_1\rvert = 0.51$	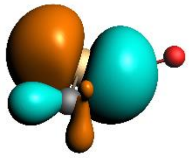<br>LUMO = −3.12 eV<br>$\nu_1 = 0.23$	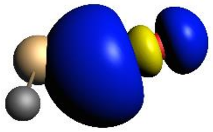<br>HOMO = −9.22 eV<br>$\nu_1 = -0.29$
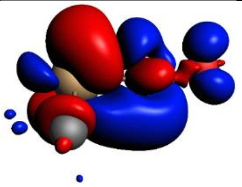<br>$\Delta E_{orb(2)} = -70.3$ kcal/mol<br>$\lvert\nu_2\rvert = 0.93$	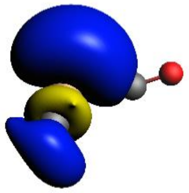<br>HOMO = −4.85 eV<br>$\nu_2 = -0.58$	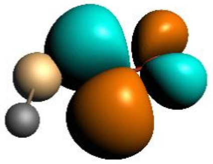<br>LUMO = −2.40 eV<br>$\nu_2 = 0.39$
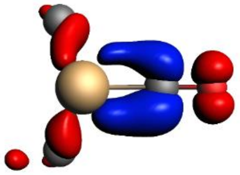<br>$\Delta E_{orb(3)} = -7.8$ kcal/mol<br>$\lvert\nu_3\rvert = 0.28$	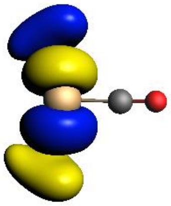<br>HOMO-1 = −7.51 eV<br>$\nu_3 = -0.05$	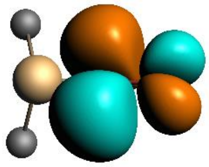<br>LUMO = −2.40 eV<br>$\nu_3 = 0.06$

Figure S5. Plot of deformation densities $\Delta\rho_{1\text{-}3}$ (isovalue = 0.003) of the pairwise orbital interaction and shape of the most important occupied and vacant orbitals (isovalue = 0.03) in silylene carbonyl complexes **2** with the orbital interaction energies ΔE_{orb} (in kcal/mol) and their eigenvalues ν (in e). The direction of the charge flow is red→blue. The eigenvalues ν indicate the amount of donated (negative numbers) and accepted charge (positive numbers). The occupied orbitals are shown in yellow and blue for the different phases, while the unoccupied orbitals are in cyan and orange.

Deformation Desities	R_2Si orbitals	CO Orbitals
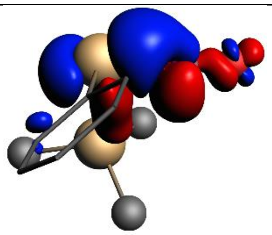<br>$\Delta E_{orb(1)}$ = −47.4 kcal/mol<br>$\lvert\nu_1\rvert$ = 0.62	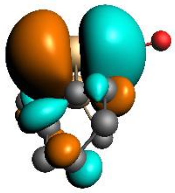<br>LUMO = −3.69 eV<br>ν_1 = 0.32	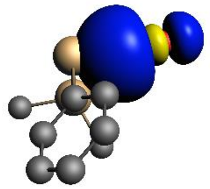<br>HOMO = −9.22 eV<br>ν_1 = −0.32
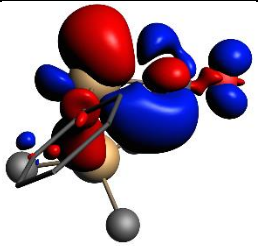<br>$\Delta E_{orb(2)}$ = −54.5 kcal/mol<br>$\lvert\nu_2\rvert$ = 0.85	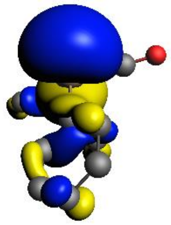<br>HOMO = −4.66 eV<br>ν_2 = −0.47	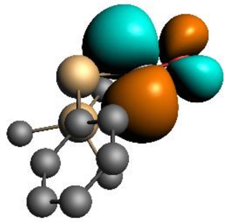<br>LUMO = −2.41 eV<br>ν_2 = 0.27
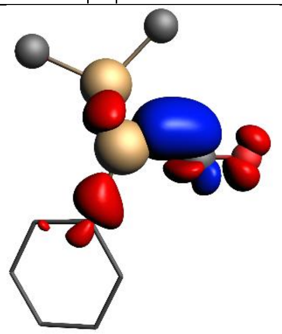<br>$\Delta E_{orb(3)}$ = −16.1 kcal/mol<br>$\lvert\nu_3\rvert$ = 0.40	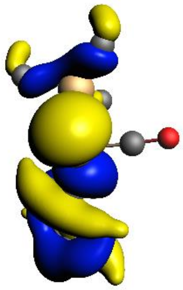<br>HOMO-1 = −6.58 eV<br>ν_3 = −0.07	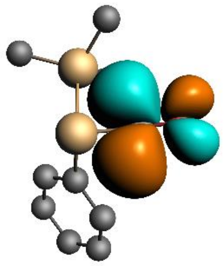<br>LUMO = −2.41 eV<br>ν_3 = 0.07

Figure S6. Plot of deformation densities $\Delta\rho_{1\text{-}3}$ (isovalue = 0.003) of the pairwise orbital interaction and shape of the most important occupied and vacant orbitals (isovalue = 0.03) in silylene carbonyl complexes **3** with the orbital interaction energies ΔE_{orb} (in kcal/mol) and their eigenvalues *ν* (in e). The direction of the charge flow is red→blue. The eigenvalues *ν* indicate the amount of donated (negative numbers) and accepted charge (positive numbers). The occupied orbitals are shown in yellow and blue for the different phases, while the unoccupied orbitals are in cyan and orange.

Deformation Desities	R_2Si orbitals	CO Orbitals
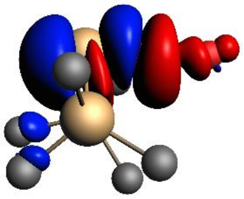 $\Delta E_{orb(1)} = -60.8$ kcal/mol $\lvert\nu_1\rvert = 0.66$	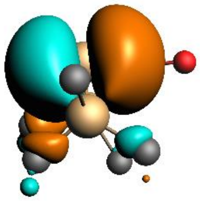 LUMO = −3.94 eV $\nu_1 = 0.51$	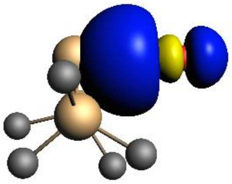 HOMO = −9.23 eV $\nu_1 = -0.47$
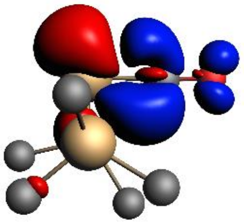 $\Delta E_{orb(2)} = -38.0$ kcal/mol $\lvert\nu_2\rvert = 0.74$	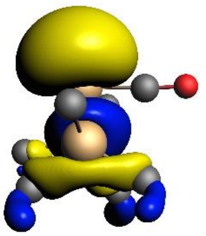 HOMO = −4.78 eV $\nu_2 = -0.42$	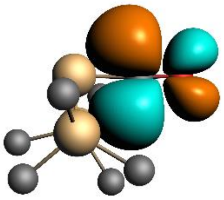 LUMO = −2.45 eV $\nu_2 = 0.41$
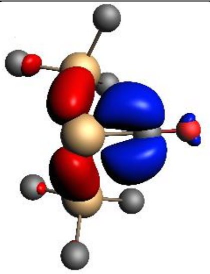 $\Delta E_{orb(3)} = -23.9$ kcal/mol $\lvert\nu_3\rvert = 0.55$	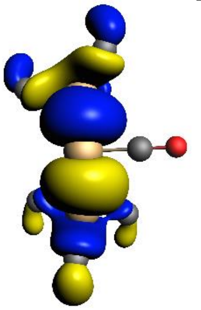 HOMO-1 = −5.75 eV $\nu_3 = -0.24$	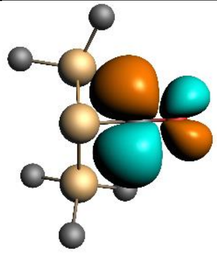 LUMO = −2.45 eV $\nu_3 = 0.26$

Figure S7. Plot of deformation densities $\Delta\rho_{1\text{-}3}$ (isovalue = 0.003) of the pairwise orbital interaction and shape of the most important occupied and vacant orbitals (isovalue = 0.03) in silylene carbonyl complexes **4** with the orbital interaction energies ΔE_{orb} (in kcal/mol) and their eigenvalues ν (in e). The direction of the charge flow is red→blue. The eigenvalues ν indicate the amount of donated (negative numbers) and accepted charge (positive numbers). The occupied orbitals are shown in yellow and blue for the different phases, while the unoccupied orbitals are in cyan and orange.

Deformation Desities	R_2Si orbitals	CO Orbitals
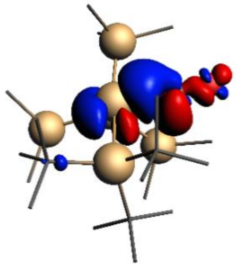<br>$\Delta E_{orb(1)} = -50.5$ kcal/mol<br>$\lvert\nu_1\rvert = 0.64$	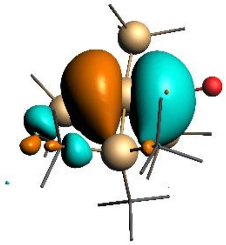<br>LUMO = −3.75 eV<br>$\nu_1 = 0.35$	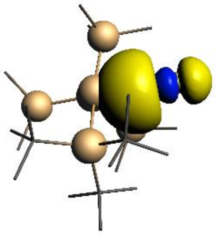<br>HOMO = −9.24 eV<br>$\nu_1 = -0.35$
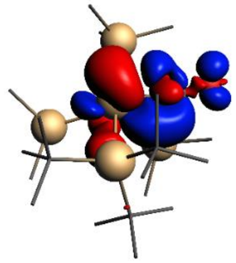<br>$\Delta E_{orb(2)} = -63.5$ kcal/mol<br>$\lvert\nu_2\rvert = 0.90$	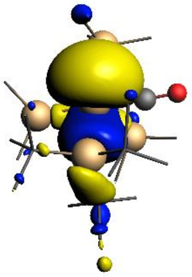<br>HOMO = −4.25 eV<br>$\nu_2 = -0.50$	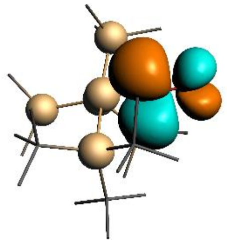<br>LUMO = −2.48 eV<br>$\nu_2 = 0.42$
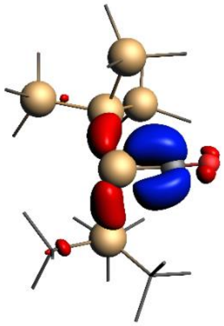<br>$\Delta E_{orb(3)} = -18.9$ kcal/mol<br>$\lvert\nu_3\rvert = 0.48$	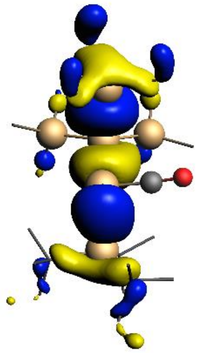<br>HOMO-1 = −5.71 eV<br>$\nu_3 = -0.09$	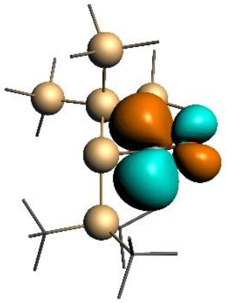<br>LUMO = −2.48 eV<br>$\nu_3 = 0.20$

Figure S8. Plot of deformation densities $\Delta\rho_{1\text{-}3}$ (isovalue = 0.003) of the pairwise orbital interaction and shape of the most important occupied and vacant orbitals (isovalue = 0.03) in silylene carbonyl complexes **5** with the orbital interaction energies ΔE_{orb} (in kcal/mol) and their eigenvalues ν (in e). The direction of the charge flow is red→blue. The eigenvalues ν indicate the amount of donated (negative numbers) and accepted charge (positive numbers). The occupied orbitals are shown in yellow and blue for the different phases, while the unoccupied orbitals are in cyan and orange.

Deformation Desities	R_2Si orbitals	CO Orbitals
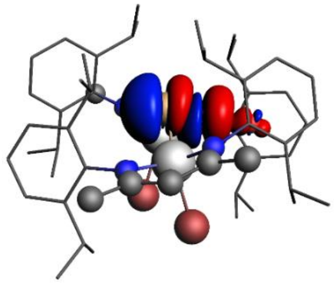<br>$\Delta E_{orb(1)} = -63.4$ kcal/mol<br>$\lvert\nu_1\rvert = 0.69$	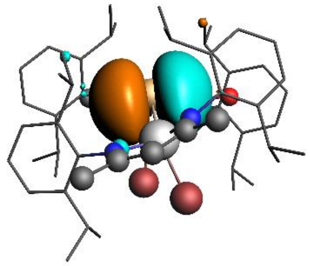<br>LUMO = −4.15 eV<br>$\nu_1 = 0.53$	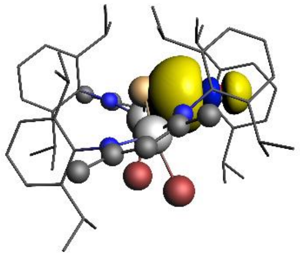<br>HOMO = −9.22 eV<br>$\nu_1 = -0.47$
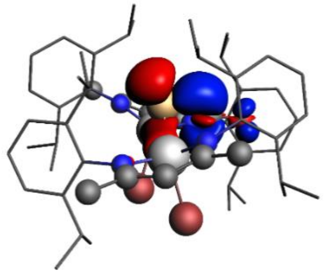<br>$\Delta E_{orb(2)} = -39.8$ kcal/mol<br>$\lvert\nu_2\rvert = 0.74$	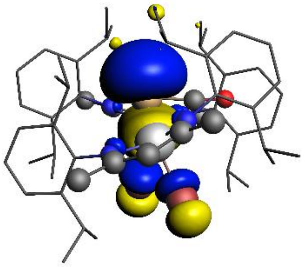<br>HOMO = −4.70 eV<br>$\nu_2 = -0.38$	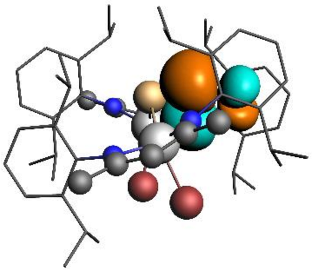<br>LUMO = −2.43 eV<br>$\nu_2 = 0.27$
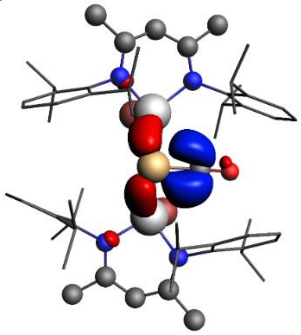<br>$\Delta E_{orb(3)} = -23.7$ kcal/mol<br>$\lvert\nu_3\rvert = 0.53$	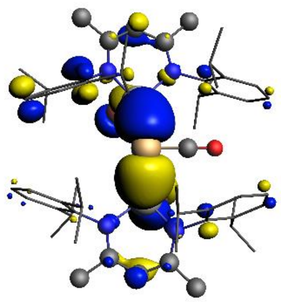<br>HOMO-10 = −6.41 eV<br>$\nu_3 = -0.1$	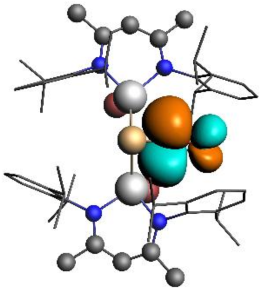<br>LUMO = −2.43 eV<br>$\nu_3 = 0.18$

Figure S9. Plot of deformation densities $\Delta\rho_{1\text{-}3}$ (isovalue = 0.003) of the pairwise orbital interaction and shape of the most important occupied and vacant orbitals (isovalue = 0.03) in silylene carbonyl complexes **6** with the orbital interaction energies ΔE_{orb} (in kcal/mol) and their eigenvalues ν (in e). The direction of the charge flow is red→blue. The eigenvalues ν indicate the amount of donated (negative numbers) and accepted charge (positive numbers). The occupied orbitals are shown in yellow and blue for the different phases, while the unoccupied orbitals are in cyan and orange.

Deformation Desities	R_2Si orbitals	CO Orbitals
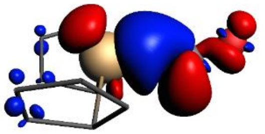<br>$\Delta E_{orb(1)} = -35.3$ kcal/mol<br>$\lvert \nu_1 \rvert = 0.53$	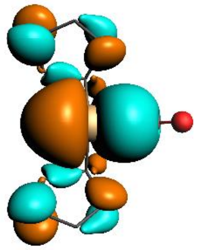<br>LUMO = −3.02 eV<br>$\nu_1 = 0.20$	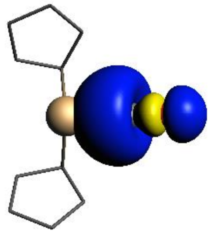<br>HOMO = −9.21 eV<br>$\nu_1 = -0.23$
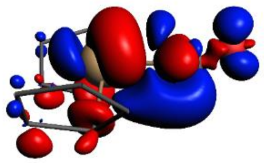<br>$\Delta E_{orb(2)} = -48.5$ kcal/mol<br>$\lvert \nu_2 \rvert = 0.77$	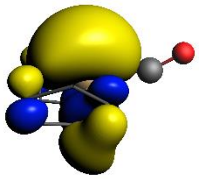<br>HOMO = −5.43 eV<br>$\nu_2 = -0.25$	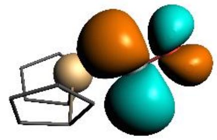<br>LUMO = −2.36 eV<br>$\nu_2 = 0.33$
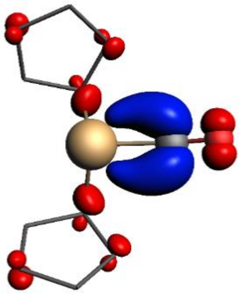<br>$\Delta E_{orb(3)} = -8.8$ kcal/mol<br>$\lvert \nu_3 \rvert = 0.37$	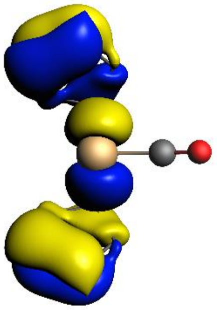<br>HOMO-2 = −6.40 eV<br>$\nu_3 = -0.05$	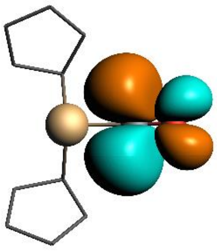<br>LUMO = −2.36 eV<br>$\nu_3 = 0.12$

Figure S10. Plot of deformation densities $\Delta\rho_{1\text{-}3}$ (isovalue = 0.003) of the pairwise orbital interaction and shape of the most important occupied and vacant orbitals (isovalue = 0.03) in silylene carbonyl complexes **7** with the orbital interaction energies ΔE_{orb} (in kcal/mol) and their eigenvalues ν (in e). The direction of the charge flow is red→blue. The eigenvalues ν indicate the amount of donated (negative numbers) and accepted charge (positive numbers). The occupied orbitals are shown in yellow and blue for the different phases, while the unoccupied orbitals are in cyan and orange.

Deformation Desities	R_2Si orbitals	CO Orbitals
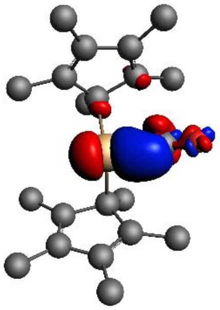 $\Delta E_{orb(1)} = -33.0$ kcal/mol<br>$\|\nu_1\| = 0.58$	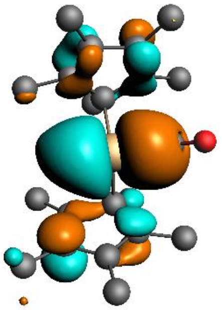 LUMO = −2.50 eV<br>$\nu_1 = 0.11$	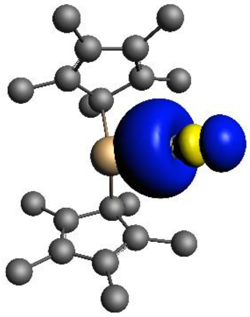 HOMO = −9.24 eV<br>$\nu_1 = -0.13$
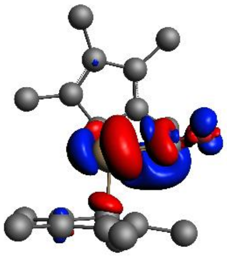 $\Delta E_{orb(2)} = -74.4$ kcal/mol<br>$\|\nu_2\| = 0.96$	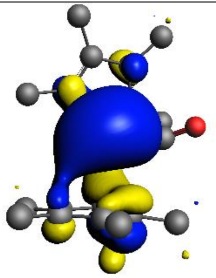 HOMO = −4.50 eV<br>$\nu_2 = -0.38$	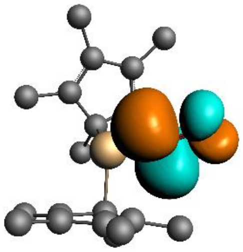 LUMO = −2.45 eV<br>$\nu_2 = 0.35$
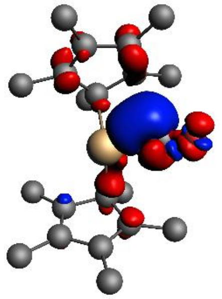 $\Delta E_{orb(3)} = -17.2$ kcal/mol<br>$\|\nu_3\| = 0.43$	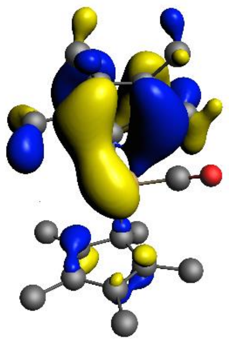 HOMO-1 = −4.76 eV<br>$\nu_3 = -0.04$	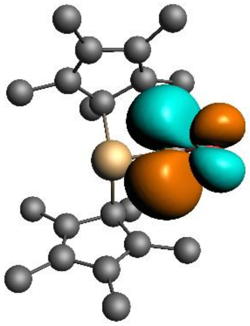 LUMO = −2.50 eV<br>$\nu_3 = 0.10$

Figure S11. Plot of deformation densities $\Delta\rho_{1\text{-}3}$ (isovalue = 0.003) of the pairwise orbital interaction and shape of the most important occupied and vacant orbitals (isovalue = 0.03) in silylene carbonyl complexes **7’** with the orbital interaction energies ΔE_{orb} (in kcal/mol) and their eigenvalues *ν* (in e). The direction of the charge flow is red→blue. The eigenvalues *ν* indicate the amount of donated (negative numbers) and accepted charge (positive numbers). The occupied orbitals are shown in yellow and blue for the different phases, while the unoccupied orbitals are in cyan and orange.

Bonding Analysis on the relaxed potential energy surface

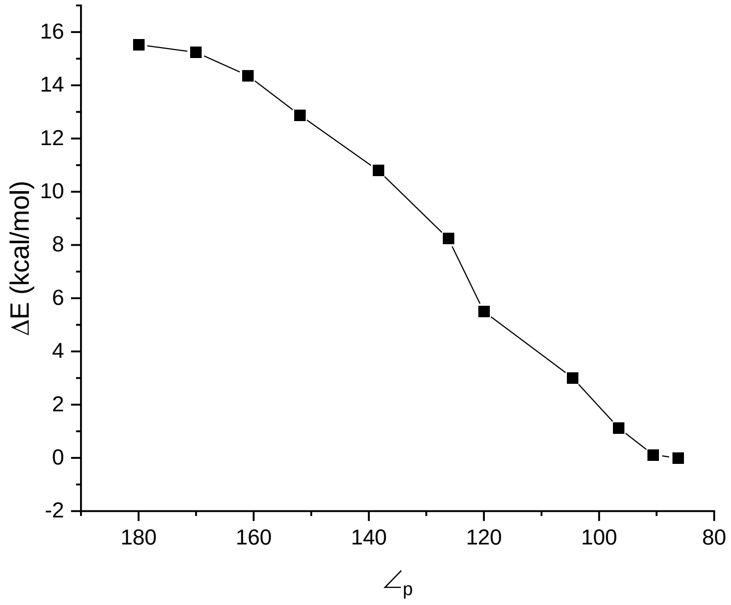

Figure S12. Potential Energy Surface of **1** on the pyramidalization angle at the BP86-D3(BJ)/def2-SVP.

Table S5. EDA-NOCV results of the Si-CO bond in SiH_2CO (**1**) at BP86-D3(BJ)/TZ2P//BP86-D3(BJ)/def2-SVP using SiH_2 ($a_1^8\,a_2^0\,b_1^4\,b_2^2$) and CO ($a_1^6\,a_2^0\,b_1^2\,b_2^2$) fragments. All values are in kcal/mol. The smallest ΔE_{orb} values for each species are indicated in bold.

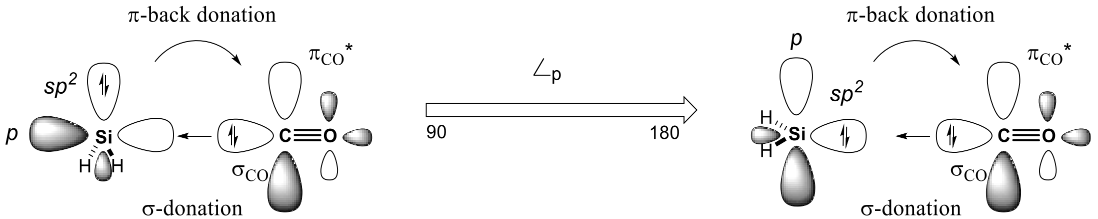

	**90**	**100**	**110**	**120**	**130**	**140**	**150**	**160**	**170**	**180**
ΔE_{int}	-39.6	-41.1	-42.7	-44.2	-45.1	-45.2	-44.7	-43.9	-45.6	-43.0
ΔE_{Pauli}	146.5	160.3	186.1	228.2	289.4	361.8	432.2	487.1	520.1	531.2
ΔE_{disp}[a]	-1.9 (1.0%)	-1.9 (1.0%)	-1.9 (0.8%)	-1.9 (0.7%)	-1.9 (0.6%)	-1.9 (0.5%)	-1.9 (0.4%)	-1.9 (0.4%)	-1.9 (0.3%)	-1.9 (0.3%)
ΔE_{elstat}[a]	-79.8 (42.9%)	-87.1 (43.3%)	-100.2 (43.8%)	-120.4 (44.2%)	-147.3 (44.0%)	-175.4 (43.1%)	-199.4 (41.8%)	-215.9 (40.7%)	-225.0 (39.8%)	-227.8 (39.7%)
ΔE_{orb}[a]	**-104.3** (56.1%)	**-112.3** (55.8%)	**-126.7** (55.4%)	**-150.1** (55.1%)	**-185.4** (55.4%)	-229.7 (56.4%)	-275.6 (57.8%)	-313.3 (59.0%)	-338.8 (59.9%)	-344.6 (60.0%)
ΔE_{orb1} ($\sigma_{CO} \rightarrow p_{Si}$)[b]	-44.7 (42.9%)	-42.8 (38.1%)	-40.7 (32.1%)	-38.6 (25.7%)	-36.6 (19.8%)	-34.7 (15.1%)	-32.9 (11.9%)	-31.5 (10.1%)	-30.4 (9.0%)	-30.3 (8.8%)
ΔE_{orb2} ($sp^2 \rightarrow \pi^*_{CO}$)[b]	-45.3 (43.5%)	-56.4 (50.2%)	-73.9 (58.3%)	-100.4 (66.9%)	-138.7 (74.8%)	-185.6 (80.8%)	-233.9 (84.9%)	-273.4 (87.3%)	-299.5 (88.4%)	-306.2 (88.9%)
ΔE_{orb3} ($\sigma_{RSi\perp} \rightarrow \pi^*_{CO}$)[b]	-9.5 (9.2%)	-9.0 (8.0%)	-8.6 (6.8%)	-8.1 (5.4%)	-7.6 (4.1%)	-7.1 (3.1%)	-6.7 (2.4%)	-6.4 (2.0%)	-6.2 (1.8%)	-6.1 (1.8%)
$\Delta E_{orb\ rest}$[b]	-4.7 (4.5%)	-4.1 (3.7%)	-3.5 (2.8%)	-3.0 (2.0%)	-2.5 (1.4%)	-2.2 (1.0%)	-2.0 (0.7%)	-2.0 (0.6%)	-2.6 (0.8%)	-2.0 (0.6%)

[a]The values in parentheses give the percentage contribution to the total attractive interactions $\Delta E_{elstat} + \Delta E_{orb}$.
[b]The values in parentheses give the percentage contribution to the total orbital interactions ΔE_{orb}.

Table S6. EDA-NOCV results of the Si-CO bond in SiH_2CO (**1**) at BP86-D3(BJ)/TZ2P//BP86/def2-SVP using SiH_2 ($a_1^6\,a_2^0\,b_1^4\,b_2^4$) and CO ($a_1^6\,a_2^0\,b_1^2\,b_2^2$) fragments. All values are in kcal/mol. The smallest ΔE_{orb} values for each species are indicated in bold.

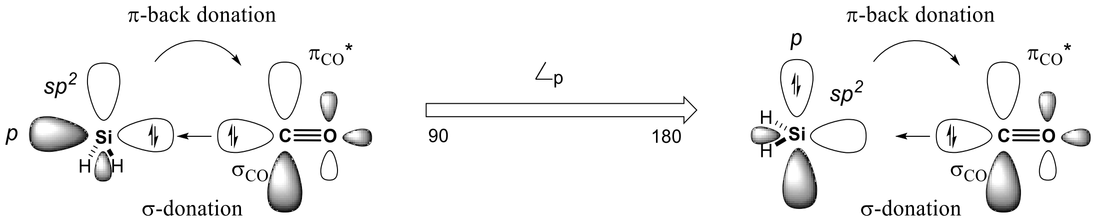

	**90**	**100**	**110**	**120**	**130**	**140**	**150**	**160**	**170**	**180**
ΔE_{int}	-140.1	-133.1	-125.9	-119.3	-113.7	-109.4	-106.6	-104.3	-106.1	-103.5
ΔE_{Pauli}	405.8	401.3	387.8	358.1	308.2	249.4	196.0	156.5	134.1	129.0
ΔE_{disp}[a]	-1.9 (0.4%)	-1.9 (0.4%)	-1.9 (0.4%)	-1.9 (0.4%)	-1.9 (0.4%)	-1.9 (0.5%)	-1.9 (0.6%)	-1.9 (0.7%)	-1.9 (0.8%)	-1.9 (0.8%)
ΔE_{elstat}[a]	-183.8 (33.7%)	-183.2 (34.3%)	-180.4 (35.1%)	-172.7 (36.2%)	-157.1 (37.2%)	-135.4 (37.7%)	-112.5	-94.2 (36.1%)	-83.6 (34.8%)	-79.9 (34.3%)
ΔE_{orb}[a]	-360.3 (66.0%)	-349.2 (65.4%)	-331.4 (64.5%)	-302.8 (63.4%)	-262.9 (62.3%)	-221.7 (61.8%)	**-188.2** (62.2%)	**-164.8** (63.2%)	**-154.6** (64.4%)	**-150.8** (64.8%)
ΔE_{orb1} ($\sigma_{CO} \rightarrow p_{Si}$)[b]	-25.3 (7.0%)	-25.3 (7.2%)	-25.6 (7.7%)	-26.4 (8.7%)	-28.4 (10.8%)	-32.7 (14.8%)	-40.2 (21.4%)	-50.2 (30.5%)	-73.3 (47.4%)	-69.4 (46.0%)
ΔE_{orb2} ($sp^2 \rightarrow \pi^*_{CO}$)[b]	-323.9 (89.9%)	-313.1 (89.6%)	-294.8 (88.9%)	-264.7 (87.4%)	-221.8 (84.4%)	-175.1 (79.0%)	-133.4 (70.9%)	-99.8 (60.5%)	-65.6 (42.4%)	-66.2 (43.9%)
ΔE_{orb3} ($\sigma_{RSi\perp} \rightarrow \pi^*_{CO}$)[b]	-8.9 (2.5%)	-8.6 (2.5%)	-8.4 (2.5%)	-8.3 (2.7%)	-8.4 (3.2%)	-8.5 (3.8%)	-8.6 (4.6%)	-8.6 (5.2%)	-8.8 (5.7%)	-8.8 (5.9%)
$\Delta E_{orb\ rest}$[b]	-2.1 (0.6%)	-2.3 (0.7%)	-2.7 (0.8%)	-3.4 (1.1%)	-4.3 (1.7%)	-5.3 (2.4%)	-6.0 (3.2%)	-6.2 (3.8%)	-6.9 (4.5%)	-6.4 (4.3%)

[a]The values in parentheses give the percentage contribution to the total attractive interactions $\Delta E_{elstat} + \Delta E_{orb}$.
[b]The values in parentheses give the percentage contribution to the total orbital interactions ΔE_{orb}.

Table S7. EDA-NOCV results of the Si-CO bond in SiH_2CO (**1**) at BP86-D3(BJ)/TZ2P//BP86/def2-SVP using SiH_2 ($a_1^7\,a_2^0\,b_1^4\,b_2^3$) and CO ($a_1^5\,a_2^0\,b_1^2\,b_2^3$) fragments. All values are in kcal/mol. The smallest ΔE_{orb} values for each species are indicated in bold.

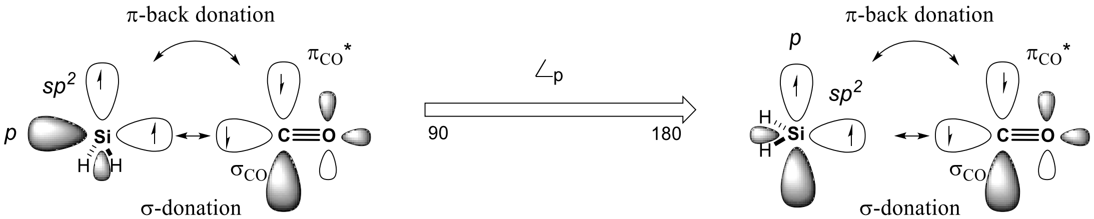

	**90**	**100**	**110**	**120**	**130**	**140**	**150**	**160**	**170**	**180**
ΔE_{int}	-196.9	-193.7	-190.4	-187.2	-184.3	-181.7	-179.6	-178.6	-177.6	-176.9
ΔE_{Pauli}	111.0	113.4	116.6	119.7	122.1	123.6	124.3	122.7	124.5	124.6
ΔE_{disp}	-1.9	-1.9	-1.9	-1.9	-1.9	-1.9	-1.9	-1.9	-1.9	-1.9
	(0.6%)	(0.6%)	(0.6%)	(0.6%)	(0.6%)	(0.6%)	(0.6%)	(0.6%)	(0.6%)	(0.6%)
ΔE_{elstat}[a]	-89.9	-92.9	-96.2	-99.1	-100.4	-99.9	-98.2	-95.9	-94.8	-94.8
	(29.2%)	(30.2%)	(31.4%)	(32.3%)	(32.8%)	(32.7%)	(32.3%)	(31.8%)	(31.4%)	(31.4%)
ΔE_{orb}[a]	-216.0	-212.3	-208.8	-205.9	-204.1	**-203.5**	-203.8	-203.5	-205.4	-204.8
	(70.2%)	(69.1%)	(68.0%)	(67.1%)	(66.6%)	(66.7%)	(67.1%)	(67.6%)	(68.0%)	(67.9%)
ΔE_{orb1} (σ_{CO} ←→ p_{Si})[b]	-146.6	-145.1	-143.3	-141.5	-140.3	-140.6	-142.0	-143.4	-144.6	-145.4
	(67.8%)	(68.3%)	(68.6%)	(68.7%)	(68.8%)	(69.1%)	(69.7%)	(70.4%)	(70.4%)	(71.0%)
ΔE_{orb2} (sp^2←→ π^*_{CO})[b]	-56.4	-54.9	-53.7	-52.8	-52.0	-50.9	-49.4	-47.8	-47.4	-46.5
	(26.1%)	(25.9%)	(25.7%)	(25.6%)	(25.5%)	(25.0%)	(24.2%)	(23.5%)	(23.1%)	(22.7%)
ΔE_{orb3} ($\sigma_{RSi\perp}$→ π^*_{CO})[b]	-7.5	-6.8	-6.4	-6.2	-6.1	-6.3	-6.5	-6.5	-6.8	-6.9
	(3.5%)	(3.2%)	(3.1%)	(3.0%)	(3.0%)	(3.1%)	(3.2%)	(3.2%)	(3.3%)	(3.4%)
$\Delta E_{orb\ rest}$[b]	-5.5	-5.4	-5.4	-5.5	-5.6	-5.8	-5.9	-5.9	-6.5	-6.0
	(2.6%)	(2.6%)	(2.6%)	(2.7%)	(2.8%)	(2.8%)	(2.9%)	(2.9%)	(3.2%)	(2.9%)

[a]The values in parentheses give the percentage contribution to the total attractive interactions ΔE_{elstat} + ΔE_{orb}.
[b]The values in parentheses give the percentage contribution to the total orbital interactions ΔE_{orb}.

$\Delta\rho_{\text{orb(1)}}$	$\Delta\rho_{\text{orb(2)}}$	$\Delta\rho_{\text{orb(3)}}$
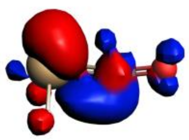<br>$\Delta E_{\text{orb(1)}} = -45.3$ kcal/mol<br>$\lvert\nu_1\rvert = 0.76$	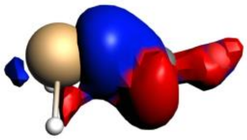<br>$\Delta E_{\text{orb(2)}} = -44.7$ kcal/mol<br>$\lvert\nu_2\rvert = 0.54$	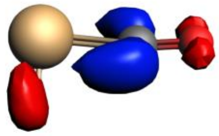<br>$\Delta E_{\text{orb(3)}} = -9.5$ kcal/mol<br>$\lvert\nu_3\rvert = 0.31$

Figure S12. Plot of deformation densities $\Delta\rho_{1\text{-}3}$ (isovalue = 0.003) of the pairwise orbital interaction and the orbital interaction energies ΔE_{orb} (in kcal/mol) and their eigenvalues *v* (in e) of **1** at pyramidalization angle ($\angle_p$) 90 °. The fragmentation is SiH_2 ($a_1^8\, a_2^0\, b_1^4\, b_2^2$) and CO ($a_1^6\, a_2^0\, b_1^2\, b_2^2$), see Table S5. The direction of the charge flow is red→blue. The eigenvalues *v* indicate the amount of donated (negative numbers) and accepted charge (positive numbers).

$\Delta\rho_{\text{orb(1)}}$	$\Delta\rho_{\text{orb(2)}}$	$\Delta\rho_{\text{orb(3)}}$
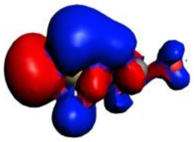<br>$\Delta E_{\text{orb(1)}} = −\text{-}323.9$ kcal/mol<br>$\lvert\nu_1\rvert = 0.58$	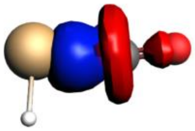<br>$\Delta E_{\text{orb(2)}} = -25.3$ kcal/mol<br>$\lvert\nu_2\rvert = 0.32$	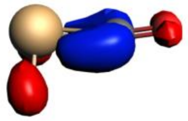<br>$\Delta E_{\text{orb(3)}} = -9.0$ kcal/mol<br>$\lvert\nu_3\rvert = 0.32$

Figure S13. Plot of deformation densities $\Delta\rho_{1\text{-}3}$ (isovalue = 0.003) of the pairwise orbital interaction and the orbital interaction energies ΔE_{orb} (in kcal/mol) and their eigenvalues *v* (in e) of **1** at pyramidalization angle ($\angle_p$) 90 °. The fragmentation is SiH_2 ($a_1^6\, a_2^0\, b_1^4\, b_2^4$) and CO ($a_1^6\, a_2^0\, b_1^2\, b_2^2$), see Table S6. The direction of the charge flow is red→blue. The eigenvalues *v* indicate the amount of donated (negative numbers) and accepted charge (positive numbers).

$\Delta\rho_{\text{orb(1)}}$	$\Delta\rho_{\text{orb(2)}}$	$\Delta\rho_{\text{orb(3)}}$
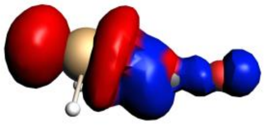<br>$\Delta E_{\text{orb(1)}\alpha\beta} = -146.6$ kcal/mol<br>$\lvert\nu_1\rvert_\alpha = 0.80$; $\lvert\nu_1\rvert_\beta = 0.36$	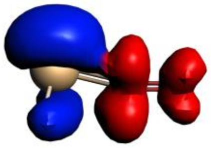<br>$\Delta E_{\text{orb(2)}\alpha\beta} = -56.4$ kcal/mol<br>$\lvert\nu_2\rvert_\alpha = 0.26$; $\lvert\nu_2\rvert_\beta = 0.70$	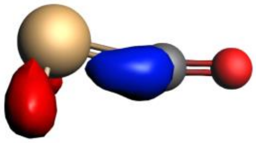<br>$\Delta E_{\text{orb(3)}\alpha\beta} = -7.5$ kcal/mol<br>$\lvert\nu_3\rvert_\alpha = 0.15$; $\lvert\nu_3\rvert_\beta = 0.15$

Figure S14. Plot of deformation densities $\Delta\rho_{1\text{-}3}$ (isovalue = 0.003) of the pairwise orbital interaction and the orbital interaction energies ΔE_{orb} (in kcal/mol) and their eigenvalues *v* (in e) of **1** at pyramidalization angle ($\angle_p$) 90 °. The fragmentation is SiH_2 ($a_1^7\, a_2^0\, b_1^4\, b_2^3$) and CO ($a_1^5\, a_2^0\, b_1^2\, b_2^3$), see Table S7. The direction of the charge flow is red→blue. The eigenvalues *v* indicate the amount of donated (negative numbers) and accepted charge (positive numbers).

$\Delta\rho$ orb(1)	$\Delta\rho$ orb(2)	$\Delta\rho$ orb(3)
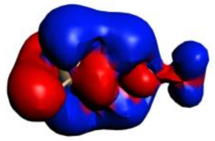	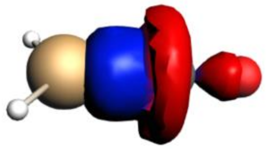	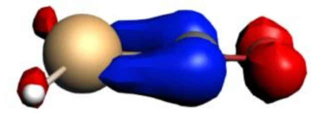
$\Delta E_{orb(1)}$ = −306.2 kcal/mol<br>$\lvert\nu_1\rvert$ = 2.00	$\Delta E_{orb(2)}$ = −30.3 kcal/mol<br>$\lvert\nu_2\rvert$ = 0.37	$\Delta E_{orb(3)}$ = −6.1 kcal/mol<br>$\lvert\nu_3\rvert$ = 0.24

Figure S15. Plot of deformation densities $\Delta\rho_{1\text{-}3}$ (isovalue = 0.003) of the pairwise orbital interaction and the orbital interaction energies ΔE_{orb} (in kcal/mol) and their eigenvalues ν (in e) of **1** at pyramidalization angle ($\angle_p$) 180 °. The fragmentation is SiH_2 (a_1^8 a_2^0 b_1^4 b_2^2) and CO (a_1^6 a_2^0 b_1^2 b_2^2), see Table S5. The direction of the charge flow is red→blue. The eigenvalues ν indicate the amount of donated (negative numbers) and accepted charge (positive numbers).

$\Delta\rho$ orb(1)	$\Delta\rho$ orb(2)	$\Delta\rho$ orb(3)
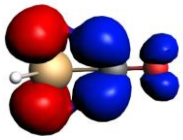	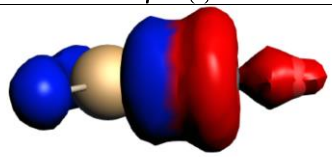	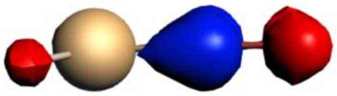
$\Delta E_{orb(1)}$ = −66.2 kcal/mol<br>$\lvert\nu_1\rvert$ = 0.98	$\Delta E_{orb(2)}$ = −69.4 kcal/mol<br>$\lvert\nu_2\rvert$ = 0.64	$\Delta E_{orb(3)}$ = −-8.8 kcal/mol<br>$\lvert\nu_3\rvert$ = 0.23

Figure S16. Plot of deformation densities $\Delta\rho_{1\text{-}3}$ (isovalue = 0.003) of the pairwise orbital interaction and the orbital interaction energies ΔE_{orb} (in kcal/mol) and their eigenvalues ν (in e) of **1** at pyramidalization angle ($\angle_p$) 180 °. The fragmentation is SiH_2 (a_1^6 a_2^0 b_1^4 b_2^4) and CO (a_1^6 a_2^0 b_1^2 b_2^2), see Table S6. The direction of the charge flow is red→blue. The eigenvalues ν indicate the amount of donated (negative numbers) and accepted charge (positive numbers).

$\Delta\rho$ orb(1)	$\Delta\rho$ orb(2)	$\Delta\rho$ orb(3)
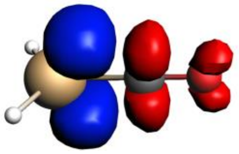	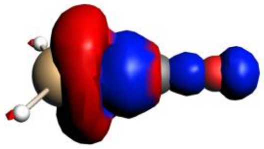	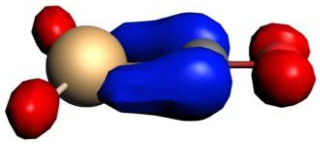
$\Delta E_{orb(1)\alpha\beta}$ = −46.5 kcal/mol<br>$\lvert\nu_1\rvert_\alpha$ = 0.66 ; $\lvert\nu_1\rvert_\beta$ = 0.50	$\Delta E_{orb(2)\alpha\beta}$ = −145.4 kcal/mol<br>$\lvert\nu_2\rvert_\alpha$ = 0.29; $\lvert\nu_2\rvert_\beta$ = 0.61	$\Delta E_{orb(3)\alpha\beta}$ = −6.9 kcal/mol<br>$\lvert\nu_3\rvert_\alpha$ = 0.10 ; $\lvert\nu_3\rvert_\beta$ = 0.10

Figure S17. Plot of deformation densities $\Delta\rho_{1\text{-}3}$ (isovalue = 0.003) of the pairwise orbital interaction and the orbital interaction energies ΔE_{orb} (in kcal/mol) and their eigenvalues ν (in e) of **1** at pyramidalization angle ($\angle_p$) 180 °. The fragmentation is SiH_2 (a_1^7 a_2^0 b_1^4 b_2^3) and CO (a_1^5 a_2^0 b_1^2 b_2^3), see Table S7. The direction of the charge flow is red→blue. The eigenvalues ν indicate the amount of donated (negative numbers) and accepted charge (positive numbers).

$\Delta\rho_{\text{orb(1)}}$	$\Delta\rho_{\text{orb(2)}}$	$\Delta\rho_{\text{orb(3)}}$
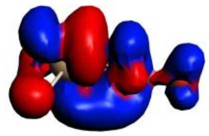<br>$\Delta E_{\text{orb(1)}} = -185.6$ kcal/mol<br>$\lvert\nu_1\rvert = 1.50$	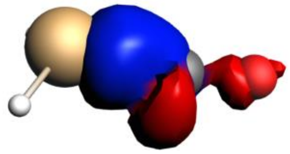<br>$\Delta E_{\text{orb(2)}} = -34.7$ kcal/mol<br>$\lvert\nu_2\rvert = 0.42$	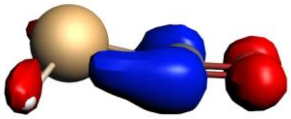<br>$\Delta E_{\text{orb(3)}} = -7.1$ kcal/mol<br>$\lvert\nu_3\rvert = 0.24$

Figure S18. Plot of deformation densities $\Delta\rho_{1\text{-}3}$ (isovalue = 0.003) of the pairwise orbital interaction and the orbital interaction energies ΔE_{orb} (in kcal/mol) and their eigenvalues ν (in e) of **1** at pyramidalization angle ($\angle_p$) 140 °. The fragmentation is SiH_2 ($a_1^8\ a_2^0\ b_1^4\ b_2^2$) and CO ($a_1^6\ a_2^0\ b_1^2\ b_2^2$), see Table S5. The direction of the charge flow is red→blue. The eigenvalues ν indicate the amount of donated (negative numbers) and accepted charge (positive numbers).

$\Delta\rho_{\text{orb(1)}}$	$\Delta\rho_{\text{orb(2)}}$	$\Delta\rho_{\text{orb(3)}}$
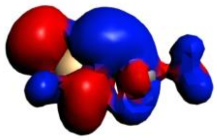<br>$\Delta E_{\text{orb(1)}} = -175.1$ kcal/mol<br>$\lvert\nu_1\rvert = 1.42$	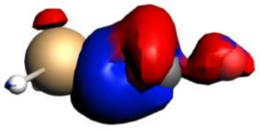<br>$\Delta E_{\text{orb(2)}} = -32.7$ kcal/mol<br>$\lvert\nu_2\rvert = 0.43$	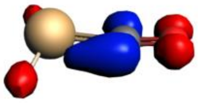<br>$\Delta E_{\text{orb(3)}} = -8.5$ kcal/mol<br>$\lvert\nu_3\rvert = 0.14$

Figure S19. Plot of deformation densities $\Delta\rho_{1\text{-}3}$ (isovalue = 0.003) of the pairwise orbital interaction and the orbital interaction energies ΔE_{orb} (in kcal/mol) and their eigenvalues ν (in e) of **1** at pyramidalization angle ($\angle_p$) 140 °. The fragmentation is SiH_2 ($a_1^6\ a_2^0\ b_1^4\ b_2^4$) and CO ($a_1^6\ a_2^0\ b_1^2\ b_2^2$), see Table S6. The direction of the charge flow is red→blue. The eigenvalues ν indicate the amount of donated (negative numbers) and accepted charge (positive numbers).

$\Delta\rho_{\text{orb(1)}}$	$\Delta\rho_{\text{orb(2)}}$	$\Delta\rho_{\text{orb(3)}}$
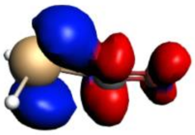<br>$\Delta E_{\text{orb(1)}\alpha\beta} = -50.9$ kcal/mol<br>$\lvert\nu_1\rvert_\alpha = 0.71$; $\lvert\nu_1\rvert_\beta = 0.47$	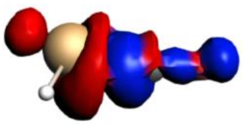<br>$\Delta E_{\text{orb(2)}\alpha\beta} = -140.6$ kcal/mol<br>$\lvert\nu_2\rvert_\alpha = 0.28$; $\lvert\nu_2\rvert_\beta = 0.63$	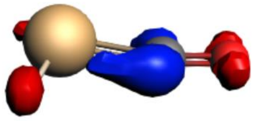<br>$\Delta E_{\text{orb(3)}\alpha\beta} = -6.3$ kcal/mol<br>$\lvert\nu_3\rvert_\alpha = 0.10$; $\lvert\nu_3\rvert_\beta = 0.11$

Figure S20. Plot of deformation densities $\Delta\rho_{1\text{-}3}$ (isovalue = 0.003) of the pairwise orbital interaction and the orbital interaction energies ΔE_{orb} (in kcal/mol) and their eigenvalues ν (in e) of **1** at pyramidalization angle ($\angle_p$) 140 °. The fragmentation is SiH_2 ($a_1^7\ a_2^0\ b_1^4\ b_2^3$) and CO ($a_1^5\ a_2^0\ b_1^2\ b_2^3$), see Table S7. The direction of the charge flow is red→blue. The eigenvalues ν indicate the amount of donated (negative numbers) and accepted charge (positive numbers).

xyz coordinate (Å) and Energy (in Hartree)

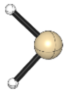

BP86-D3(BJ)/def2-svp
Thermal correction to Gibbs Energy = −0.008588
Thermal correction to Enthalpy = 0.015012
Sum of electronic and thermal Free Energies = −290.551389
Sum of electronic and thermal Enthalpies = −290.527789
N_{imag} = 0

Atomic Type	Coordinates (Angstroms) X	Y	Z
Si	-0.000000	-0.000000	0.136119
H	0.000000	1.100914	-0.952833
H	-0.000000	-1.100914	-0.952833

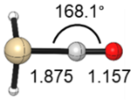

BP86-D3(BJ)/def2-svp
Thermal correction to Gibbs Energy = −0.004397
Thermal correction to Enthalpy = 0.026565
Sum of electronic and thermal Free Energies = −403.833646
Sum of electronic and thermal Enthalpies = −403.802685
N_{imag} = 0

Atomic Type	Coordinates (Angstroms) X	Y	Z
C	-0.000000	0.695551	-0.000000
O	-0.235890	1.828526	-0.000000
Si	-0.006536	-1.179549	0.000000
H	0.989313	-1.143917	1.162917
H	0.989313	-1.143917	-1.162917

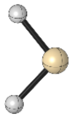

BP86-D3(BJ)/def2-svp
Thermal correction to Gibbs Energy = 0.040106
Thermal correction to Enthalpy = 0.074228
Sum of electronic and thermal Free Energies = −369.107327
Sum of electronic and thermal Enthalpies = −369.073204
N_{imag} = 0

Atomic Type	Coordinates (Angstroms) X	Y	Z
Si	-0.000001	-0.768619	-0.002948
C	-1.446304	0.502256	-0.003965
H	-2.447979	0.030248	-0.065730
H	-1.405238	1.094436	0.941493
H	-1.347467	1.242118	-0.831205
C	1.446322	0.502235	-0.003887
H	1.348575	1.240261	-0.832915
H	1.403936	1.096531	0.940166
H	2.448086	0.030133	-0.063424

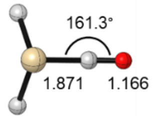

BP86-D3(BJ)/def2-svp
Thermal correction to Gibbs Energy = 0.047058
Thermal correction to Enthalpy = 0.087157
Sum of electronic and thermal Free Energies = −482.371624
Sum of electronic and thermal Enthalpies = −482.331525
N_{imag} = 0

Atomic Type	Coordinates (Angstroms) X	Y	Z
C	1.319115	0.000289	0.099044
O	2.483525	0.000417	0.151379
Si	-0.423988	0.000020	-0.580327
C	-1.157130	1.552770	0.299948
H	-0.557254	2.461449	0.088274
H	-2.168721	1.725192	-0.125551
H	-1.259930	1.432301	1.397624
C	-1.156028	-1.553353	0.299864
H	-1.258818	-1.433011	1.397559
H	-2.167614	-1.726063	-0.125549
H	-0.555765	-2.461722	0.088045

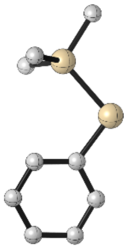

BP86-D3(BJ)/def2-svp
Thermal correction to Gibbs Energy = 0.152693
Thermal correction to Enthalpy = 0.211697
Sum of electronic and thermal Free Energies = −929.853048
Sum of electronic and thermal Enthalpies = −929.794044
N_{imag} = 0

Atomic Type	Coordinates (Angstroms) X	Y	Z
Si	-0.359275	-1.597196	-0.094596
Si	-2.076817	0.106949	0.017673
C	1.234415	-0.548829	-0.047830
C	2.450713	-1.287812	0.022190
C	1.338959	0.870242	-0.079634
C	3.700067	-0.650241	0.069848
H	2.400321	-2.390359	0.039413
C	2.583415	1.514369	-0.043814
H	0.425997	1.481558	-0.136292
C	3.766162	0.753863	0.034755
H	4.625050	-1.245553	0.129091
H	2.637322	2.614376	-0.073065
H	4.744121	1.260445	0.067078
C	-3.748865	-0.801561	0.085314
H	-3.808330	-1.463605	0.974903
H	-4.595828	-0.083721	0.136339
H	-3.893355	-1.438255	-0.812962
C	-1.943739	1.184896	1.589098
H	-1.975851	0.553522	2.501938
H	-1.004530	1.773430	1.622618
H	-2.798998	1.893564	1.639983
C	-2.098373	1.222837	-1.534511
H	-2.965753	1.917010	-1.488558
H	-1.177013	1.831180	-1.639569
H	-2.204403	0.613269	-2.456490

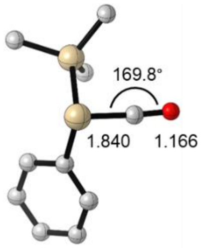

BP86-D3(BJ)/def2-svp
Thermal correction to Gibbs Energy = 0.152693
Thermal correction to Enthalpy = 0.211697
Sum of electronic and thermal Free Energies = −929.853048
Sum of electronic and thermal Enthalpies = −929.794044

$N_{imag} = 0$

Atomic Type	X	Y	Z
Si	-0.359275	-1.597196	-0.094596
Si	-2.076817	0.106949	0.017673
C	1.234415	-0.548829	-0.047830
C	2.450713	-1.287812	0.022190
C	1.338959	0.870242	-0.079634
C	3.700067	-0.650241	0.069848
H	2.400321	-2.390359	0.039413
C	2.583415	1.514369	-0.043814
H	0.425997	1.481558	-0.136292
C	3.766162	0.753863	0.034755
H	4.625050	-1.245553	0.129091
H	2.637322	2.614376	-0.073065
H	4.744121	1.260445	0.067078
C	-3.748865	-0.801561	0.085314
H	-3.808330	-1.463605	0.974903
H	-4.595828	-0.083721	0.136339
H	-3.893355	-1.438255	-0.812962
C	-1.943739	1.184896	1.589098
H	-1.975851	0.553522	2.501938
H	-1.004530	1.773430	1.622618
H	-2.798998	1.893564	1.639983
C	-2.098373	1.222837	-1.534511
H	-2.965753	1.917010	-1.488558
H	-1.177013	1.831180	-1.639569
H	-2.204403	0.613269	-2.456490

(Coordinates in Angstroms)

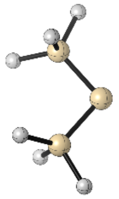

BP86-D3(BJ)/def2-svp
Thermal correction to Gibbs Energy = 0.171001
Thermal correction to Enthalpy = 0.234866
Sum of electronic and thermal Free Energies = −1107.460017
Sum of electronic and thermal Enthalpies = −1107.396153
$N_{imag} = 0$

Atomic Type	X	Y	Z
Si	0.000042	-1.603643	-0.002224
Si	-1.784830	-0.012019	0.010248

(Coordinates in Angstroms)

C	-3.409585	-0.857293	0.528633
H	-3.373713	-1.175739	1.591849
H	-4.275852	-0.170970	0.408191
H	-3.596304	-1.763152	-0.085081
C	-1.605900	1.562074	1.070435
H	-1.527426	1.306636	2.147861
H	-0.715297	2.161400	0.795803
H	-2.505470	2.202835	0.943133
C	-1.940654	0.476366	-1.835159
H	-2.842732	1.113687	-1.964284
H	-1.059717	1.048324	-2.188569
H	-2.064645	-0.412084	-2.489070
Si	1.784753	-0.011938	-0.010534
C	1.941417	0.468821	1.836851
H	2.843722	1.105370	1.968184
H	1.060781	1.039608	2.192906
H	2.065427	-0.422288	2.487131
C	3.409150	-0.855277	-0.533152
H	3.595931	-1.763773	0.076631
H	3.372799	-1.169180	-1.597702
H	4.275585	-0.169623	-0.410144
C	1.605606	1.566631	-1.064000
H	2.505418	2.206616	-0.934523
H	1.526464	1.315740	-2.142447
H	0.715318	2.165059	-0.786382

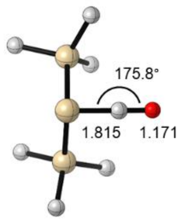

BP86-D3(BJ)/def2-svp
Thermal correction to Gibbs Energy = 0.177987
Thermal correction to Enthalpy = 0.245971
Sum of electronic and thermal Free Energies = −1220.759187
Sum of electronic and thermal Enthalpies = −1220.691203
N_{imag} = 0

Atomic Type	Coordinates (Angstroms) X	Y	Z
Si	-0.004319	0.399258	-1.331507
Si	1.843208	-0.357304	0.018050
C	3.345697	0.724849	-0.395198
H	3.181060	1.775039	-0.075883
H	4.253247	0.348038	0.124170
H	3.547062	0.727675	-1.486291

C	1.568901	-0.332974	1.896384
H	1.351417	0.695125	2.252487
H	0.726111	-0.987454	2.198552
H	2.481546	-0.691655	2.420064
C	2.163968	-2.141888	-0.550215
H	3.040441	-2.563447	-0.012121
H	1.290771	-2.795719	-0.350111
H	2.375828	-2.176729	-1.638823
Si	-1.875120	-0.308333	0.016476
C	-2.097860	0.571125	1.682878
H	-2.990941	0.168870	2.208517
H	-1.217028	0.429958	2.340393
H	-2.246929	1.661895	1.544616
C	-3.430726	-0.025705	-1.033810
H	-3.572527	1.054325	-1.246953
H	-3.357077	-0.555164	-2.006190
H	-4.335675	-0.393664	-0.503188
C	-1.643826	-2.173049	0.305795
H	-2.549507	-2.595794	0.791983
H	-1.485533	-2.710995	-0.651807
H	-0.775972	-2.380980	0.964362
C	0.061947	1.915628	-0.336517
O	0.122793	2.936506	0.233507

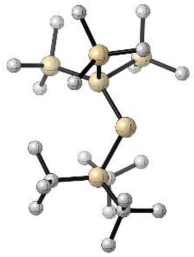

BP86-D3(BJ)/def2-svp
Thermal correction to Gibbs Energy = 0.606482
Thermal correction to Enthalpy = 0.731640
Sum of electronic and thermal Free Energies = −2568.358837
Sum of electronic and thermal Enthalpies = −2568.233678
N_{imag} = 0

Atomic Type	Coordinates (Angstroms) X	Y	Z
Si	0.458890	1.077117	0.849286
Si	2.455207	0.040571	0.026860
Si	-1.487322	0.059019	0.037128
Si	-2.268688	-1.838953	-1.171052
Si	-2.658356	1.950950	-0.791290
Si	-2.322087	-0.190125	2.254738
C	2.624457	-1.910268	0.151873
C	3.696397	-2.444659	-0.822509

C	1.264653	-2.554106	-0.175279
C	2.986852	-2.349617	1.586083
C	2.360488	0.693533	-1.834150
C	3.733499	0.820728	-2.520088
C	1.456544	-0.226890	-2.674849
C	1.663419	2.075260	-1.802282
C	3.868675	0.955463	1.050768
C	5.276418	0.392000	0.765357
C	3.864549	2.466982	0.734646
C	3.555887	0.823457	2.560155
C	-2.131984	-3.451558	-0.169072
C	-4.128902	-1.543133	-1.468338
C	-1.504077	-2.121577	-2.889584
C	-2.483975	1.912606	-2.688001
C	-1.907229	3.573436	-0.148809
C	-4.502465	1.948357	-0.327290
C	-4.034404	-1.017851	2.286878
C	-1.079031	-1.283621	3.199957
C	-2.467292	1.484482	3.138811
H	3.786543	-3.550964	-0.720758
H	4.698510	-2.013085	-0.626643
H	3.440648	-2.236281	-1.881016
H	1.342834	-3.664023	-0.110851
H	0.899460	-2.303045	-1.187484
H	0.485857	-2.224853	0.541809
H	3.995686	-2.014812	1.897510
H	2.978358	-3.462048	1.645903
H	2.253130	-1.976811	2.331487
H	4.257865	-0.154770	-2.577355
H	4.403077	1.532975	-1.997729
H	3.609764	1.191560	-3.564343
H	1.883563	-1.239924	-2.813817
H	1.311306	0.209360	-3.689729
H	0.454451	-0.334701	-2.212889
H	2.235155	2.851036	-1.261427
H	0.647904	2.007909	-1.329616
H	1.477398	2.447637	-2.836831
H	5.371146	-0.672285	1.061405
H	6.042103	0.959268	1.343411
H	5.550536	0.468532	-0.306642
H	4.156664	2.687606	-0.311398
H	4.595074	2.988574	1.394597
H	2.869581	2.924598	0.923692
H	4.291420	1.415018	3.152366
H	3.602671	-0.219458	2.924602
H	2.546320	1.225511	2.800600
H	-1.083264	-3.774744	-0.025642
H	-2.669710	-4.262678	-0.706173
H	-2.596175	-3.344963	0.832438
H	-4.573616	-2.442922	-1.946573
H	-4.304071	-0.681284	-2.144017

H	-4.673169	-1.354095	-0.521860
H	-2.094083	-2.903098	-3.415805
H	-0.453300	-2.468174	-2.840416
H	-1.533431	-1.199707	-3.505370
H	-2.952473	1.004860	-3.121103
H	-1.414118	1.913291	-2.983993
H	-2.968579	2.800401	-3.148815
H	-0.858631	3.703496	-0.486593
H	-1.901251	3.601476	0.959266
H	-2.499608	4.438285	-0.519835
H	-4.631667	2.017887	0.772513
H	-5.018273	1.031843	-0.674550
H	-5.010466	2.825428	-0.783939
H	-4.025097	-2.027290	1.829141
H	-4.779117	-0.401155	1.743431
H	-4.378113	-1.123299	3.338929
H	-0.088436	-0.780029	3.257937
H	-0.929468	-2.256308	2.686615
H	-1.414166	-1.488653	4.239635
H	-1.495428	2.018980	3.132823
H	-2.785254	1.336669	4.193596
H	-3.217933	2.135001	2.644445

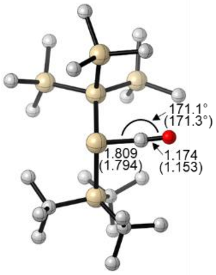

BP86-D3(BJ)/def2-svp
Thermal correction to Gibbs Energy = 0.615096
Thermal correction to Enthalpy = 0.743667
Sum of electronic and thermal Free Energies = −2681.646013
Sum of electronic and thermal Enthalpies = −2681.517442
N_{imag} = 0

Atomic Type	Coordinates (Angstroms) X	Y	Z
Si	0.448352	-0.386976	-1.101423
Si	2.562634	0.061091	-0.064782
Si	-1.674633	0.015663	-0.064139
Si	-2.263943	2.302845	0.097140
Si	-2.344206	-1.065778	1.929295
Si	-3.037447	-0.846154	-1.812892
O	0.302423	-3.322578	-0.643287
C	0.374722	-2.152895	-0.714308

C	2.962056	1.796024	-0.920380
C	4.111051	2.545677	-0.211084
C	1.706270	2.691758	-0.899583
C	3.327957	1.587792	-2.405525
C	2.413712	0.194736	1.885234
C	3.793204	0.309955	2.569148
C	1.560437	1.425503	2.245756
C	1.670900	-1.038231	2.437485
C	3.842727	-1.330628	-0.619654
C	5.289450	-0.788617	-0.546592
C	3.758607	-2.575199	0.291810
C	3.560167	-1.803213	-2.065039
C	-2.206895	3.102314	-1.625477
C	-4.053029	2.357148	0.748444
C	-1.272747	3.368313	1.319442
C	-1.876713	-0.047780	3.465690
C	-1.656380	-2.829844	2.068789
C	-4.243052	-1.218463	1.894486
C	-4.828099	-0.202894	-1.755607
C	-2.304330	-0.369813	-3.499470
C	-3.093044	-2.744003	-1.689448
H	4.329143	3.496850	-0.748395
H	5.050990	1.959897	-0.184745
H	3.850456	2.816168	0.832143
H	1.909430	3.644895	-1.438234
H	1.383060	2.950157	0.124132
H	0.844006	2.210468	-1.411997
H	4.279877	1.038831	-2.542476
H	3.446161	2.578452	-2.900574
H	2.530320	1.039036	-2.950014
H	4.375050	1.182127	2.210509
H	4.413276	-0.596390	2.417789
H	3.658690	0.431618	3.668316
H	2.053582	2.382973	1.985427
H	1.369109	1.445786	3.342733
H	0.573902	1.394506	1.740050
H	2.208589	-1.988140	2.264303
H	0.668447	-1.131394	1.977000
H	1.529714	-0.931171	3.537157
H	5.469525	0.039029	-1.260566
H	6.005194	-1.602124	-0.805278
H	5.556560	-0.427266	0.466946
H	4.071795	-2.361373	1.332869
H	4.445172	-3.360448	-0.098448
H	2.743315	-3.016791	0.321928
H	4.329077	-2.551243	-2.365477
H	3.588602	-0.981859	-2.804447
H	2.571978	-2.296847	-2.157655
H	-1.193761	3.037804	-2.072539
H	-2.489418	4.175470	-1.567749
H	-2.913577	2.598574	-2.316547

H	-4.424814	3.404225	0.712359
H	-4.094280	2.023095	1.805828
H	-4.744719	1.724907	0.160081
H	-1.786209	4.349146	1.420495
H	-0.236935	3.563471	0.983708
H	-1.228971	2.902653	2.324663
H	-2.355951	0.952867	3.432281
H	-0.782541	0.101663	3.546469
H	-2.224383	-0.560504	4.388442
H	-0.550866	-2.858057	2.120053
H	-1.966630	-3.442781	1.198482
H	-2.055338	-3.310248	2.988305
H	-4.578103	-1.872252	1.063367
H	-4.739774	-0.234834	1.778178
H	-4.597954	-1.671955	2.845393
H	-4.874309	0.888612	-1.947974
H	-5.312584	-0.402927	-0.778907
H	-5.426378	-0.709045	-2.543960
H	-1.289067	-0.799286	-3.624610
H	-2.214315	0.731014	-3.603913
H	-2.951547	-0.745180	-4.321470
H	-2.081673	-3.191549	-1.759478
H	-3.708462	-3.160766	-2.515926
H	-3.544246	-3.074765	-0.731214

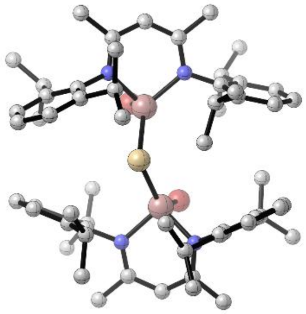

BP86-D3(BJ)/def2-svp

Thermal correction to Gibbs Energy = 1.127678

Thermal correction to Enthalpy = 1.320086

Sum of electronic and thermal Free Energies = −11763.684031

Sum of electronic and thermal Enthalpies = −11763.491623

$N_{imag} = 0$

Atomic Type	Coordinates (Angstroms) X	Y	Z
Br	-2.437960	0.165215	-2.480669
Ga	-1.877304	0.605557	-0.197060
Si	-0.077778	-0.461330	1.068072
N	-3.539471	-0.015220	0.757956
N	-2.335221	2.545945	-0.035112

C	-4.666633	0.708172	0.751839
C	-4.698694	2.072123	0.378981
H	-5.685724	2.550588	0.424303
C	-3.614624	2.944250	0.138716
C	-5.972191	0.068678	1.171087
H	-5.820847	-0.704875	1.947854
H	-6.425984	-0.437959	0.292910
H	-6.692595	0.824197	1.536339
C	-3.922296	4.427209	0.139334
H	-4.989736	4.607918	0.360203
H	-3.673019	4.893940	-0.833544
H	-3.303340	4.950035	0.897266
C	-3.473395	-1.298444	1.393502
C	-3.827759	-2.481205	0.692592
C	-3.687769	-3.716591	1.358312
H	-3.954241	-4.642894	0.825852
C	-3.222599	-3.784955	2.675963
H	-3.124862	-4.760024	3.178702
C	-2.874926	-2.607893	3.355947
H	-2.507380	-2.669483	4.390660
C	-2.981596	-1.351058	2.732856
C	-4.358988	-2.449195	-0.735309
H	-4.399656	-1.388520	-1.056262
C	-3.403618	-3.170941	-1.699699
H	-3.367645	-4.259757	-1.488529
H	-2.373896	-2.775062	-1.619411
H	-3.733270	-3.032456	-2.749879
C	-5.777978	-3.044047	-0.829492
H	-6.483093	-2.554144	-0.127600
H	-5.778415	-4.129486	-0.593057
H	-6.179225	-2.928107	-1.857949
C	-2.617698	-0.068823	3.476117
H	-2.227055	0.629806	2.703934
C	-3.852903	0.602862	4.108953
H	-4.613078	0.871269	3.351063
H	-3.560647	1.537174	4.633337
H	-4.331138	-0.071365	4.850653
C	-1.327594	3.565743	-0.154002
C	-1.150742	4.269507	-1.376949
C	-0.284084	5.381011	-1.381048
H	-0.150106	5.949179	-2.314666
C	0.418842	5.766587	-0.232719
H	1.083026	6.644662	-0.260031
C	0.308310	5.004827	0.938590
H	0.902251	5.274068	1.824793
C	-0.563342	3.901869	0.996676
C	-0.636222	3.029747	2.241286
H	-1.605579	2.490491	2.216280
C	-0.565650	3.802940	3.567528
H	-1.340595	4.594944	3.609601
H	0.424136	4.282655	3.715514

H	-0.727404	3.115440	4.423157
C	0.493865	1.991125	2.182318
H	0.458175	1.280142	3.027291
H	1.494155	2.466164	2.170380
H	0.476977	1.469545	1.165474
C	-1.820942	3.824589	-2.671651
H	-2.566782	3.043560	-2.418725
C	-2.547873	4.966787	-3.404256
H	-3.084781	4.570991	-4.290840
H	-1.840303	5.741777	-3.768117
H	-3.293963	5.474436	-2.757423
C	-0.774202	3.161318	-3.590059
H	-1.260149	2.761338	-4.503772
H	-0.266829	2.313757	-3.088837
H	0.001625	3.893806	-3.900691
Br	1.752843	0.176002	-2.833328
Ga	1.762006	-0.526343	-0.536092
N	3.580092	0.013221	0.094263
N	2.276654	-2.457839	-0.726640
C	4.674943	-0.650049	-0.330256
C	4.631609	-1.918651	-0.942576
H	5.598625	-2.323247	-1.269476
C	3.534309	-2.812887	-1.023240
C	6.030466	-0.019067	-0.103955
H	6.207483	0.162627	0.975282
H	6.082191	0.973057	-0.596040
H	6.841792	-0.659075	-0.495517
C	3.835808	-4.230282	-1.458817
H	4.864086	-4.523278	-1.175199
H	3.757561	-4.298033	-2.564358
H	3.114602	-4.954632	-1.035921
C	3.719962	1.162087	0.939524
C	3.762640	2.465916	0.373619
C	3.832783	3.572180	1.244523
H	3.848179	4.587390	0.818111
C	3.893776	3.401627	2.634823
H	3.956346	4.278499	3.298409
C	3.878210	2.108325	3.176781
H	3.930066	1.976342	4.269324
C	3.781002	0.972126	2.349132
C	3.743549	2.684592	-1.132627
H	3.736547	1.688468	-1.618186
C	2.461790	3.410428	-1.566977
H	2.446432	4.452144	-1.190913
H	1.552808	2.904037	-1.185691
H	2.381741	3.437275	-2.672133
C	4.992806	3.443706	-1.619556
H	5.932728	2.937299	-1.315681
H	5.027885	4.478116	-1.216089
H	4.989163	3.517590	-2.726844
C	3.763273	-0.419519	2.973906

H	3.613600	-1.149650	2.152516
C	2.592403	-0.585615	3.957280
H	2.607330	0.186771	4.754345
H	2.637638	-1.576556	4.452688
H	1.619880	-0.522886	3.425448
C	5.108091	-0.748341	3.651883
H	5.957046	-0.651422	2.945168
H	5.106372	-1.789018	4.038150
H	5.306190	-0.069952	4.508763
C	1.218063	-3.424938	-0.651836
C	0.575243	-3.893429	-1.831628
C	-0.452832	-4.846975	-1.692372
H	-0.954409	-5.224218	-2.597042
C	-0.864716	-5.299134	-0.434822
H	-1.681223	-6.032981	-0.349973
C	-0.244906	-4.805604	0.719549
H	-0.583448	-5.156148	1.703805
C	0.812825	-3.878549	0.640644
C	1.558521	-3.447929	1.902873
H	1.840351	-2.381797	1.762537
C	2.867894	-4.240977	2.087731
H	3.569344	-4.085642	1.245773
H	2.664827	-5.329735	2.170881
H	3.386434	-3.918073	3.015257
C	0.686183	-3.528936	3.161742
H	1.225034	-3.107309	4.032469
H	0.424795	-4.576627	3.420284
H	-0.255049	-2.959075	3.028139
C	0.945018	-3.404745	-3.227845
H	1.789813	-2.693075	-3.129099
C	1.383008	-4.562980	-4.146358
H	1.740843	-4.168260	-5.120009
H	0.538449	-5.251985	-4.360386
H	2.197227	-5.169729	-3.700425
C	-0.223060	-2.626147	-3.860775
H	0.079104	-2.207493	-4.842951
H	-0.542313	-1.777545	-3.225862
H	-1.102883	-3.284401	-4.024369
C	-1.510957	-0.265246	4.521513
H	-1.184035	0.716642	4.922460
H	-0.630626	-0.770584	4.075226
H	-1.856143	-0.867007	5.388495

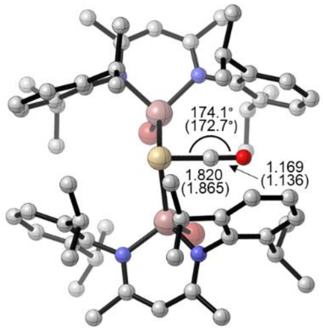

BP86-D3(BJ)/def2-svp
Thermal correction to Gibbs Energy = 1.131568
Thermal correction to Enthalpy = 1.331767
Sum of electronic and thermal Free Energies = −11876.977208
Sum of electronic and thermal Enthalpies = −11876.777009
N_{imag} = 0

Atomic Type	Coordinates (Angstroms) X	Y	Z
Br	1.729861	0.809670	-2.750640
Ga	1.965525	0.091735	-0.493447
Si	0.034241	-0.069239	0.991205
O	-0.046325	-2.986261	0.363790
C	-0.011365	-1.831121	0.536721
N	3.277707	1.380603	0.281157
N	3.277372	-1.401136	-0.603520
C	4.585371	1.277316	-0.021427
C	5.154486	0.145303	-0.647963
H	6.221543	0.223836	-0.892297
C	4.579661	-1.131816	-0.821696
C	5.518710	2.410042	0.343972
H	5.340690	2.758850	1.380065
H	5.338552	3.283425	-0.314821
H	6.575061	2.102977	0.238599
C	5.492500	-2.262384	-1.239843
H	6.535919	-1.914576	-1.346085
H	5.153709	-2.690714	-2.204892
H	5.460467	-3.093565	-0.507569
C	2.819967	2.500841	1.054790
C	2.567454	3.749773	0.425805
C	2.162439	4.831780	1.233497
H	1.953026	5.803390	0.759711
C	2.030311	4.693552	2.619370
H	1.726725	5.554473	3.235564
C	2.267012	3.449605	3.221801
H	2.137986	3.344396	4.308531
C	2.645263	2.328598	2.458352
C	2.698526	3.943483	-1.079956
H	3.113890	3.010489	-1.511224
C	1.311747	4.152967	-1.708547

H	0.879958	5.123878	-1.394623
H	0.602936	3.358797	-1.406130
H	1.376134	4.139421	-2.815404
C	3.634154	5.109990	-1.451235
H	4.639799	5.009849	-0.992742
H	3.219077	6.086294	-1.122597
H	3.765634	5.160822	-2.551972
C	2.883712	0.972985	3.120501
H	2.540971	0.204424	2.392642
C	4.378226	0.709816	3.391914
H	4.967977	0.657067	2.456625
H	4.509923	-0.257838	3.919290
H	4.810997	1.507453	4.032200
C	2.857840	-2.775094	-0.518606
C	2.416182	-3.467770	-1.676345
C	1.993960	-4.803921	-1.529328
H	1.633793	-5.350882	-2.414751
C	2.023490	-5.445241	-0.285088
H	1.688981	-6.490489	-0.193738
C	2.468390	-4.748380	0.845630
H	2.477152	-5.251180	1.825352
C	2.880973	-3.406198	0.755772
C	3.326350	-2.662445	2.011376
H	3.420400	-1.590183	1.743350
C	4.714070	-3.140623	2.481987
H	5.482642	-2.996169	1.696251
H	4.696931	-4.220081	2.743043
H	5.042023	-2.578444	3.381371
C	2.289423	-2.769989	3.143843
H	2.633368	-2.208360	4.036328
H	2.128325	-3.821704	3.458926
H	1.307713	-2.355327	2.838610
C	2.369063	-2.809251	-3.049168
H	2.818635	-1.799576	-2.957195
C	3.177634	-3.595525	-4.098667
H	3.194855	-3.045734	-5.062544
H	2.734458	-4.594921	-4.294312
H	4.228746	-3.757054	-3.779441
C	0.913856	-2.611822	-3.507491
H	0.877253	-2.060767	-4.469437
H	0.331346	-2.018713	-2.776598
H	0.399627	-3.586365	-3.645615
Br	-2.178301	-0.616390	-2.613150
Ga	-2.050135	-0.040288	-0.310977
N	-3.418110	-1.229356	0.538388
N	-3.149027	1.607700	-0.209416
C	-4.725118	-0.930219	0.447741
C	-5.210006	0.326471	0.019589
H	-6.302927	0.416483	-0.030375
C	-4.494691	1.526479	-0.184558
C	-5.754831	-1.971450	0.827265

H	-5.403492	-2.614749	1.656170
H	-5.941963	-2.638636	-0.040188
H	-6.714498	-1.498678	1.106533
C	-5.307892	2.790355	-0.362074
H	-6.368608	2.616963	-0.105082
H	-5.253213	3.135824	-1.414338
H	-4.911651	3.617945	0.258171
C	-2.983933	-2.449037	1.164194
C	-2.953557	-3.671562	0.441869
C	-2.458770	-4.817079	1.097198
H	-2.420182	-5.770663	0.547863
C	-1.985167	-4.759924	2.412058
H	-1.584538	-5.664505	2.895687
C	-2.011746	-3.544108	3.108642
H	-1.636019	-3.503148	4.141272
C	-2.515526	-2.374746	2.508913
C	-3.376619	-3.783004	-1.018974
H	-3.727868	-2.784467	-1.351261
C	-2.175488	-4.155001	-1.910207
H	-1.782332	-5.162251	-1.656282
H	-1.349259	-3.431066	-1.795841
H	-2.474340	-4.159701	-2.978707
C	-4.522478	-4.796388	-1.210533
H	-5.393033	-4.573598	-0.560380
H	-4.191776	-5.829816	-0.973249
H	-4.870421	-4.793636	-2.264498
C	-2.604585	-1.071523	3.296150
H	-2.459214	-0.247100	2.566061
C	-1.513990	-0.928946	4.365471
H	-1.638607	-1.659234	5.192396
H	-1.554912	0.082524	4.818135
H	-0.504616	-1.062358	3.926806
C	-4.007086	-0.887065	3.910238
H	-4.794442	-0.840509	3.133413
H	-4.056886	0.056852	4.492146
H	-4.250496	-1.726320	4.595685
C	-2.526531	2.903191	-0.219551
C	-2.408906	3.647242	-1.425197
C	-1.899766	4.958161	-1.340569
H	-1.814987	5.557902	-2.260113
C	-1.475719	5.500895	-0.121186
H	-1.073663	6.525625	-0.083253
C	-1.539737	4.730017	1.046918
H	-1.180940	5.150195	1.997825
C	-2.077457	3.429001	1.023569
C	-2.257226	2.633963	2.311775
H	-2.271173	1.560928	2.028051
C	-3.615703	2.938577	2.973505
H	-4.464742	2.660222	2.319018
H	-3.705242	4.019627	3.212240
H	-3.723637	2.368680	3.920009

C	-1.099577	2.823613	3.295740
H	-1.219606	2.146000	4.165285
H	-1.046620	3.858877	3.693054
H	-0.128328	2.599312	2.815027
C	-2.785747	3.068879	-2.785264
H	-3.285432	2.093219	-2.613362
C	-3.749623	3.977275	-3.572249
H	-4.075954	3.473433	-4.505467
H	-3.264051	4.931620	-3.866838
H	-4.657804	4.236823	-2.989208
C	-1.519406	2.781295	-3.614957
H	-1.788631	2.292216	-4.573899
H	-0.824429	2.103361	-3.083111
H	-0.975036	3.721433	-3.846462
C	2.055002	0.778674	4.397944
H	2.156842	-0.262277	4.763422
H	0.980109	0.967562	4.211146
H	2.390142	1.445952	5.219766

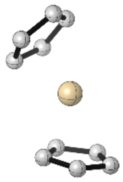

BP86-D3(BJ)/def2-svp
Thermal correction to Gibbs Energy = 0.123537
Thermal correction to Enthalpy = 0.173071
Sum of electronic and thermal Free Energies = −676.073920
Sum of electronic and thermal Enthalpies = −676.024386
N_{imag} = 0

Atomic Type	Coordinates (Angstroms) X	Y	Z
C	-2.523117	-1.011483	-0.500889
C	-1.833165	-0.957036	0.749840
C	-1.651616	0.434684	1.086831
C	-2.236021	1.212610	0.029684
C	-2.767104	0.315871	-0.936531
C	2.242158	-1.211801	0.018218
C	2.769055	-0.303337	-0.939155
C	2.518034	1.018597	-0.491113
C	1.828013	0.948673	0.758972
C	1.653587	-0.447193	1.082523
H	2.764200	1.942611	-1.030914
H	3.232959	-0.579854	-1.895100
H	2.256757	-2.307947	-0.046174
H	1.243972	-0.847173	2.019499
H	1.529978	1.797790	1.388213

H	-3.229280	0.603846	-1.889928
H	-1.538561	-1.813624	1.370525
H	-2.773636	-1.929000	-1.049691
H	-1.241005	0.823534	2.028045
H	-2.245635	2.309396	-0.023869
Si	0.000093	0.000208	-0.430063

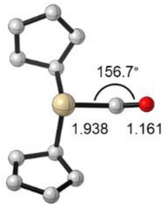

BP86-D3(BJ)/def2-svp
Thermal correction to Gibbs Energy = 0.379692
Thermal correction to Enthalpy = 0.468206
Sum of electronic and thermal Free Energies = −1181.985049
Sum of electronic and thermal Enthalpies = −1181.896536
N_{imag} = 0

Atomic Type	Coordinates (Angstroms) X	Y	Z
C	2.641917	0.610318	0.250921
C	1.587800	-0.227684	0.873804
C	1.862451	-1.605208	0.390336
C	2.896087	-1.544795	-0.527098
C	3.374425	-0.181239	-0.615059
C	-2.642351	0.610523	0.249793
C	-3.374227	-0.182091	-0.615770
C	-2.895513	-1.545392	-0.526330
C	-1.862250	-1.604622	0.391645
C	-1.588297	-0.226569	0.873940
H	-3.284015	-2.390592	-1.112211
H	-4.182131	0.161543	-1.277304
H	-2.779495	1.685197	0.435225
H	-1.304790	-0.059860	1.929604
H	-1.295386	-2.497802	0.687599
H	4.182560	0.1632630	-1.275862
H	1.304197	-0.062100	1.929635
H	2.778684	1.684845	0.437483
H	1.295807	-2.498884	0.685209
H	3.285149	-2.389337	-1.113557
Si	-0.000088	0.209355	-0.315294
C	0.000129	2.113236	0.045093
O	-0.000046	3.246737	-0.207419

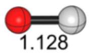

WB97XD/def2-svp
Thermal correction to Gibbs Energy = −0.013929
Thermal correction to Enthalpy = 0.007553
Sum of electronic and thermal Free Energies = −113.199306
Sum of electronic and thermal Enthalpies = −113.176880
N_{imag} = 0

Atomic Type	Coordinates (Angstroms) X	Y	Z
C	0.000000	0.000000	-0.644653
O	0.000000	0.000000	0.483489

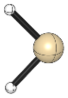

WB97XD/def2-svp
Thermal correction to Gibbs Energy = −0.007803
Thermal correction to Enthalpy = 0.015747
Sum of electronic and thermal Free Energies = −290.516331
Sum of electronic and thermal Enthalpies = −290.492781
N_{imag} = 0

Atomic Type	Coordinates (Angstroms) X	Y	Z
Si	0.000000	-0.000000	0.132884
H	0.000000	1.096898	-0.930187
H	-0.000000	-1.096898	-0.930187

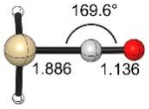

WB97XD/def2-svp
Thermal correction to Gibbs Energy = −0.003065
Thermal correction to Enthalpy = 0.027802
Sum of electronic and thermal Free Energies = −403.742745
Sum of electronic and thermal Enthalpies = −403.711878
N_{imag} = 0

Atomic Type	Coordinates (Angstroms) X	Y	Z
C	-0.000000	0.704893	0.000000
O	-0.189015	1.825069	0.000000
Si	-0.028369	-1.180963	-0.000000
H	0.954641	-1.148214	1.153190

H	0.954641	-1.148214	-1.153190

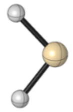

WB97XD/def2-svp
Thermal correction to Gibbs Energy = 0.043345
Thermal correction to Enthalpy = 0.077713
Sum of electronic and thermal Free Energies = −369.043543
Sum of electronic and thermal Enthalpies = −369.009175
N_{imag} = 0

Atomic Type	Coordinates (Angstroms) X	Y	Z
Si	-0.000010	-0.755561	-0.000004
C	-1.436280	0.496787	-0.011081
H	-2.408294	0.053092	-0.273383
H	-1.520798	0.874397	1.028100
H	-1.253481	1.380787	-0.645925
C	1.436276	0.496783	0.011079
H	1.521081	0.874252	-1.028127
H	1.253457	1.380888	0.645768
H	2.408197	0.053022	0.273629

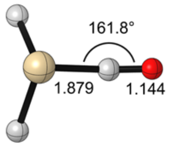

WB97XD/def2-svp
Thermal correction to Gibbs Energy = 0.050203
Thermal correction to Enthalpy = 0.089933
Sum of electronic and thermal Free Energies = −482.251587
Sum of electronic and thermal Enthalpies = −482.211857
N_{imag} = 0

Atomic Type	Coordinates (Angstroms) X	Y	Z
C	-1.323282	-0.000032	0.094099
O	-2.465671	-0.000028	0.152089
Si	0.429705	-0.000000	-0.582992
C	1.145998	-1.536828	0.302187
H	0.552369	-2.440315	0.094327
H	2.152436	-1.718087	-0.108753
H	1.244215	-1.416652	1.391627
C	1.145912	1.536871	0.302181
H	1.244147	1.416707	1.391622

H	2.152334	1.718195	-0.108769
H	0.552223	2.440320	0.094324

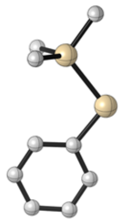

WB97XD/def2-svp
Thermal correction to Gibbs Energy = 0.161356
Thermal correction to Enthalpy = 0.218770
Sum of electronic and thermal Free Energies = −929.635251
Sum of electronic and thermal Enthalpies = −929.577837
N_{imag} = 0

Atomic Type	Coordinates (Angstroms) X	Y	Z
Si	-0.347016	-1.569681	0.000546
Si	-2.084133	0.104403	-0.000057
C	1.245117	-0.539025	0.000307
C	2.449622	-1.275995	-0.000028
C	1.355482	0.868276	0.000372
C	3.694305	-0.648787	-0.000261
H	2.403051	-2.369541	-0.000085
C	2.593879	1.502732	0.000153
H	0.454452	1.484364	0.000618
C	3.765562	0.743088	-0.000171
H	4.609309	-1.245237	-0.000521
H	2.649471	2.593633	0.000230
H	4.738275	1.240846	-0.000361
C	-3.735280	-0.817476	-0.000069
H	-3.832552	-1.461693	0.888337
H	-4.581367	-0.110786	-0.000303
H	-3.832367	-1.462068	-0.888221
C	-2.042326	1.194363	1.550938
H	-2.112746	0.581553	2.463777
H	-1.125761	1.798403	1.626449
H	-2.903656	1.882950	1.546882
C	-2.041515	1.193250	-1.551815
H	-2.903057	1.881577	-1.548945
H	-1.125068	1.797519	-1.626952
H	-2.110974	0.579810	-2.464304

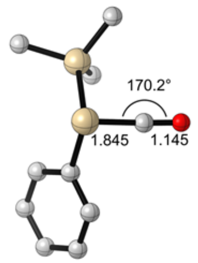

WB97XD/def2-svp
Thermal correction to Gibbs Energy = 0.167445
Thermal correction to Enthalpy = 0.229738
Sum of electronic and thermal Free Energies = −1042.859922
Sum of electronic and thermal Enthalpies = −1042.797629
N_{imag} = 0

Atomic Type	Coordinates (Angstroms) X	Y	Z
Si	0.349876	0.695906	-1.080173
Si	2.017809	-0.580929	0.053043
C	-1.365651	0.139441	-0.434762
C	-2.447487	1.032282	-0.360414
C	-1.607036	-1.205737	-0.112844
C	-3.709082	0.606708	0.051643
H	-2.308833	2.083288	-0.633037
C	-2.871473	-1.639220	0.285320
H	-0.797066	-1.938471	-0.172512
C	-3.925661	-0.732218	0.375896
H	-4.530659	1.324411	0.111413
H	-3.031653	-2.692104	0.529029
H	-4.915482	-1.068660	0.692050
C	3.628460	0.400010	-0.000072
H	3.554593	1.340761	0.567978
H	4.444559	-0.193130	0.444096
H	3.913418	0.647730	-1.034209
C	1.574953	-0.971215	1.843656
H	1.537693	-0.049923	2.445598
H	0.591851	-1.459722	1.921655
H	2.326685	-1.642030	2.291803
C	2.246584	-2.192532	-0.902909
H	3.048038	-2.792899	-0.441821
H	1.332283	-2.806229	-0.908811
H	2.526261	-1.994936	-1.949285
C	0.596480	2.090676	0.102664
O	0.805022	3.057883	0.678351

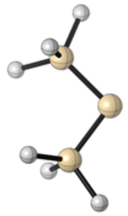

WB97XD/def2-svp
Thermal correction to Gibbs Energy = 0.180166
Thermal correction to Enthalpy = 0.242370
Sum of electronic and thermal Free Energies = −1107.264140
Sum of electronic and thermal Enthalpies = −1107.201936
N_{imag} = 0

Atomic Type	Coordinates (Angstroms) X	Y	Z
Si	0.000006	-1.582114	-0.000520
Si	-1.804772	-0.014636	0.005779
C	-3.404044	-0.884877	0.512502
H	-3.353793	-1.240161	1.554126
H	-4.266243	-0.202483	0.430399
H	-3.597269	-1.759946	-0.127628
C	-1.609800	1.501555	1.122501
H	-1.496582	1.208182	2.178344
H	-0.741640	2.118892	0.848408
H	-2.511121	2.132611	1.049754
C	-1.991042	0.550648	-1.795781
H	-2.876421	1.202248	-1.883181
H	-1.116735	1.119097	-2.145959
H	-2.141749	-0.302096	-2.476722
Si	1.804766	-0.014629	-0.005840
C	1.991006	0.549222	1.796178
H	2.876409	1.200717	1.884119
H	1.116711	1.117437	2.146770
H	2.141653	-0.304058	2.476460
C	3.404026	-0.884502	-0.513230
H	3.597223	-1.760082	0.126208
H	3.353788	-1.238956	-1.555138
H	4.266235	-0.202188	-0.430572
C	1.609852	1.502468	-1.121337
H	2.511211	2.133414	-1.048104
H	1.496588	1.209954	-2.177413
H	0.741734	2.119636	-0.846727

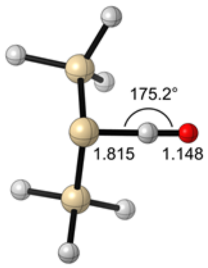

WB97XD/def2-svp
Thermal correction to Gibbs Energy = 0.186996
Thermal correction to Enthalpy = 0.253709
Sum of electronic and thermal Free Energies = −1220.506178
Sum of electronic and thermal Enthalpies = −1220.439464
N_{imag} = 0

Atomic Type	Coordinates (Angstroms) X	Y	Z
Si	-0.003207	0.439541	-1.248460
Si	1.865489	-0.363457	0.007060
C	3.354441	0.734935	-0.356567
H	3.194903	1.762277	0.007385
H	4.254319	0.338263	0.141669
H	3.556383	0.786429	-1.437478
C	1.605575	-0.425930	1.875988
H	1.393789	0.575511	2.282469
H	0.769693	-1.087479	2.151015
H	2.512897	-0.808117	2.372562
C	2.198418	-2.110113	-0.624265
H	3.069994	-2.542688	-0.106036
H	1.337733	-2.774239	-0.450451
H	2.410625	-2.105856	-1.704580
Si	-1.893448	-0.322672	0.005530
C	-2.115523	0.475201	1.701111
H	-2.998805	0.049943	2.205866
H	-1.240599	0.308818	2.346979
H	-2.270595	1.562302	1.616024
C	-3.444490	0.000343	-1.016357
H	-3.592443	1.078983	-1.182198
H	-3.377686	-0.483787	-2.002862
H	-4.337387	-0.389672	-0.500333
C	-1.669276	-2.186002	0.218592
H	-2.568982	-2.622482	0.682588
H	-1.513030	-2.685188	-0.750600
H	-0.810124	-2.422522	0.865202
C	0.050614	1.985101	-0.297898
O	0.095886	3.008815	0.220166

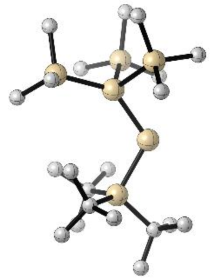

WB97XD/def2-svp
Thermal correction to Gibbs Energy = 0.637398
Thermal correction to Enthalpy = 0.757231
Sum of electronic and thermal Free Energies = −2567.822857
Sum of electronic and thermal Enthalpies = −2567.703025
N_{imag} = 0

Atomic Type	Coordinates (Angstroms) X	Y	Z
Si	-0.457294	-0.747162	1.151648
Si	-2.488365	-0.015084	0.092623
Si	1.532623	-0.031611	0.108267
Si	2.232491	1.558878	-1.516700
Si	2.509016	-2.108006	-0.503760
Si	2.625034	0.579113	2.125548
C	-2.666497	1.913671	-0.155060
C	-3.739961	2.255804	-1.203347
C	-1.322688	2.505298	-0.603764
C	-3.031570	2.624719	1.159591
C	-2.431183	-1.002560	-1.598896
C	-3.813878	-1.225340	-2.230398
C	-1.539994	-0.275748	-2.616029
C	-1.764557	-2.371351	-1.352429
C	-3.893119	-0.704813	1.273648
C	-5.295504	-0.213270	0.874633
C	-3.902467	-2.244224	1.266660
C	-3.620258	-0.286177	2.732513
C	2.168281	3.321614	-0.835445
C	4.052081	1.175922	-1.880357
C	1.382323	1.531033	-3.205037
C	2.190457	-2.388030	-2.347401
C	1.779613	-3.552593	0.469203
C	4.374509	-2.114453	-0.201894
C	4.368811	1.249879	1.839014
C	1.595842	1.930241	2.963047
C	2.751840	-0.898345	3.293442
H	-3.851531	3.352063	-1.282113
H	-4.726676	1.844665	-0.944559
H	-3.477111	1.887180	-2.205313
H	-1.428664	3.590069	-0.785852

H	-0.940681	2.051426	-1.525518
H	-0.550445	2.371909	0.169314
H	-4.039071	2.371719	1.516921
H	-3.009880	3.717503	1.002026
H	-2.315812	2.400598	1.966959
H	-4.336660	-0.277859	-2.429162
H	-4.469027	-1.843069	-1.599985
H	-3.706111	-1.749543	-3.197076
H	-1.953505	0.690835	-2.936606
H	-1.426119	-0.894704	-3.523886
H	-0.532668	-0.098876	-2.213041
H	-2.314938	-3.012747	-0.653430
H	-0.732055	-2.258622	-0.969909
H	-1.671080	-2.924943	-2.304166
H	-5.401800	0.876216	0.979158
H	-6.056041	-0.674094	1.530057
H	-5.555883	-0.475913	-0.161401
H	-4.207232	-2.664289	0.297827
H	-4.624955	-2.611297	2.016794
H	-2.919281	-2.664732	1.534104
H	-4.388016	-0.727921	3.392451
H	-3.651340	0.799453	2.886015
H	-2.642744	-0.652878	3.086270
H	1.139469	3.675792	-0.679916
H	2.656264	4.006884	-1.548089
H	2.702488	3.402073	0.123895
H	4.464060	1.965047	-2.531308
H	4.164256	0.217586	-2.410926
H	4.666426	1.134817	-0.969569
H	1.964875	2.169696	-3.889721
H	0.354262	1.918099	-3.185001
H	1.356670	0.517296	-3.633125
H	2.671426	-1.609348	-2.960000
H	1.113172	-2.375261	-2.576070
H	2.593154	-3.363428	-2.666502
H	0.709168	-3.701989	0.259508
H	1.884177	-3.395114	1.553458
H	2.308247	-4.483202	0.203152
H	4.602654	-2.016124	0.870759
H	4.885483	-1.298438	-0.732611
H	4.804587	-3.068579	-0.549121
H	4.371801	2.153159	1.210167
H	5.011809	0.497237	1.357050
H	4.825740	1.514034	2.807133
H	0.595757	1.552370	3.235484
H	1.458134	2.799523	2.300501
H	2.079953	2.282184	3.888926
H	1.762108	-1.342516	3.480262
H	3.179003	-0.583253	4.259789
H	3.402628	-1.684143	2.879066

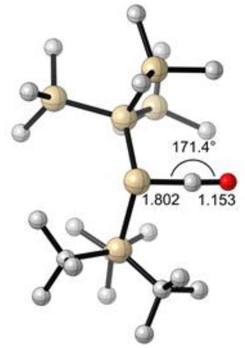

WB97XD/def2-svp
Thermal correction to Gibbs Energy = 0.645254
Thermal correction to Enthalpy = 0.769049
Sum of electronic and thermal Free Energies = −2681.059046
Sum of electronic and thermal Enthalpies = −2680.935252
N_{imag} = 0

Atomic Type	Coordinates (Angstroms) X	Y	Z
Si	0.428652	-0.475222	-0.989472
Si	2.575624	0.059820	-0.083511
Si	-1.716128	0.012929	-0.077345
Si	-2.295691	2.306171	-0.012524
Si	-2.343640	-0.950670	1.986617
Si	-3.099605	-0.941911	-1.754538
O	0.311338	-3.384703	-0.536019
C	0.368786	-2.235311	-0.609517
C	2.981348	1.723933	-1.038610
C	4.154729	2.494564	-0.407101
C	1.755834	2.654241	-1.047118
C	3.314679	1.439551	-2.514064
C	2.495529	0.317354	1.849327
C	3.889586	0.453007	2.487093
C	1.681014	1.579193	2.173629
C	1.760784	-0.854924	2.520214
C	3.830712	-1.363945	-0.572051
C	5.280296	-0.839648	-0.549989
C	3.759944	-2.552780	0.404312
C	3.544176	-1.925240	-1.979095
C	-2.273369	3.034424	-1.754208
C	-4.056114	2.410785	0.674167
C	-1.269347	3.402861	1.131633
C	-1.837833	0.146890	3.439584
C	-1.636873	-2.687206	2.204894
C	-4.228409	-1.116973	2.018371
C	-4.876915	-0.300382	-1.679064
C	-2.419379	-0.548978	-3.470102
C	-3.154958	-2.820292	-1.543558

H	4.384328	3.386843	-1.015858
H	5.074672	1.896187	-0.346535
H	3.918213	2.850745	0.606246
H	1.987060	3.570915	-1.617370
H	1.454301	2.964725	-0.040838
H	0.882684	2.189611	-1.534679
H	4.255822	0.887842	-2.642108
H	3.425938	2.395667	-3.054842
H	2.513101	0.872505	-3.013917
H	4.482372	1.267211	2.046461
H	4.477118	-0.472704	2.404595
H	3.784226	0.671202	3.564533
H	2.182626	2.505004	1.858018
H	1.526213	1.653735	3.264565
H	0.685204	1.550115	1.705852
H	2.265239	-1.820225	2.390359
H	0.740303	-0.956351	2.127373
H	1.682374	-0.670368	3.606413
H	5.468213	-0.087916	-1.328876
H	5.976190	-1.676362	-0.737022
H	5.557020	-0.397573	0.418332
H	4.108224	-2.290090	1.412834
H	4.416742	-3.361216	0.038621
H	2.749217	-2.976109	0.493433
H	4.325323	-2.659839	-2.243232
H	3.538999	-1.156422	-2.761264
H	2.580364	-2.452081	-2.030524
H	-1.279602	2.949938	-2.220625
H	-2.545551	4.102234	-1.727105
H	-2.995286	2.518971	-2.406804
H	-4.414205	3.450988	0.598216
H	-4.074849	2.132648	1.739908
H	-4.769334	1.766631	0.141964
H	-1.767247	4.384066	1.204985
H	-0.249009	3.574958	0.765781
H	-1.204136	2.983726	2.147196
H	-2.344981	1.123317	3.383790
H	-0.753989	0.330778	3.463942
H	-2.124449	-0.325448	4.393570
H	-0.537980	-2.703115	2.234996
H	-1.956451	-3.348144	1.384487
H	-2.005947	-3.119055	3.149849
H	-4.581692	-1.815671	1.244028
H	-4.733959	-0.152783	1.860228
H	-4.550732	-1.511982	2.996063
H	-4.929771	0.770863	-1.928301
H	-5.332399	-0.444789	-0.687813
H	-5.491803	-0.843403	-2.415772
H	-1.415466	-0.979097	-3.607031
H	-2.340235	0.536977	-3.633768
H	-3.083877	-0.963779	-4.246009

H	-2.157013	-3.275895	-1.628179
H	-3.792497	-3.265656	-2.325133
H	-3.574642	-3.107084	-0.566549

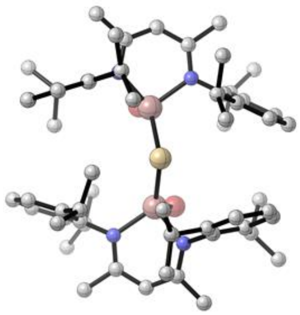

WB97XD/def2-svp
Thermal correction to Gibbs Energy = 1.180925
Thermal correction to Enthalpy = 1.367306
Sum of electronic and thermal Free Energies = −11761.781950
Sum of electronic and thermal Enthalpies = −11761.595568
N_{imag} = 0

Atomic Type	Coordinates (Angstroms) X	Y	Z
Br	-2.412431	-0.224601	-2.537172
Ga	-2.007156	0.309595	-0.255032
Si	-0.018272	-0.398509	0.968049
N	-3.503893	-0.616506	0.692845
N	-2.793165	2.120137	-0.072854
C	-4.730846	-0.114959	0.674744
C	-5.013958	1.214014	0.310817
H	-6.064631	1.496364	0.346107
C	-4.110620	2.267361	0.097701
C	-5.903228	-0.979507	1.064398
H	-5.644973	-1.696089	1.854204
H	-6.211957	-1.561802	0.182085
H	-6.756979	-0.371794	1.387234
C	-4.695516	3.658847	0.117384
H	-5.771494	3.627529	0.321013
H	-4.528665	4.173652	-0.837285
H	-4.203863	4.267044	0.890859
C	-3.227663	-1.860563	1.349073
C	-3.328080	-3.083978	0.659956
C	-2.999958	-4.260129	1.344480
H	-3.063247	-5.215928	0.818926
C	-2.602083	-4.235548	2.672800
H	-2.354969	-5.165191	3.190281
C	-2.513193	-3.019629	3.343817
H	-2.192762	-3.008722	4.386851
C	-2.808171	-1.817184	2.700616

C	-3.786934	-3.174586	-0.785622
H	-4.025314	-2.159091	-1.130543
C	-2.668816	-3.703285	-1.686702
H	-2.433405	-4.753566	-1.453385
H	-1.745902	-3.119009	-1.568650
H	-2.964930	-3.643190	-2.745048
C	-5.049391	-4.033050	-0.929500
H	-5.857740	-3.691303	-0.266182
H	-4.849279	-5.088790	-0.686269
H	-5.418855	-3.997424	-1.965845
C	-2.701766	-0.496064	3.448718
H	-2.464354	0.268404	2.695279
C	-4.034058	-0.095015	4.093482
H	-4.832846	0.023826	3.348994
H	-3.930948	0.864640	4.624232
H	-4.359060	-0.854191	4.822860
C	-1.994894	3.311938	-0.162763
C	-1.922109	4.040265	-1.368838
C	-1.272356	5.278198	-1.353678
H	-1.219912	5.861701	-2.275441
C	-0.680683	5.775247	-0.199978
H	-0.184460	6.748135	-0.213078
C	-0.683603	5.007769	0.958132
H	-0.171673	5.374765	1.849961
C	-1.335748	3.773792	0.995550
C	-1.262358	2.911498	2.241512
H	-2.071112	2.170748	2.182575
C	-1.451303	3.676765	3.552046
H	-2.384863	4.258471	3.540069
H	-0.619812	4.370327	3.750709
H	-1.497967	2.974137	4.397966
C	0.072580	2.163642	2.245231
H	0.143430	1.447578	3.073651
H	0.924906	2.855612	2.309831
H	0.235173	1.663710	1.253576
C	-2.475448	3.507890	-2.680976
H	-3.075024	2.613379	-2.461857
C	-3.375999	4.508702	-3.412456
H	-3.819397	4.033742	-4.300463
H	-2.813913	5.389437	-3.761039
H	-4.200822	4.870598	-2.779800
C	-1.316679	3.062717	-3.582317
H	-1.702510	2.595147	-4.500777
H	-0.673451	2.324319	-3.083078
H	-0.690973	3.923755	-3.870051
Br	1.796618	0.423679	-2.857935
Ga	1.869172	-0.235803	-0.564405
N	3.515717	0.627851	0.106768
N	2.703553	-2.046971	-0.665946
C	4.706321	0.175695	-0.297703
C	4.907510	-1.086126	-0.868163

H	5.926625	-1.313189	-1.177211
C	3.998904	-2.165412	-0.919807
C	5.916225	1.056390	-0.115849
H	6.027775	1.371306	0.930452
H	5.796116	1.974471	-0.709222
H	6.831786	0.545413	-0.434311
C	4.580714	-3.506243	-1.291795
H	5.631607	-3.574458	-0.985337
H	4.538925	-3.615425	-2.386438
H	4.017818	-4.339885	-0.854578
C	3.451214	1.775088	0.960074
C	3.290232	3.066028	0.416756
C	3.207220	4.152396	1.293800
H	3.068274	5.156238	0.885530
C	3.311879	3.980376	2.669067
H	3.257629	4.843022	3.336905
C	3.489991	2.703436	3.189802
H	3.578720	2.572776	4.271142
C	3.551496	1.583480	2.355971
C	3.210562	3.304539	-1.080490
H	3.413620	2.351047	-1.585422
C	1.803536	3.744419	-1.486524
H	1.575722	4.742816	-1.086814
H	1.032923	3.052968	-1.112256
H	1.706579	3.778484	-2.581682
C	4.254997	4.316303	-1.564493
H	5.275049	4.031924	-1.263810
H	4.062525	5.325611	-1.167108
H	4.232032	4.385215	-2.662617
C	3.765656	0.207177	2.969652
H	3.693750	-0.533774	2.161229
C	2.686159	-0.135687	3.998812
H	2.640659	0.605347	4.812085
H	2.897286	-1.113094	4.457464
H	1.694119	-0.195892	3.525884
C	5.166384	0.079784	3.580404
H	5.952517	0.281875	2.838691
H	5.326049	-0.938421	3.967560
H	5.304849	0.783398	4.416852
C	1.859387	-3.205690	-0.587621
C	1.351437	-3.803944	-1.760704
C	0.544227	-4.937838	-1.628686
H	0.143040	-5.410739	-2.527926
C	0.224706	-5.458475	-0.384212
H	-0.417158	-6.338629	-0.304285
C	0.713497	-4.847129	0.763249
H	0.445491	-5.257959	1.736410
C	1.541689	-3.723575	0.691669
C	2.124812	-3.126889	1.967260
H	2.192177	-2.040012	1.814663
C	3.546731	-3.634344	2.235614

H	4.242172	-3.361549	1.429942
H	3.560665	-4.730844	2.342961
H	3.937112	-3.196870	3.168334
C	1.230680	-3.350217	3.186775
H	1.612745	-2.782227	4.045972
H	1.201987	-4.408578	3.490743
H	0.201655	-3.016397	2.989221
C	1.635066	-3.276581	-3.158636
H	2.327481	-2.427598	-3.073728
C	2.290708	-4.338021	-4.051146
H	2.594728	-3.891735	-5.010266
H	1.594042	-5.160321	-4.278967
H	3.180976	-4.783619	-3.583151
C	0.354183	-2.745132	-3.809935
H	0.580587	-2.295369	-4.788570
H	-0.122909	-1.968761	-3.196572
H	-0.377524	-3.554441	-3.966853
C	-1.575047	-0.474955	4.482911
H	-1.431930	0.549360	4.861263
H	-0.627240	-0.816126	4.040605
H	-1.800422	-1.107986	5.355601

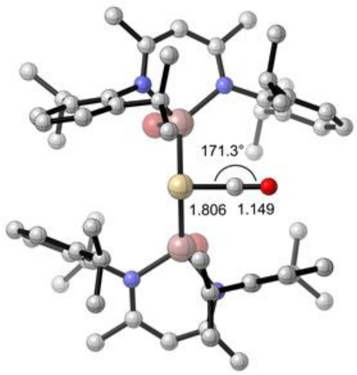

WB97XD/def2-svp
Thermal correction to Gibbs Energy = 1.186781
Thermal correction to Enthalpy = 1.379204
Sum of electronic and thermal Free Energies = −11875.025824
Sum of electronic and thermal Enthalpies = −11874.833401
N_{imag} = 0

Atomic Type	Coordinates (Angstroms) X	Y	Z
Br	1.851762	1.028868	-2.703570
Ga	2.028803	0.253679	-0.469941
Si	-0.029339	0.027837	0.778520
O	0.277644	-2.837200	1.397907
C	0.152201	-1.748145	1.053713
N	3.193150	1.595516	0.412642
N	3.433583	-1.125475	-0.560342

C	4.496212	1.601780	0.147167
C	5.157289	0.565245	-0.538031
H	6.204321	0.745451	-0.775394
C	4.689726	-0.733156	-0.785176
C	5.341211	2.765284	0.600028
H	5.132519	3.024270	1.646899
H	5.104287	3.654776	-0.000281
H	6.408427	2.542351	0.489396
C	5.683166	-1.716394	-1.351306
H	6.713739	-1.411714	-1.133984
H	5.554874	-1.738215	-2.445212
H	5.518855	-2.736701	-0.984242
C	2.636935	2.634291	1.231242
C	2.308504	3.885126	0.671750
C	1.817544	4.882099	1.521839
H	1.550528	5.854982	1.102924
C	1.667293	4.660106	2.883050
H	1.294783	5.455554	3.532188
C	1.968397	3.410648	3.414898
H	1.817159	3.234964	4.480979
C	2.434153	2.373459	2.604808
C	2.432977	4.175393	-0.815023
H	2.942421	3.326169	-1.290505
C	1.042673	4.279664	-1.446584
H	0.516969	5.175551	-1.084823
H	0.417738	3.408421	-1.202867
H	1.115837	4.338439	-2.542955
C	3.247843	5.439406	-1.111474
H	4.241546	5.418328	-0.639114
H	2.734953	6.346975	-0.756039
H	3.391772	5.549055	-2.197041
C	2.726046	1.003509	3.198022
H	2.532602	0.271427	2.400910
C	4.196148	0.855769	3.605512
H	4.870191	0.924826	2.739750
H	4.368158	-0.123709	4.077756
H	4.482981	1.635341	4.329240
C	3.118604	-2.525099	-0.506342
C	2.712345	-3.218991	-1.662302
C	2.363035	-4.567268	-1.532725
H	2.026561	-5.116760	-2.415138
C	2.445841	-5.220472	-0.310950
H	2.168986	-6.274058	-0.232443
C	2.888422	-4.531169	0.812695
H	2.954847	-5.054679	1.767732
C	3.224545	-3.178831	0.742480
C	3.721817	-2.443269	1.980643
H	3.494053	-1.377696	1.834925
C	5.245349	-2.560701	2.122750
H	5.775354	-2.118055	1.269219
H	5.547565	-3.617545	2.199268

H	5.589648	-2.041304	3.030362
C	3.039415	-2.887913	3.276482
H	3.346657	-2.230213	4.103688
H	3.328104	-3.911687	3.561620
H	1.944402	-2.854593	3.197313
C	2.690290	-2.574559	-3.038891
H	3.074616	-1.550332	-2.939263
C	3.603272	-3.320833	-4.019909
H	3.670934	-2.769260	-4.970114
H	3.215068	-4.325873	-4.249407
H	4.621627	-3.444450	-3.621596
C	1.270420	-2.463170	-3.595169
H	1.274947	-1.923664	-4.554201
H	0.609298	-1.908593	-2.915797
H	0.829070	-3.458320	-3.764534
Br	-2.104029	-0.748194	-2.787941
Ga	-2.084140	-0.233804	-0.476238
N	-3.367504	-1.506342	0.310260
N	-3.294551	1.328986	-0.347157
C	-4.670997	-1.334122	0.084232
C	-5.229643	-0.125805	-0.352779
H	-6.305947	-0.131630	-0.513922
C	-4.609803	1.137378	-0.419622
C	-5.612209	-2.485979	0.327272
H	-5.482499	-2.907501	1.332988
H	-5.387824	-3.294829	-0.383765
H	-6.656351	-2.180012	0.197252
C	-5.527450	2.325370	-0.566808
H	-6.569840	2.038113	-0.388915
H	-5.446365	2.735444	-1.583049
H	-5.251675	3.132825	0.124505
C	-2.937501	-2.640507	1.075777
C	-2.599748	-3.854112	0.445979
C	-2.183606	-4.922272	1.245185
H	-1.904430	-5.865689	0.770576
C	-2.107863	-4.805667	2.626306
H	-1.779302	-5.652969	3.231860
C	-2.446027	-3.602896	3.234016
H	-2.387207	-3.517272	4.321296
C	-2.860043	-2.503156	2.479263
C	-2.632573	-4.027807	-1.062031
H	-3.087913	-3.128912	-1.500515
C	-1.208900	-4.126548	-1.617117
H	-0.689640	-5.018676	-1.232064
H	-0.606068	-3.249960	-1.342077
H	-1.226819	-4.179737	-2.715876
C	-3.473607	-5.232905	-1.496043
H	-4.491603	-5.195234	-1.078804
H	-3.020975	-6.184827	-1.176395
H	-3.557241	-5.260891	-2.593084
C	-3.251053	-1.213684	3.186098

H	-3.341501	-0.430322	2.421265
C	-2.187789	-0.755165	4.186120
H	-2.043557	-1.481965	5.000272
H	-2.491607	0.194425	4.651359
H	-1.218880	-0.596979	3.688648
C	-4.622062	-1.345386	3.860191
H	-5.409450	-1.588948	3.132425
H	-4.904411	-0.401885	4.352353
H	-4.612874	-2.137739	4.625784
C	-2.795175	2.675293	-0.288918
C	-2.691525	3.452318	-1.462922
C	-2.316081	4.792194	-1.334546
H	-2.237842	5.411104	-2.231012
C	-2.019292	5.345828	-0.097230
H	-1.725968	6.395311	-0.021401
C	-2.067300	4.552480	1.041596
H	-1.802919	4.988097	2.005659
C	-2.458721	3.212360	0.973161
C	-2.570672	2.386150	2.246702
H	-2.363449	1.341772	1.974667
C	-3.987802	2.429242	2.829600
H	-4.727516	1.988634	2.145851
H	-4.293446	3.465441	3.046148
H	-4.031582	1.859580	3.770968
C	-1.529381	2.777043	3.293225
H	-1.539454	2.059430	4.125506
H	-1.721378	3.772393	3.724207
H	-0.518893	2.780487	2.861982
C	-2.934874	2.889621	-2.855112
H	-3.363081	1.882744	-2.750767
C	-3.911702	3.733921	-3.681851
H	-4.143034	3.221773	-4.627970
H	-3.484986	4.715958	-3.939507
H	-4.860686	3.916971	-3.155383
C	-1.603221	2.735564	-3.598471
H	-1.760415	2.251227	-4.574123
H	-0.891874	2.116264	-3.035604
H	-1.135112	3.718240	-3.772298
C	1.792967	0.641190	4.354154
H	1.912157	-0.420923	4.613650
H	0.741358	0.805368	4.077372
H	2.009223	1.224689	5.263053

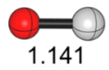

MP2/def2-svp
Thermal correction to Gibbs Energy = −0.014194
Thermal correction to Enthalpy = 0.008254
Sum of electronic and thermal Free Energies = −112.947523

Sum of electronic and thermal Enthalpies = −112.925075
N_{imag} = 0

Atomic Type	Coordinates (Angstroms) X	Y	Z
C	0.000000	0.000000	-0.651831
O	0.000000	0.000000	0.488873

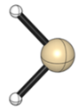

MP2/def2-svp
Thermal correction to Gibbs Energy = −0.007500
Thermal correction to Enthalpy = 0.016025
Sum of electronic and thermal Free Energies = −290.019640
Sum of electronic and thermal Enthalpies = −289.996116
N_{imag} = 0

Atomic Type	Coordinates (Angstroms) X	Y	Z
Si	0.000000	0.000000	0.109705
H	-0.000000	1.094539	-0.939626
H	-0.000000	-1.094539	-0.939626

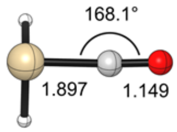

MP2/def2-svp
Thermal correction to Gibbs Energy = −0.003176
Thermal correction to Enthalpy = 0.027687
Sum of electronic and thermal Free Energies = −402.987126
Sum of electronic and thermal Enthalpies = −402.956263
N_{imag} = 0

Atomic Type	Coordinates (Angstroms) X	Y	Z
C	0.000000	0.710234	-0.000000
O	-0.235531	1.835097	-0.000000
Si	-0.003335	-1.187210	0.000000
H	0.965470	-1.160622	1.152263
H	0.965470	-1.160622	-1.152263

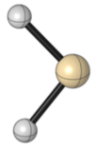

MP2/def2-svp
Thermal correction to Gibbs Energy = 0.044574
Thermal correction to Enthalpy = 0.078820
Sum of electronic and thermal Free Energies = −368.292922
Sum of electronic and thermal Enthalpies = −368.258675
N_{imag} = 0

Atomic Type	Coordinates (Angstroms) X	Y	Z
Si	-0.000023	-0.761734	-0.000010
C	-1.431056	0.500973	-0.010800
H	-2.406391	0.068630	-0.273000
H	-1.508453	0.878147	1.027345
H	-1.236847	1.379691	-0.646846
C	1.431026	0.500980	0.010785
H	1.509410	0.877652	-1.027457
H	1.236725	1.380023	0.646349
H	2.406050	0.068411	0.273841

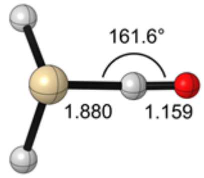

MP2/def2-svp
Thermal correction to Gibbs Energy = 0.050999
Thermal correction to Enthalpy = 0.090707
Sum of electronic and thermal Free Energies = −481.243711
Sum of electronic and thermal Enthalpies = −481.204002
N_{imag} = 0

Atomic Type	Coordinates (Angstroms) X	Y	Z
C	-1.315947	-0.000049	0.093688
O	-2.473484	-0.000040	0.151281
Si	0.436227	0.000001	-0.587665
C	1.143714	-1.541857	0.305815
H	0.540215	-2.437892	0.098909
H	2.148659	-1.732881	-0.100746
H	1.237621	-1.420181	1.394452
C	1.143592	1.541917	0.305808
H	1.237537	1.420253	1.394444

H	2.148507	1.733048	-0.100778
H	0.539997	2.437892	0.098917

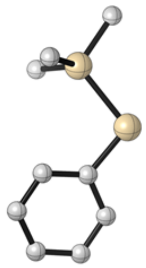

MP2/def2-svp
Thermal correction to Gibbs Energy = 0.161786
Thermal correction to Enthalpy = 0.219646
Sum of electronic and thermal Free Energies = −927.597523
Sum of electronic and thermal Enthalpies = −927.539664
N_{imag} = 0

Atomic Type	Coordinates (Angstroms) X	Y	Z
Si	-0.358076	-1.589555	-0.204114
Si	-2.067232	0.104889	0.035042
C	1.230913	-0.547259	-0.106749
C	2.435224	-1.280975	0.041962
C	1.339669	0.864275	-0.172058
C	3.676403	-0.644079	0.147288
H	2.390884	-2.374500	0.077582
C	2.579031	1.505641	-0.089959
H	0.443332	1.474002	-0.297345
C	3.749413	0.752234	0.077176
H	4.587534	-1.234746	0.271615
H	2.634782	2.595714	-0.149050
H	4.717441	1.254825	0.149840
C	-3.729591	-0.791600	0.180409
H	-3.744352	-1.467107	1.049382
H	-4.555599	-0.072256	0.297738
H	-3.933453	-1.398589	-0.714895
C	-1.841940	1.142081	1.608455
H	-1.849165	0.500152	2.503046
H	-0.898453	1.705817	1.611159
H	-2.672386	1.859560	1.709229
C	-2.185869	1.247578	-1.478606
H	-3.039549	1.933619	-1.354750
H	-1.284698	1.856515	-1.637901
H	-2.361527	0.664940	-2.396164

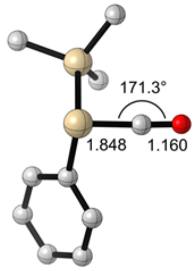

MP2/def2-svp
Thermal correction to Gibbs Energy = 0.167702
Thermal correction to Enthalpy = 0.230228
Sum of electronic and thermal Free Energies = −1040.568606
Sum of electronic and thermal Enthalpies = −1040.506080
N_{imag} = 0

Atomic Type	Coordinates (Angstroms) X	Y	Z
Si	0.382659	0.654734	-1.210129
Si	1.963168	-0.583168	0.098458
C	-1.328378	0.147365	-0.493434
C	-2.358235	1.080810	-0.254538
C	-1.620736	-1.217212	-0.289311
C	-3.612803	0.673081	0.213494
H	-2.183895	2.147144	-0.434238
C	-2.878819	-1.631626	0.166271
H	-0.857112	-1.976129	-0.486436
C	-3.878311	-0.686043	0.423115
H	-4.389867	1.418430	0.402790
H	-3.078012	-2.695808	0.318258
H	-4.859787	-1.005989	0.781418
C	3.581865	0.392323	0.138546
H	3.469052	1.349424	0.670188
H	4.359845	-0.188131	0.659594
H	3.943991	0.607139	-0.877982
C	1.373447	-0.902882	1.863308
H	1.281987	0.041931	2.420368
H	0.388655	-1.392778	1.871482
H	2.085442	-1.549970	2.401020
C	2.269843	-2.234656	-0.769482
H	3.031879	-2.811676	-0.221181
H	1.359444	-2.850139	-0.821449
H	2.631818	-2.078216	-1.796559
C	0.637153	2.040881	-0.014505
O	0.858104	3.001323	0.596916

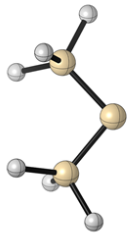

MP2/def2-svp
Thermal correction to Gibbs Energy = 0.183460
Thermal correction to Enthalpy = 0.245430
Sum of electronic and thermal Free Energies = −1105.010397
Sum of electronic and thermal Enthalpies = −1104.948428

Atomic Type	Coordinates (Angstroms) X	Y	Z
Si	0.000004	-1.589423	-0.000688
Si	-1.803509	-0.011990	0.006246
C	-3.406218	-0.878142	0.519769
H	-3.358761	-1.220360	1.564924
H	-4.267172	-0.196782	0.426961
H	-3.597282	-1.759624	-0.110511
C	-1.624370	1.521875	1.105494
H	-1.510638	1.243559	2.164465
H	-0.765412	2.146412	0.824724
H	-2.533762	2.139062	1.023026
C	-1.988024	0.525943	-1.807222
H	-2.875486	1.171679	-1.907385
H	-1.115050	1.091325	-2.162538
H	-2.133128	-0.338504	-2.473455
Si	1.803495	-0.011981	-0.006324
C	1.988117	0.523861	1.807760
H	2.875561	1.169516	1.908603
H	1.115151	1.088805	2.163792
H	2.133303	-0.341349	2.472986
C	3.406149	-0.877584	-0.520939
H	3.597236	-1.759782	0.108332
H	3.358619	-1.218615	-1.566478
H	4.267119	-0.196342	-0.427417
C	1.624359	1.523175	-1.103764
H	2.533764	2.140247	-1.020586
H	1.510602	1.246107	-2.163060
H	0.765418	2.147399	-0.822245

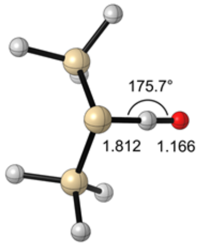

MP2/def2-svp
Thermal correction to Gibbs Energy = 0.189997
Thermal correction to Enthalpy = 0.256400
Sum of electronic and thermal Free Energies = −1218.000994
Sum of electronic and thermal Enthalpies = −1217.934591

Atomic Type	Coordinates (Angstroms) X	Y	Z
Si	-0.007106	0.375893	-1.323098
Si	1.846277	-0.363988	0.009789
C	3.325908	0.726415	-0.424038
H	3.149806	1.771511	-0.126474
H	4.229888	0.375280	0.099150
H	3.527437	0.711116	-1.505359
C	1.600552	-0.300003	1.883321
H	1.383023	0.725226	2.219686
H	0.778480	-0.950499	2.215480
H	2.519381	-0.634389	2.392299
C	2.195615	-2.145562	-0.512482
H	3.072492	-2.536200	0.028437
H	1.341434	-2.803573	-0.295897
H	2.404703	-2.203051	-1.591091
Si	-1.886117	-0.304111	0.009622
C	-2.111145	0.608543	1.649121
H	-3.000083	0.220697	2.172682
H	-1.242383	0.480067	2.309882
H	-2.259759	1.687500	1.490977
C	-3.436576	-0.035533	-1.034337
H	-3.569061	1.030170	-1.274790
H	-3.375636	-0.588307	-1.983574
H	-4.333300	-0.376821	-0.492251
C	-1.681627	-2.153527	0.346349
H	-2.589306	-2.549640	0.829788
H	-1.524911	-2.713938	-0.587812
H	-0.830660	-2.357095	1.012592
C	0.074291	1.890408	-0.330849
O	0.146700	2.907298	0.234422

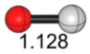

PBE0-D3(BJ)/def2-svp
Thermal correction to Gibbs Energy = −0.013940

Thermal correction to Enthalpy = 0.008487
Sum of electronic and thermal Free Energies = −113.110344
Sum of electronic and thermal Enthalpies = −113.087917

Atomic Type	Coordinates (Angstroms) X	Y	Z
C	0.000000	0.000000	-0.644610
O	0.000000	0.000000	0.483457

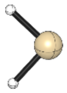

PBE0-D3(BJ)/def2-svp
Thermal correction to Gibbs Energy = −0.008092
Thermal correction to Enthalpy = 0.015474
Sum of electronic and thermal Free Energies = −290.394068
Sum of electronic and thermal Enthalpies = −290.370502

Atomic Type	Coordinates (Angstroms) X	Y	Z
Si	-0.000000	-0.000000	0.125575
H	0.000000	1.096154	-0.947561
H	-0.000000	-1.096154	-0.947561

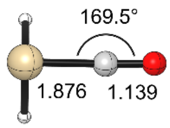

PBE0-D3(BJ)/def2-svp
Thermal correction to Gibbs Energy = −0.003432
Thermal correction to Enthalpy = 0.027417
Sum of electronic and thermal Free Energies = −403.541923
Sum of electronic and thermal Enthalpies = −403.511074

Atomic Type	Coordinates (Angstroms) X	Y	Z
C	-0.006529	0.685909	-0.000000
O	-0.215619	1.805144	-0.000000
Si	-0.004740	-1.189607	0.000000
H	0.982580	-1.128757	1.153769
H	0.982580	-1.128757	-1.153769

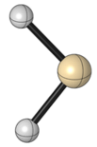

PBE0-D3(BJ)/def2-svp
Thermal correction to Gibbs Energy = 0.042884
Thermal correction to Enthalpy = 0.076546
Sum of electronic and thermal Free Energies = −368.852199
Sum of electronic and thermal Enthalpies = −368.818538

Atomic Type	Coordinates (Angstroms) X	Y	Z
Si	-0.000002	-0.760739	-0.002865
C	-1.435925	0.497476	-0.003694
H	-2.428732	0.027443	-0.061317
H	-1.392324	1.086784	0.932168
H	-1.339003	1.226092	-0.828470
C	1.435947	0.497451	-0.003596
H	1.340377	1.223761	-0.830586
H	1.390723	1.089393	0.930500
H	2.428857	0.027309	-0.058455

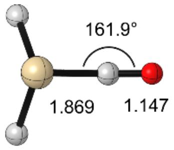

PBE0-D3(BJ)/def2-svp
Thermal correction to Gibbs Energy = 0.049962
Thermal correction to Enthalpy = 0.089755
Sum of electronic and thermal Free Energies = −481.981536
Sum of electronic and thermal Enthalpies = −481.941743

Atomic Type	Coordinates (Angstroms) X	Y	Z
C	-1.317629	-0.000043	0.092785
O	-2.462998	-0.000036	0.150499
Si	0.427682	-0.000018	-0.576030
C	1.144999	-1.541544	0.299123
H	0.542623	-2.439694	0.094342
H	2.145578	-1.726103	-0.124339
H	1.253489	-1.421655	1.387617
C	1.144878	1.541623	0.299020
H	1.253397	1.421814	1.387521
H	2.145433	1.726251	-0.124470
H	0.542416	2.439705	0.094193

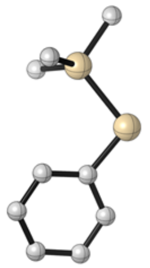

PBE0-D3(BJ)/def2-svp
Thermal correction to Gibbs Energy = 0.160558
Thermal correction to Enthalpy = 0.218233
Sum of electronic and thermal Free Energies = −929.113135
Sum of electronic and thermal Enthalpies = −929.055459

Atomic Type	Coordinates (Angstroms) X	Y	Z
Si	-0.348751	-1.577745	0.000435
Si	-2.075563	0.107777	0.000002
C	1.238933	-0.540899	0.000250
C	2.444371	-1.276370	0.000115
C	1.343000	0.866444	0.000187
C	3.686482	-0.646194	-0.000053
H	2.394557	-2.370122	0.000149
C	2.579343	1.503607	0.000020
H	0.437240	1.475931	0.000281
C	3.752915	0.746385	-0.000098
H	4.604223	-1.239346	-0.000148
H	2.633229	2.595128	-0.000023
H	4.724597	1.247142	-0.000234
C	-3.727134	-0.812591	0.000415
H	-3.821724	-1.456820	0.889002
H	-4.574445	-0.107257	-0.000037
H	-3.821646	-1.457892	-0.887399
C	-2.029279	1.195592	1.552890
H	-2.098640	0.579111	2.463188
H	-1.110416	1.796055	1.627048
H	-2.889647	1.885712	1.551911
C	-2.028709	1.193684	-1.554212
H	-2.888831	1.884111	-1.554308
H	-1.109600	1.793719	-1.628827
H	-2.098034	0.576131	-2.463787

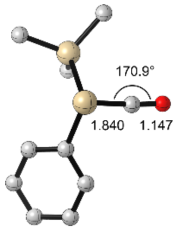

PBE0-D3(BJ)/def2-svp
Thermal correction to Gibbs Energy = 0.166476
Thermal correction to Enthalpy = 0.229166
Sum of electronic and thermal Free Energies = −1042.259825
Sum of electronic and thermal Enthalpies = −1042.197135

Atomic Type	Coordinates (Angstroms) X	Y	Z
Si	0.360484	0.663122	-1.151815
Si	1.983161	-0.587017	0.084943
C	-1.351442	0.148573	-0.465419
C	-2.393624	1.072930	-0.289339
C	-1.626341	-1.205373	-0.216248
C	-3.651120	0.664875	0.150113
H	-2.225542	2.133448	-0.501784
C	-2.887592	-1.618583	0.210704
H	-0.844964	-1.957848	-0.358193
C	-3.903009	-0.683420	0.402113
H	-4.442800	1.405338	0.290327
H	-3.076341	-2.678924	0.397066
H	-4.890803	-1.004920	0.740593
C	3.598792	0.385661	0.062326
H	3.509444	1.338617	0.606908
H	4.397772	-0.201146	0.544593
H	3.915652	0.608760	-0.968095
C	1.456401	-0.921158	1.862462
H	1.398199	0.019286	2.432038
H	0.465810	-1.398749	1.903799
H	2.179963	-1.583982	2.365607
C	2.236488	-2.222173	-0.823942
H	3.021877	-2.811322	-0.322034
H	1.320721	-2.833104	-0.841828
H	2.548664	-2.048920	-1.865238
C	0.644145	2.045405	0.028486
O	0.896891	2.993447	0.623113

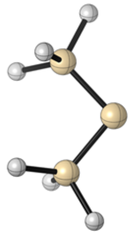

PBE0-D3(BJ)/def2-svp
Thermal correction to Gibbs Energy = 0.178823
Thermal correction to Enthalpy = 0.241702
Sum of electronic and thermal Free Energies = −1106.701294
Sum of electronic and thermal Enthalpies = −1106.638415

Atomic Type	Coordinates (Angstroms) X	Y	Z
Si	0.000009	-1.581972	-0.000915
Si	-1.795713	-0.009470	0.007082
C	-3.394146	-0.875047	0.524576
H	-3.345499	-1.206308	1.574066
H	-4.260338	-0.200139	0.422903
H	-3.576256	-1.765792	-0.096703
C	-1.615849	1.532813	1.089487
H	-1.518374	1.260295	2.152183
H	-0.744040	2.143760	0.814637
H	-2.517595	2.159882	0.989533
C	-1.969735	0.504015	-1.812750
H	-2.863944	1.140187	-1.924411
H	-1.098959	1.074554	-2.167555
H	-2.102236	-0.369268	-2.470830
Si	1.795683	-0.009444	-0.007181
C	1.970111	0.500902	1.813509
H	2.864381	1.136831	1.926065
H	1.099446	1.070897	2.169461
H	2.102697	-0.373504	2.470079
C	3.393954	-0.874211	-0.526519
H	3.576153	-1.766025	0.093200
H	3.345057	-1.203676	-1.576562
H	4.260201	-0.199520	-0.423890
C	1.615687	1.534742	-1.086845
H	2.517490	2.161579	-0.985955
H	1.518008	1.264091	-2.149999
H	0.743966	2.145270	-0.810779

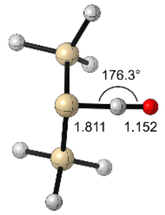

PBE0-D3(BJ)/def2-svp
Thermal correction to Gibbs Energy = 0.185898
Thermal correction to Enthalpy = 0.253059
Sum of electronic and thermal Free Energies = −1219.798932
Sum of electronic and thermal Enthalpies = −1219.866093

Atomic Type	Coordinates (Angstroms) X	Y	Z
Si	-0.005438	0.384348	-1.309630
Si	1.848589	-0.358248	0.015288
C	3.330726	0.725251	-0.412635
H	3.163571	1.768932	-0.102882
H	4.235574	0.361273	0.101636
H	3.525043	0.720132	-1.496084
C	1.595325	-0.319699	1.884755
H	1.378260	0.702040	2.233337
H	0.767396	-0.971801	2.201480
H	2.509950	-0.664918	2.395172
C	2.178290	-2.132326	-0.536057
H	3.054724	-2.538320	-0.004329
H	1.319312	-2.787819	-0.326848
H	2.382652	-2.173691	-1.616998
Si	-1.882645	-0.306795	0.013717
C	-2.107521	0.582474	1.661744
H	-2.997668	0.189533	2.180716
H	-1.238199	0.444915	2.321325
H	-2.253739	1.664126	1.516526
C	-3.425209	-0.028898	-1.034024
H	-3.563256	1.041025	-1.254996
H	-3.351939	-0.562774	-1.993980
H	-4.324182	-0.387402	-0.505461
C	-1.665803	-2.157618	0.318606
H	-2.569654	-2.566066	0.800183
H	-1.509329	-2.701051	-0.626190
H	-0.809181	-2.365168	0.977582
C	0.064789	1.907375	-0.331448
O	0.126249	2.912177	0.227863

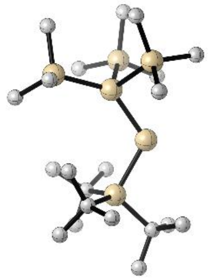

PBE0-D3(BJ)/def2-svp
Thermal correction to Gibbs Energy = 0.631019
Thermal correction to Enthalpy = 0.753932
Sum of electronic and thermal Free Energies = −2566.394365
Sum of electronic and thermal Enthalpies = −2566.271452

Atomic Type	Coordinates (Angstroms) X	Y	Z
Si	-0.446908	-1.074269	0.778550
Si	-2.471943	-0.043823	0.018016
Si	1.527953	-0.055350	0.037749
Si	2.269894	1.878672	-1.130175
Si	2.695021	-1.918825	-0.850679
Si	2.334137	0.136015	2.266642
C	-2.663693	1.888727	0.191894
C	-3.765221	2.428329	-0.727827
C	-1.335980	2.562138	-0.156693
C	-2.988335	2.289935	1.634801
C	-2.410436	-0.653178	-1.844216
C	-3.784361	-0.801001	-2.500181
C	-1.558229	0.296248	-2.688021
C	-1.684262	-2.006945	-1.857104
C	-3.836651	-0.995994	1.051835
C	-5.250266	-0.455915	0.812086
C	-3.819628	-2.490750	0.710646
C	-3.501640	-0.893395	2.547074
C	2.085536	3.465796	-0.118939
C	4.125828	1.634261	-1.418141
C	1.506196	2.154769	-2.836399
C	2.533886	-1.847347	-2.734996
C	1.959367	-3.550908	-0.252735
C	4.528038	-1.915388	-0.390332
C	4.024310	0.978608	2.336574
C	1.087857	1.180252	3.238293
C	2.487299	-1.551023	3.097441
H	-3.866046	3.520574	-0.591413
H	-4.747021	1.980698	-0.515194
H	-3.542866	2.256022	-1.790987
H	-1.431383	3.658354	-0.050356
H	-1.005428	2.356926	-1.181842
H	-0.533219	2.226290	0.516326

H	-3.976479	1.940747	1.964496
H	-2.990579	3.391875	1.714496
H	-2.236417	1.915474	2.347730
H	-4.331632	0.153340	-2.525903
H	-4.420465	-1.536861	-1.988008
H	-3.669815	-1.141134	-3.545997
H	-2.012596	1.290536	-2.803056
H	-1.430294	-0.121147	-3.703287
H	-0.554963	0.425595	-2.256319
H	-2.210758	-2.798650	-1.310390
H	-0.658474	-1.921678	-1.433226
H	-1.535386	-2.357482	-2.895443
H	-5.360047	0.589125	1.136644
H	-5.983525	-1.050395	1.387012
H	-5.545714	-0.509514	-0.246578
H	-4.130208	-2.697611	-0.323264
H	-4.523613	-3.026207	1.372814
H	-2.823579	-2.935389	0.870581
H	-4.219884	-1.497140	3.130879
H	-3.549456	0.132306	2.933227
H	-2.495734	-1.293213	2.762913
H	1.037698	3.777510	-0.013041
H	2.631319	4.279107	-0.625557
H	2.510652	3.355547	0.890068
H	4.543173	2.544627	-1.880384
H	4.325925	0.792529	-2.098083
H	4.668055	1.451547	-0.479042
H	2.084446	2.936176	-3.357142
H	0.461127	2.490685	-2.785122
H	1.541672	1.242105	-3.450303
H	2.997442	-0.938463	-3.148727
H	1.475465	-1.852763	-3.040105
H	3.025420	-2.718645	-3.198766
H	0.921610	-3.683456	-0.594760
H	1.952229	-3.609990	0.845592
H	2.558282	-4.390923	-0.643332
H	4.661567	-2.013806	0.697917
H	5.035765	-0.995276	-0.712583
H	5.033700	-2.770642	-0.868983
H	4.006084	1.991682	1.908517
H	4.778683	0.391465	1.791002
H	4.351649	1.060653	3.386547
H	0.107003	0.675940	3.284510
H	0.936490	2.164304	2.767388
H	1.424817	1.344874	4.275207
H	1.530446	-2.093796	3.065290
H	2.784217	-1.425278	4.151992
H	3.248796	-2.174355	2.604252

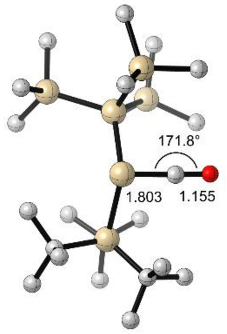

PBE0-D3(BJ)/def2-svp
Thermal correction to Gibbs Energy = 0.639528
Thermal correction to Enthalpy = 0.766157
Sum of electronic and thermal Free Energies = −2679.548665
Sum of electronic and thermal Enthalpies = −2679.422036

Atomic Type	Coordinates (Angstroms) X	Y	Z
Si	0.430659	-0.391774	-1.015738
Si	2.579220	0.063351	-0.067008
Si	-1.713935	0.018676	-0.058019
Si	-2.306720	2.307152	0.017462
Si	-2.354917	-0.991321	1.980318
Si	-3.058630	-0.916113	-1.779674
O	0.276837	-3.307132	-0.583428
C	0.352403	-2.156564	-0.653639
C	2.995807	1.749046	-0.980603
C	4.180122	2.484847	-0.342156
C	1.780582	2.681645	-0.950524
C	3.304569	1.489471	-2.459350
C	2.496099	0.262204	1.871413
C	3.885499	0.360606	2.512102
C	1.696571	1.519459	2.221232
C	1.743381	-0.920411	2.487696
C	3.805400	-1.367413	-0.607926
C	5.256292	-0.864845	-0.583838
C	3.719237	-2.574521	0.334815
C	3.491270	-1.876747	-2.021994
C	-2.253885	3.047633	-1.718378
C	-4.077046	2.405415	0.677170
C	-1.284999	3.386527	1.181066
C	-1.871631	0.075296	3.463009
C	-1.643651	-2.728667	2.170846
C	-4.239587	-1.161263	1.984647
C	-4.840130	-0.282735	-1.732888
C	-2.337717	-0.489344	-3.470233
C	-3.113112	-2.798041	-1.606405
H	4.403688	3.399567	-0.920043
H	5.097463	1.879805	-0.322281
H	3.963845	2.801928	0.688604

H	2.000482	3.598270	-1.526269
H	1.507810	2.990135	0.064954
H	0.892001	2.220573	-1.414631
H	4.232902	0.922132	-2.610704
H	3.425594	2.455076	-2.981948
H	2.484817	0.947006	-2.957644
H	4.480333	1.193968	2.111516
H	4.470874	-0.561467	2.385106
H	3.779924	0.527241	3.599234
H	2.209919	2.446660	1.929474
H	1.539002	1.567940	3.313625
H	0.702815	1.511517	1.748293
H	2.243338	-1.884410	2.332787
H	0.729740	-0.998836	2.071094
H	1.647770	-0.770701	3.578247
H	5.443279	-0.078397	-1.328069
H	5.937063	-1.701799	-0.821939
H	5.553323	-0.474799	0.400751
H	4.064616	-2.341220	1.351629
H	4.371561	-3.378496	-0.049924
H	2.704592	-2.990881	0.407422
H	4.242075	-2.634855	-2.308936
H	3.513615	-1.086621	-2.782422
H	2.506240	-2.361570	-2.082658
H	-1.253582	2.956847	-2.168791
H	-2.516849	4.117750	-1.685468
H	-2.969839	2.543029	-2.385075
H	-4.429989	3.448227	0.611037
H	-4.114660	2.109061	1.736979
H	-4.779204	1.771291	0.119319
H	-1.766935	4.376258	1.247912
H	-0.256726	3.538044	0.828661
H	-1.242873	2.963218	2.195707
H	-2.369300	1.056595	3.414740
H	-0.787164	0.247507	3.513093
H	-2.183608	-0.414620	4.400224
H	-0.545517	-2.737410	2.212872
H	-1.950519	-3.374758	1.334595
H	-2.022327	-3.177274	3.104305
H	-4.583178	-1.836435	1.186152
H	-4.743762	-0.192973	1.851658
H	-4.568589	-1.586363	2.947629
H	-4.891976	0.793128	-1.959303
H	-5.317539	-0.451839	-0.756367
H	-5.432946	-0.813313	-2.496513
H	-1.326731	-0.908979	-3.584736
H	-2.262250	0.599486	-3.612551
H	-2.979446	-0.897361	-4.268782
H	-2.112809	-3.249342	-1.678177
H	-3.733856	-3.225759	-2.411271
H	-3.553529	-3.103909	-0.644837

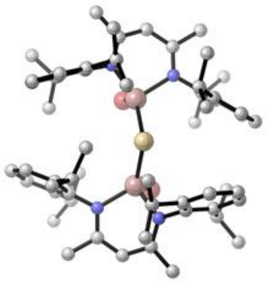

PBE0-D3(BJ)/def2-svp
Thermal correction to Gibbs Energy = 1.172887
Thermal correction to Enthalpy = 1.362620
Sum of electronic and thermal Free Energies = −11758.289060
Sum of electronic and thermal Enthalpies = −11758.099327

Atomic Type	Coordinates (Angstroms) X	Y	Z
Br	-2.446193	0.036270	-2.481163
Ga	-1.946055	0.526576	-0.210702
Si	-0.058491	-0.402807	1.026852
N	-3.555563	-0.186714	0.735073
N	-2.491861	2.426421	-0.063256
C	-4.703662	0.475924	0.719440
C	-4.807833	1.826400	0.344058
H	-5.810936	2.247527	0.386804
C	-3.777984	2.747951	0.108133
C	-5.975385	-0.212747	1.128654
H	-5.799773	-0.988004	1.885280
H	-6.405907	-0.709889	0.244801
H	-6.713597	0.507170	1.502896
C	-4.171293	4.199641	0.109041
H	-5.241830	4.313002	0.313739
H	-3.937298	4.678609	-0.850846
H	-3.599892	4.747595	0.873561
C	-3.444094	-1.457786	1.374557
C	-3.738574	-2.649675	0.682965
C	-3.568989	-3.865473	1.355644
H	-3.787914	-4.797259	0.828626
C	-3.133623	-3.908943	2.672103
H	-3.011019	-4.868990	3.179401
C	-2.848766	-2.724298	3.344524
H	-2.503088	-2.765341	4.378765
C	-2.987750	-1.486220	2.715310
C	-4.240173	-2.661041	-0.746994
H	-4.321359	-1.616925	-1.082931
C	-3.245789	-3.352050	-1.676271
H	-3.163331	-4.425487	-1.444379
H	-2.243568	-2.912041	-1.592978

H	-3.565331	-3.251246	-2.724796
C	-5.619483	-3.312819	-0.858515
H	-6.352788	-2.851760	-0.180595
H	-5.577391	-4.386263	-0.613764
H	-6.002169	-3.224728	-1.887244
C	-2.694019	-0.200528	3.465265
H	-2.344393	0.520493	2.708777
C	-3.954152	0.386003	4.104241
H	-4.728379	0.612829	3.359358
H	-3.717631	1.323524	4.632408
H	-4.383569	-0.316737	4.836186
C	-1.550152	3.496816	-0.181670
C	-1.402577	4.196483	-1.398305
C	-0.597736	5.340451	-1.407306
H	-0.487421	5.902279	-2.337668
C	0.069536	5.769696	-0.267526
H	0.683569	6.672956	-0.298399
C	-0.013302	5.020907	0.900724
H	0.553348	5.327792	1.782120
C	-0.816461	3.880647	0.961013
C	-0.836677	3.020852	2.205283
H	-1.758845	2.421695	2.178732
C	-0.835638	3.802804	3.514780
H	-1.658837	4.532200	3.541533
H	0.108019	4.348405	3.668961
H	-0.957902	3.118099	4.367922
C	0.353767	2.066862	2.162680
H	0.347513	1.357033	2.998930
H	1.313337	2.604326	2.173933
H	0.387127	1.554268	1.153608
C	-2.037634	3.723041	-2.690652
H	-2.735416	2.908241	-2.446951
C	-2.817244	4.817125	-3.418562
H	-3.319877	4.396079	-4.302539
H	-2.156903	5.624156	-3.773675
H	-3.589709	5.277467	-2.783306
C	-0.960535	3.130635	-3.602135
H	-1.416716	2.712530	-4.512459
H	-0.410164	2.317601	-3.108376
H	-0.233386	3.902198	-3.904479
Br	1.827294	0.260451	-2.809747
Ga	1.823236	-0.442642	-0.529843
N	3.580837	0.191578	0.130535
N	2.416207	-2.338581	-0.703416
C	4.697712	-0.412149	-0.283579
C	4.725300	-1.672038	-0.890686
H	5.706610	-2.025299	-1.204071
C	3.683457	-2.616969	-0.976514
C	6.014025	0.276830	-0.062484
H	6.164250	0.508520	1.001316
H	6.030139	1.239014	-0.594481

H	6.848506	-0.340175	-0.414766
C	4.079178	-4.004190	-1.399192
H	5.103208	-4.232419	-1.078136
H	4.050958	-4.062492	-2.498555
H	3.392872	-4.767162	-1.011470
C	3.668033	1.337211	0.975178
C	3.677753	2.635504	0.421129
C	3.713317	3.730868	1.290622
H	3.706671	4.740084	0.871768
C	3.768362	3.559246	2.669134
H	3.803911	4.428101	3.330937
C	3.786228	2.274284	3.200507
H	3.839780	2.140438	4.283964
C	3.730318	1.147719	2.374585
C	3.662675	2.871937	-1.074634
H	3.708036	1.891348	-1.569252
C	2.363331	3.538854	-1.513594
H	2.291451	4.563136	-1.120300
H	1.479807	2.985636	-1.163928
H	2.303606	3.585425	-2.611084
C	4.868111	3.690988	-1.537262
H	5.822633	3.239282	-1.226347
H	4.842770	4.715861	-1.133675
H	4.873350	3.769341	-2.635305
C	3.771973	-0.233888	3.000232
H	3.653652	-0.966808	2.188705
C	2.623991	-0.448462	3.983626
H	2.621349	0.309750	4.782321
H	2.711522	-1.433571	4.465683
H	1.651616	-0.415659	3.468486
C	5.120547	-0.501232	3.671047
H	5.958346	-0.375701	2.969676
H	5.160153	-1.530988	4.059377
H	5.290043	0.182688	4.518052
C	1.427979	-3.369773	-0.651218
C	0.838810	-3.866734	-1.834416
C	-0.115778	-4.882448	-1.720535
H	-0.578170	-5.277075	-2.628263
C	-0.499877	-5.381465	-0.484731
H	-1.256296	-6.167175	-0.419620
C	0.072032	-4.868874	0.672903
H	-0.243737	-5.257656	1.641129
C	1.047163	-3.869271	0.618482
C	1.723298	-3.394752	1.892703
H	1.945468	-2.324034	1.756779
C	3.055343	-4.111293	2.122573
H	3.766801	-3.934746	1.304501
H	2.905549	-5.199054	2.214439
H	3.526913	-3.754857	3.052438
C	0.826312	-3.516565	3.118053
H	1.305889	-3.046345	3.987883

H	0.635612	-4.567538	3.387082
H	-0.140839	-3.020261	2.951607
C	1.190022	-3.352634	-3.217087
H	1.982817	-2.597856	-3.108995
C	1.704933	-4.468905	-4.127907
H	2.050885	-4.049437	-5.085284
H	0.911407	-5.197526	-4.358230
H	2.539273	-5.026447	-3.677386
C	-0.006409	-2.651911	-3.859464
H	0.279810	-2.221776	-4.831507
H	-0.381782	-1.829322	-3.236497
H	-0.836268	-3.356771	-4.030741
C	-1.586060	-0.345810	4.502055
H	-1.315657	0.641852	4.907123
H	-0.686537	-0.795768	4.056127
H	-1.897729	-0.965226	5.357789

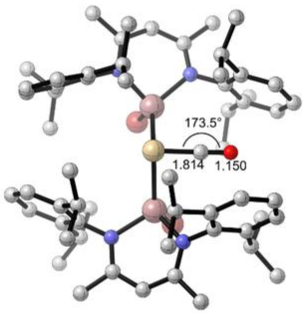

PBE0-D3(BJ)/def2-svp
Thermal correction to Gibbs Energy = 1.178360
Thermal correction to Enthalpy = 1.374884
Sum of electronic and thermal Free Energies = −11871.451120
Sum of electronic and thermal Enthalpies = −11871.254596

Atomic Type	Coordinates (Angstroms) X	Y	Z
Br	1.841821	0.838372	-2.737259
Ga	2.012832	0.070001	-0.503710
Si	0.016068	-0.086666	0.876713
O	-0.049339	-3.028702	0.568430
C	-0.023298	-1.880067	0.608051
N	3.322543	1.325946	0.301442
N	3.285197	-1.434146	-0.612425
C	4.613189	1.202862	0.001186
C	5.161988	0.084173	-0.649116
H	6.220011	0.153773	-0.895234
C	4.574651	-1.172911	-0.837717
C	5.570712	2.297650	0.378723
H	5.367797	2.680256	1.387985
H	5.454750	3.146580	-0.310787

H	6.607461	1.945999	0.323181
C	5.471564	-2.291502	-1.284207
H	6.499591	-1.939446	-1.426781
H	5.101298	-2.716703	-2.228201
H	5.471084	-3.113553	-0.554897
C	2.891170	2.439412	1.086631
C	2.678853	3.698168	0.486380
C	2.301656	4.767396	1.306021
H	2.125639	5.745920	0.853255
C	2.153502	4.609386	2.676358
H	1.869841	5.459910	3.300963
C	2.351902	3.358710	3.251387
H	2.214596	3.238075	4.326834
C	2.705620	2.252029	2.475885
C	2.822998	3.929923	-1.004616
H	3.217673	3.008546	-1.456904
C	1.457517	4.189416	-1.634774
H	1.044263	5.149312	-1.291683
H	0.734368	3.404985	-1.375274
H	1.535947	4.218148	-2.732058
C	3.779379	5.076752	-1.334436
H	4.767429	4.952290	-0.866276
H	3.379329	6.046046	-0.997196
H	3.926739	5.145496	-2.423215
C	2.915040	0.894754	3.122812
H	2.608087	0.141514	2.378705
C	4.386901	0.640254	3.452670
H	5.016780	0.630068	2.552518
H	4.507051	-0.335737	3.948543
H	4.775268	1.414581	4.133595
C	2.864729	-2.799506	-0.537778
C	2.377752	-3.463326	-1.682159
C	1.948493	-4.786993	-1.546724
H	1.552052	-5.307997	-2.421541
C	2.022675	-5.450818	-0.329236
H	1.683033	-6.485905	-0.246415
C	2.528458	-4.790897	0.783649
H	2.583890	-5.314975	1.740988
C	2.947776	-3.461270	0.706925
C	3.469255	-2.775396	1.956438
H	3.652711	-1.720605	1.704078
C	4.800692	-3.375304	2.411253
H	5.563604	-3.321261	1.620948
H	4.685987	-4.434425	2.692035
H	5.189907	-2.836037	3.289155
C	2.446102	-2.808784	3.091352
H	2.858150	-2.327746	3.991291
H	2.175419	-3.840206	3.364567
H	1.519652	-2.281403	2.821363
C	2.308871	-2.796072	-3.040489
H	2.782921	-1.807464	-2.953920

C	3.067625	-3.586087	-4.107125
H	3.078254	-3.028295	-5.056152
H	2.595738	-4.561687	-4.304366
H	4.111933	-3.779309	-3.816303
C	0.861683	-2.561785	-3.464554
H	0.820754	-2.006410	-4.413610
H	0.310640	-1.965987	-2.724417
H	0.325885	-3.514848	-3.600396
Br	-2.290390	-0.572644	-2.635843
Ga	-2.098779	-0.023243	-0.342631
N	-3.465027	-1.178214	0.516290
N	-3.141438	1.645945	-0.212352
C	-4.751198	-0.852605	0.416659
C	-5.208164	0.403157	-0.013179
H	-6.289826	0.511068	-0.073189
C	-4.473852	1.583806	-0.200352
C	-5.803597	-1.861465	0.781398
H	-5.490687	-2.496981	1.619685
H	-5.977744	-2.526073	-0.079034
H	-6.751436	-1.368095	1.027394
C	-5.269422	2.846423	-0.380570
H	-6.322245	2.684889	-0.122101
H	-5.213652	3.174132	-1.428932
H	-4.866568	3.668255	0.226592
C	-3.078732	-2.401166	1.149767
C	-3.068873	-3.617583	0.436856
C	-2.653416	-4.773001	1.106184
H	-2.633043	-5.720862	0.563300
C	-2.246066	-4.736720	2.431792
H	-1.913890	-5.650553	2.929961
C	-2.253649	-3.529618	3.120859
H	-1.929775	-3.504567	4.162554
C	-2.670225	-2.348432	2.503233
C	-3.442646	-3.722424	-1.028550
H	-3.769565	-2.728628	-1.369248
C	-2.224291	-4.103691	-1.869785
H	-1.858130	-5.109606	-1.608229
H	-1.398355	-3.397805	-1.723110
H	-2.479979	-4.098084	-2.940428
C	-4.583550	-4.713403	-1.263373
H	-5.465851	-4.488368	-0.645854
H	-4.275784	-5.744743	-1.028610
H	-4.893021	-4.696085	-2.319826
C	-2.726748	-1.053593	3.290178
H	-2.573046	-0.236952	2.568687
C	-1.630491	-0.939053	4.342383
H	-1.767055	-1.658044	5.165295
H	-1.642097	0.065771	4.790224
H	-0.636165	-1.098398	3.899917
C	-4.107004	-0.850933	3.918539
H	-4.899631	-0.794981	3.159696

H	-4.135683	0.085556	4.497053
H	-4.349924	-1.680195	4.602202
C	-2.511466	2.930137	-0.213239
C	-2.364512	3.666005	-1.408533
C	-1.840396	4.959282	-1.322430
H	-1.728477	5.547024	-2.236458
C	-1.443455	5.502663	-0.107805
H	-1.033748	6.514928	-0.067392
C	-1.548025	4.747079	1.053539
H	-1.213657	5.168722	2.002882
C	-2.087759	3.458743	1.025866
C	-2.278906	2.680726	2.312714
H	-2.296651	1.614832	2.040643
C	-3.624599	3.003119	2.964277
H	-4.470662	2.732055	2.317277
H	-3.700975	4.078458	3.192341
H	-3.739160	2.447850	3.908376
C	-1.132915	2.872309	3.295467
H	-1.251834	2.194036	4.153466
H	-1.094136	3.897014	3.697350
H	-0.163960	2.660250	2.823679
C	-2.728695	3.105790	-2.769837
H	-3.245262	2.146726	-2.614612
C	-3.657416	4.028313	-3.560019
H	-3.980135	3.534157	-4.489165
H	-3.151938	4.963620	-3.847926
H	-4.559186	4.305984	-2.993158
C	-1.466329	2.806000	-3.578648
H	-1.729012	2.333137	-4.537365
H	-0.793277	2.118461	-3.049106
H	-0.907809	3.731371	-3.793924
C	2.041039	0.685648	4.354180
H	2.119425	-0.354006	4.701828
H	0.983831	0.885703	4.130090
H	2.346805	1.329681	5.193677

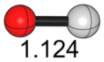

M06-2X/def2-svp
Thermal correction to Gibbs Energy = −0.013825
Thermal correction to Enthalpy = 0.008595
Sum of electronic and thermal Free Energies = −113.199466
Sum of electronic and thermal Enthalpies = −113.177046

Atomic Type	Coordinates (Angstroms) X	Y	Z
C	0.000000	0.000000	-0.642266
O	0.000000	0.000000	0.481699

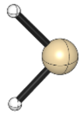

M06-2X/def2-svp
Thermal correction to Gibbs Energy = −0.007815
Thermal correction to Enthalpy = 0.015728
Sum of electronic and thermal Free Energies = −290.499524
Sum of electronic and thermal Enthalpies = −290.475981

Atomic Type	Coordinates (Angstroms) X	Y	Z
Si	-0.000000	0.000000	0.132952
H	0.000000	1.090815	-0.930662
H	-0.000000	-1.090815	-0.930662

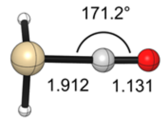

M06-2X/def2-svp
Thermal correction to Gibbs Energy = −0.003312
Thermal correction to Enthalpy = 0.027682
Sum of electronic and thermal Free Energies = −403.723730
Sum of electronic and thermal Enthalpies = −403.692736

Atomic Type	Coordinates (Angstroms) X	Y	Z
C	-0.018826	0.687737	-0.000000
O	-0.196965	1.804209	-0.000000
Si	-0.011564	-1.223825	-0.000000
H	0.982370	-1.110760	1.137224
H	0.982370	-1.110760	-1.137224

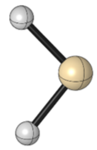

M06-2X/def2-svp
Thermal correction to Gibbs Energy = 0.043393
Thermal correction to Enthalpy = 0.077631
Sum of electronic and thermal Free Energies = −369.003632
Sum of electronic and thermal Enthalpies = −368.969394

Atomic Type	Coordinates (Angstroms) X	Y	Z

Si	0.000029	-0.763074	-0.000027
C	-1.428300	0.502408	-0.011865
H	-2.402575	0.073345	-0.281746
H	-1.507067	0.862890	1.032680
H	-1.228847	1.390668	-0.632790
C	1.428195	0.502477	0.011820
H	1.507662	0.863228	-1.032561
H	1.228907	1.390701	0.632905
H	2.402138	0.072894	0.282164

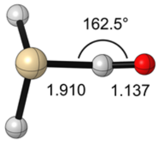

M06-2X/def2-svp
Thermal correction to Gibbs Energy = 0.049840
Thermal correction to Enthalpy = 0.089823
Sum of electronic and thermal Free Energies = −482.208922
Sum of electronic and thermal Enthalpies = −482.168939

Atomic Type	Coordinates (Angstroms) X	Y	Z
C	1.302943	0.000377	0.097223
O	2.435326	0.000665	0.199318
Si	-0.459326	-0.000074	-0.638259
C	-1.149041	1.529017	0.295178
H	-0.552384	2.429711	0.091182
H	-2.157561	1.720981	-0.102009
H	-1.233746	1.389895	1.381899
C	-1.148194	-1.529610	0.295077
H	-1.232981	-1.390607	1.381806
H	-2.156607	-1.722106	-0.102128
H	-0.551037	-2.429960	0.091023

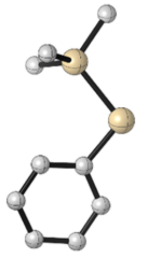

M06-2X/def2-svp
Thermal correction to Gibbs Energy = 0.161048
Thermal correction to Enthalpy = 0.218498
Sum of electronic and thermal Free Energies = -929.542072
Sum of electronic and thermal Enthalpies = -929.484623

Atomic Type	Coordinates (Angstroms) X	Y	Z
Si	-0.351859	-1.589556	-0.000827
Si	-2.068078	0.107162	0.000045
C	1.237552	-0.546049	-0.000415
C	2.445945	-1.274857	0.000039
C	1.336271	0.861495	-0.000508
C	3.686602	-0.639353	0.000413
H	2.404901	-2.367854	0.000083
C	2.570567	1.505008	-0.000212
H	0.430184	1.469972	-0.000848
C	3.747715	0.753358	0.000270
H	4.605003	-1.228509	0.000790
H	2.618780	2.595157	-0.000336
H	4.715886	1.257506	0.000540
C	-3.728187	-0.801529	0.000333
H	-3.825966	-1.444714	0.887980
H	-4.567663	-0.088866	0.001394
H	-3.827082	-1.443385	-0.888150
C	-2.022483	1.194569	1.554827
H	-2.119489	0.577663	2.460986
H	-1.095907	1.778555	1.645591
H	-2.870592	1.897509	1.538222
C	-2.023720	1.195962	-1.553788
H	-2.872233	1.898389	-1.536165
H	-1.097548	1.780615	-1.644389
H	-2.120722	0.579865	-2.460494

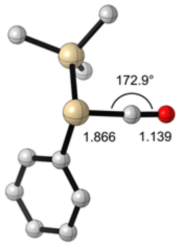

M06-2X/def2-svp
Thermal correction to Gibbs Energy = 0.166485
Thermal correction to Enthalpy = 0.229279
Sum of electronic and thermal Free Energies = −1042.764902
Sum of electronic and thermal Enthalpies = −1042.702108

Atomic Type	Coordinates (Angstroms) X	Y	Z
Si	0.364399	0.660939	-1.221134
Si	1.967872	-0.566771	0.078610
C	-1.338732	0.144054	-0.490182

C	-2.388788	1.062575	-0.332753
C	-1.600550	-1.203886	-0.198797
C	-3.640295	0.657381	0.128789
H	-2.232561	2.117078	-0.578939
C	-2.855373	-1.616943	0.249360
H	-0.812910	-1.952886	-0.322782
C	-3.878095	-0.685455	0.421133
H	-4.436464	1.393565	0.253027
H	-3.032974	-2.671523	0.467490
H	-4.859044	-1.005495	0.775526
C	3.578950	0.419082	0.069756
H	3.470711	1.381480	0.592390
H	4.370171	-0.151210	0.581073
H	3.914746	0.621822	-0.957784
C	1.426328	-0.871225	1.859880
H	1.355495	0.080200	2.408286
H	0.441869	-1.359185	1.904372
H	2.154324	-1.513427	2.380409
C	2.261843	-2.216669	-0.793464
H	3.043965	-2.783053	-0.263760
H	1.356628	-2.840681	-0.821635
H	2.595340	-2.054176	-1.828792
C	0.627817	2.010114	0.040994
O	0.857535	2.903122	0.709769

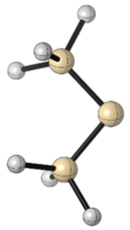

M06-2X/def2-svp
Thermal correction to Gibbs Energy = 0.178761
Thermal correction to Enthalpy = 0.241837
Sum of electronic and thermal Free Energies = −1107.137273
Sum of electronic and thermal Enthalpies = −1107.074198

Atomic Type	Coordinates (Angstroms) X	Y	Z
Si	0.000012	-1.600893	-0.000667
Si	-1.785355	-0.012063	0.007604
C	-3.397004	-0.861594	0.515772
H	-3.353787	-1.200723	1.561740
H	-4.251838	-0.174073	0.416677
H	-3.588202	-1.743406	-0.113569
C	-1.591671	1.520545	1.105025
H	-1.519218	1.237042	2.166070
H	-0.704418	2.116966	0.850575

H	-2.479538	2.163127	0.992969
C	-1.958034	0.520289	-1.808028
H	-2.833967	1.180987	-1.908730
H	-1.075025	1.067184	-2.167323
H	-2.120251	-0.346592	-2.466318
Si	1.785344	-0.012044	-0.007671
C	1.958145	0.518312	1.808541
H	2.834064	1.178930	1.909901
H	1.075147	1.064785	2.168506
H	2.120444	-0.349285	2.465868
C	3.396948	-0.861043	-0.516869
H	3.588170	-1.743537	0.111509
H	3.353663	-1.199037	-1.563201
H	4.251796	-0.173640	-0.417081
C	1.591611	1.521773	-1.103385
H	2.479466	2.164249	-0.990636
H	1.519152	1.239430	-2.164740
H	0.704347	2.117898	-0.848278

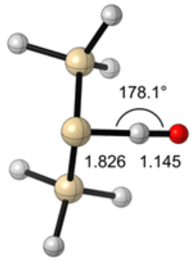

M06-2X/def2-svp
Thermal correction to Gibbs Energy = 0.186863
Thermal correction to Enthalpy = 0.253190
Sum of electronic and thermal Free Energies = −1220.376802
Sum of electronic and thermal Enthalpies = −1220.310475

Atomic Type	Coordinates (Angstroms) X	Y	Z
Si	-0.009110	0.334275	-1.391527
Si	1.818632	-0.356678	0.015573
C	3.327983	0.668321	-0.466643
H	3.173041	1.733167	-0.235696
H	4.216791	0.327905	0.087632
H	3.536618	0.579837	-1.542687
C	1.553866	-0.171023	1.877537
H	1.353738	0.878730	2.142233
H	0.715392	-0.783169	2.240846
H	2.461363	-0.487805	2.415836
C	2.129028	-2.171010	-0.404349
H	2.987820	-2.550364	0.171435
H	1.256268	-2.797372	-0.170407
H	2.354908	-2.284827	-1.474737

Si	-1.861886	-0.287881	0.013961
C	-2.098824	0.742010	1.579225
H	-2.959520	0.357667	2.149202
H	-1.213519	0.709413	2.230246
H	-2.300809	1.795553	1.334154
C	-3.421176	-0.134446	-1.037977
H	-3.578601	0.907520	-1.353822
H	-3.344930	-0.752853	-1.944280
H	-4.306917	-0.458782	-0.469192
C	-1.618703	-2.103408	0.480568
H	-2.516774	-2.478113	0.996654
H	-1.458766	-2.722403	-0.415142
H	-0.759126	-2.244080	1.152300
C	0.086151	1.850665	-0.378576
O	0.170770	2.815913	0.231827

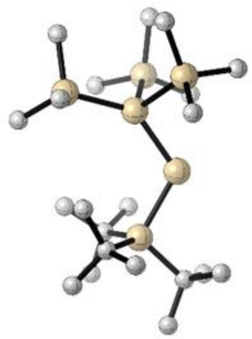

M06-2X/def2-svp
Thermal correction to Gibbs Energy = 0.634087
Thermal correction to Enthalpy = 0.754588
Sum of electronic and thermal Free Energies = −2567.459468
Sum of electronic and thermal Enthalpies = −2567.338967

Atomic Type	Coordinates (Angstroms) X	Y	Z
Si	-0.456058	-0.886118	1.089956
Si	-2.471987	-0.032335	0.085065
Si	1.511634	-0.038713	0.092951
Si	2.206011	1.670107	-1.406145
Si	2.556087	-2.044200	-0.626512
Si	2.554544	0.438018	2.170769
C	-2.637354	1.905041	-0.024487
C	-3.693623	2.326218	-1.058595
C	-1.283476	2.517093	-0.405558
C	-3.020586	2.520458	1.330078
C	-2.399062	-0.889948	-1.673418
C	-3.776037	-1.060663	-2.329117
C	-1.499116	-0.094861	-2.628127
C	-1.735539	-2.272191	-1.524232

C	-3.891177	-0.798127	1.191027
C	-5.286591	-0.277901	0.812580
C	-3.898769	-2.330837	1.073526
C	-3.634315	-0.486918	2.676692
C	2.106628	3.390393	-0.623000
C	4.040008	1.325930	-1.746977
C	1.389657	1.734257	-3.113025
C	2.285777	-2.211830	-2.494007
C	1.820318	-3.559714	0.231592
C	4.416805	-2.042545	-0.282480
C	4.278943	1.191950	1.974703
C	1.454823	1.671759	3.099849
C	2.709855	-1.125982	3.220182
H	-3.798995	3.425485	-1.054005
H	-4.683381	1.898886	-0.840221
H	-3.417920	2.030747	-2.080732
H	-1.392635	3.608146	-0.542655
H	-0.868429	2.100434	-1.332771
H	-0.538289	2.351387	0.388539
H	-4.035027	2.246628	1.649765
H	-2.989310	3.620766	1.247108
H	-2.318144	2.232526	2.128545
H	-4.292957	-0.097907	-2.458063
H	-4.435690	-1.722765	-1.751194
H	-3.657417	-1.509635	-3.331279
H	-1.904337	0.896694	-2.874747
H	-1.390196	-0.648432	-3.577926
H	-0.490865	0.041270	-2.208306
H	-2.270649	-2.949989	-0.847516
H	-0.691335	-2.178608	-1.166908
H	-1.671901	-2.766014	-2.510450
H	-5.392046	0.800782	0.997168
H	-6.051750	-0.787857	1.424073
H	-5.531407	-0.463810	-0.243686
H	-4.184760	-2.679273	0.071473
H	-4.634256	-2.745379	1.784602
H	-2.918481	-2.765810	1.328237
H	-4.414972	-0.970389	3.289799
H	-3.659504	0.585569	2.904255
H	-2.664415	-0.888143	3.012694
H	1.071568	3.741244	-0.515191
H	2.643414	4.108034	-1.263780
H	2.578927	3.408676	0.370665
H	4.456891	2.151672	-2.345944
H	4.173650	0.396243	-2.320617
H	4.628070	1.244948	-0.821824
H	1.978329	2.422816	-3.740377
H	0.355524	2.103168	-3.089805
H	1.390652	0.748140	-3.600592
H	2.777290	-1.395128	-3.044397
H	1.214101	-2.190580	-2.745973

H	2.702789	-3.163828	-2.859121
H	0.760246	-3.705560	-0.025560
H	1.884931	-3.466866	1.325710
H	2.372219	-4.462948	-0.074579
H	4.619371	-2.017069	0.798736
H	4.920789	-1.182103	-0.744246
H	4.868335	-2.962392	-0.687498
H	4.259392	2.132076	1.404060
H	4.959135	0.494368	1.463118
H	4.699391	1.408705	2.969760
H	0.462172	1.232955	3.298230
H	1.306524	2.594691	2.518331
H	1.896446	1.946598	4.070813
H	1.729610	-1.606124	3.357462
H	3.115938	-0.872637	4.212466
H	3.387090	-1.858512	2.755929

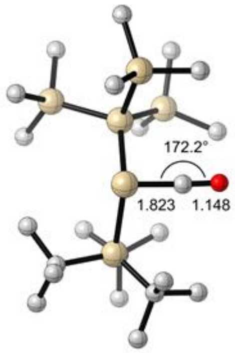

M06-2X/def2-svp
Thermal correction to Gibbs Energy = 0.641088
Thermal correction to Enthalpy = 0.766420
Sum of electronic and thermal Free Energies = −2680.691502
Sum of electronic and thermal Enthalpies = −2680.566170

Atomic Type	Coordinates (Angstroms) X	Y	Z
Si	0.439886	-0.377066	-1.106775
Si	2.566557	0.064348	-0.083144
Si	-1.686346	0.021541	-0.076435
Si	-2.301622	2.297983	0.090411
Si	-2.320550	-1.051483	1.931366
Si	-3.076693	-0.854048	-1.788956
O	0.300926	-3.290686	-0.580639
C	0.368129	-2.150345	-0.689569
C	3.014723	1.759555	-0.952976
C	4.188589	2.478336	-0.267406
C	1.807715	2.711147	-0.936840
C	3.366449	1.536847	-2.432452
C	2.449756	0.239591	1.851563

C	3.830951	0.322141	2.521403
C	1.649853	1.497849	2.215214
C	1.678769	-0.946620	2.447099
C	3.809018	-1.351167	-0.606821
C	5.262618	-0.848610	-0.542672
C	3.710765	-2.577496	0.316160
C	3.528840	-1.849472	-2.036056
C	-2.333217	3.085516	-1.627557
C	-4.053359	2.337269	0.811975
C	-1.273227	3.379154	1.251042
C	-1.857617	-0.018027	3.447169
C	-1.605435	-2.794908	2.080565
C	-4.205892	-1.240564	1.922358
C	-4.861776	-0.230578	-1.680751
C	-2.408212	-0.380547	-3.491560
C	-3.109564	-2.743727	-1.671442
H	4.444462	3.388792	-0.836735
H	5.094358	1.858011	-0.213545
H	3.934285	2.796659	0.754048
H	2.075495	3.651065	-1.449853
H	1.490005	2.968968	0.079472
H	0.939888	2.291110	-1.472681
H	4.296364	0.969361	-2.569886
H	3.506530	2.516574	-2.920819
H	2.559405	1.012600	-2.968711
H	4.446592	1.141196	2.122751
H	4.400389	-0.611832	2.414260
H	3.702673	0.501276	3.603133
H	2.180425	2.426580	1.961102
H	1.466797	1.515611	3.304235
H	0.667507	1.510578	1.715069
H	2.151438	-1.918017	2.253425
H	0.656643	-0.981983	2.044899
H	1.605419	-0.825261	3.542570
H	5.469922	-0.065298	-1.284024
H	5.945971	-1.688561	-0.757209
H	5.527436	-0.455061	0.449908
H	4.038361	-2.359081	1.341729
H	4.372318	-3.369751	-0.074441
H	2.695609	-2.996964	0.363101
H	4.312846	-2.570480	-2.325989
H	3.521925	-1.045690	-2.782581
H	2.565067	-2.375124	-2.106706
H	-1.353013	3.024185	-2.123764
H	-2.612989	4.148231	-1.554016
H	-3.070590	2.584826	-2.273327
H	-4.437602	3.369268	0.771583
H	-4.044917	2.027223	1.868342
H	-4.754780	1.689088	0.269427
H	-1.787655	4.347752	1.357795
H	-0.262439	3.576238	0.872335

H	-1.186763	2.930079	2.251568
H	-2.381935	0.949972	3.427800
H	-0.778030	0.180946	3.500839
H	-2.155589	-0.543520	4.368384
H	-0.507886	-2.811373	2.126859
H	-1.914290	-3.417351	1.227336
H	-1.991246	-3.264095	2.999805
H	-4.535144	-1.909839	1.113056
H	-4.719351	-0.276110	1.799153
H	-4.534465	-1.682014	2.876958
H	-4.920558	0.851548	-1.872060
H	-5.316641	-0.432578	-0.700174
H	-5.466797	-0.739228	-2.448258
H	-1.425599	-0.840688	-3.670121
H	-2.289360	0.708718	-3.591450
H	-3.102810	-0.724250	-4.274538
H	-2.103146	-3.176944	-1.769438
H	-3.732124	-3.155510	-2.481749
H	-3.534328	-3.084270	-0.714974

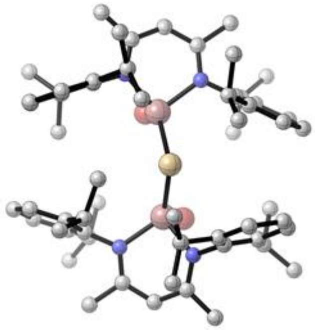

M06-2X/def2-svp
Thermal correction to Gibbs Energy = 1.175466
Thermal correction to Enthalpy = 1.362716
Sum of electronic and thermal Free Energies = −11761.131548
Sum of electronic and thermal Enthalpies = −11760.944297

Atomic Type	Coordinates (Angstroms) X	Y	Z
Br	-2.470214	-0.204275	-2.530337
Ga	-2.006679	0.308056	-0.225205
Si	-0.018049	-0.452038	1.015111
N	-3.513947	-0.592925	0.743699
N	-2.788978	2.131104	-0.041497
C	-4.740436	-0.093611	0.698520
C	-5.020612	1.227016	0.299175
H	-6.070183	1.509345	0.307394
C	-4.109720	2.277969	0.088667
C	-5.903317	-0.955271	1.122210
H	-5.720007	-1.408529	2.106174

H	-6.017379	-1.783378	0.407488
H	-6.833497	-0.378140	1.151746
C	-4.696846	3.669006	0.056201
H	-5.773516	3.639134	0.250175
H	-4.518198	4.144192	-0.917158
H	-4.208986	4.303784	0.810074
C	-3.260427	-1.840765	1.402126
C	-3.396073	-3.062231	0.715010
C	-3.096625	-4.247517	1.399074
H	-3.189566	-5.200558	0.873577
C	-2.680415	-4.230604	2.722876
H	-2.453985	-5.164819	3.239782
C	-2.539422	-3.014170	3.387503
H	-2.192685	-3.008542	4.421365
C	-2.813578	-1.805184	2.745790
C	-3.808814	-3.136318	-0.744537
H	-4.081371	-2.125170	-1.078343
C	-2.626701	-3.598390	-1.602357
H	-2.382825	-4.650568	-1.387198
H	-1.723927	-2.999111	-1.408550
H	-2.864985	-3.502226	-2.672292
C	-5.012370	-4.059169	-0.965100
H	-5.872738	-3.778230	-0.340337
H	-4.761666	-5.104870	-0.730611
H	-5.326812	-4.023381	-2.018279
C	-2.633939	-0.482633	3.476292
H	-2.296427	0.242620	2.720639
C	-3.951662	0.043379	4.057904
H	-4.692931	0.253587	3.275104
H	-3.777363	0.980631	4.608716
H	-4.384108	-0.688075	4.758385
C	-1.985926	3.319855	-0.139524
C	-1.923336	4.046721	-1.348473
C	-1.258065	5.277008	-1.347444
H	-1.215952	5.856638	-2.271541
C	-0.642546	5.769758	-0.202773
H	-0.137044	6.736758	-0.225062
C	-0.637582	5.004661	0.958055
H	-0.114042	5.368434	1.843886
C	-1.303985	3.777206	1.008507
C	-1.229392	2.925139	2.261843
H	-2.069402	2.216338	2.234672
C	-1.342513	3.720215	3.563948
H	-2.241342	4.353056	3.565208
H	-0.466613	4.366185	3.724161
H	-1.402568	3.033952	4.421433
C	0.083794	2.134476	2.248052
H	0.151278	1.428429	3.087172
H	0.953414	2.809326	2.282426
H	0.206691	1.616676	1.258095
C	-2.493435	3.512339	-2.651847

H	-3.136477	2.650247	-2.422727
C	-3.326153	4.544231	-3.418918
H	-3.788474	4.069922	-4.296356
H	-2.702873	5.372629	-3.787828
H	-4.129508	4.978676	-2.805632
C	-1.342945	3.003011	-3.530345
H	-1.737595	2.522357	-4.437739
H	-0.724179	2.260427	-3.006118
H	-0.694612	3.841929	-3.832122
Br	1.749870	0.434079	-2.871485
Ga	1.864106	-0.251043	-0.559725
N	3.539136	0.599484	0.084014
N	2.710048	-2.059291	-0.698412
C	4.718249	0.150709	-0.348960
C	4.908941	-1.100923	-0.951997
H	5.920056	-1.321288	-1.285244
C	3.995539	-2.177134	-0.996641
C	5.934297	1.019772	-0.150089
H	6.108316	1.201630	0.919572
H	5.767425	2.003198	-0.612260
H	6.825188	0.556908	-0.586683
C	4.545659	-3.524106	-1.393839
H	5.640589	-3.521173	-1.372118
H	4.213197	-3.764693	-2.414062
H	4.165478	-4.317480	-0.736373
C	3.491546	1.744166	0.943123
C	3.314424	3.034796	0.401418
C	3.229078	4.121434	1.278997
H	3.079287	5.122999	0.869678
C	3.345305	3.949182	2.654857
H	3.287991	4.809993	3.323281
C	3.543189	2.673249	3.173415
H	3.645288	2.540369	4.252957
C	3.613625	1.553676	2.337695
C	3.218192	3.269037	-1.095135
H	3.438533	2.320979	-1.603858
C	1.798693	3.687470	-1.485082
H	1.576251	4.691185	-1.095414
H	1.037903	2.996683	-1.084161
H	1.685493	3.700180	-2.579236
C	4.229964	4.311788	-1.582229
H	5.260788	4.050344	-1.300809
H	4.013425	5.307977	-1.167272
H	4.185219	4.393153	-2.677880
C	3.857690	0.184248	2.955452
H	3.857761	-0.561532	2.146043
C	2.748007	-0.195520	3.939977
H	2.650037	0.554064	4.740631
H	2.979014	-1.160375	4.415660
H	1.775512	-0.291432	3.431292
C	5.225435	0.123986	3.646517

H	6.042435	0.381878	2.958183
H	5.414700	-0.888453	4.032966
H	5.268968	0.822324	4.496463
C	1.866115	-3.217066	-0.619803
C	1.351174	-3.812501	-1.791891
C	0.539382	-4.943820	-1.657307
H	0.134727	-5.414014	-2.556111
C	0.213808	-5.456797	-0.409505
H	-0.433559	-6.331803	-0.326977
C	0.701316	-4.841471	0.738350
H	0.425553	-5.242382	1.713056
C	1.543133	-3.726891	0.662129
C	2.140402	-3.134902	1.933374
H	2.192465	-2.044109	1.797139
C	3.574332	-3.626292	2.167051
H	4.253875	-3.307886	1.363750
H	3.605017	-4.725182	2.233406
H	3.964569	-3.216761	3.112313
C	1.273332	-3.398735	3.164006
H	1.649878	-2.825065	4.021746
H	1.291315	-4.461049	3.452778
H	0.228619	-3.102627	2.983404
C	1.596920	-3.259124	-3.186539
H	2.328389	-2.441160	-3.114676
C	2.143562	-4.321507	-4.147904
H	2.411979	-3.856689	-5.107599
H	1.387606	-5.093008	-4.358418
H	3.032888	-4.832837	-3.751580
C	0.301745	-2.663255	-3.749707
H	0.491565	-2.182466	-4.720903
H	-0.121712	-1.898850	-3.084120
H	-0.454905	-3.451733	-3.893599
C	-1.557313	-0.539565	4.560028
H	-1.347832	0.474862	4.932757
H	-0.622508	-0.967076	4.165934
H	-1.879807	-1.138132	5.425767

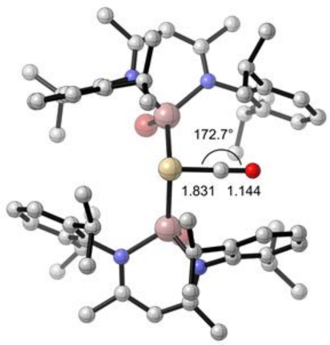

M06-2X/def2-svp

Thermal correction to Gibbs Energy = 1.182129

Thermal correction to Enthalpy = 1.374975
Sum of electronic and thermal Free Energies = −11874.375967
Sum of electronic and thermal Enthalpies = −11874.183120

Atomic Type	Coordinates (Angstroms) X	Y	Z
Br	1.721165	0.912632	-2.773029
Ga	2.001967	0.135122	-0.521870
Si	0.024219	-0.097075	0.905094
O	0.118930	-3.061162	0.764447
C	0.077599	-1.918697	0.729072
N	3.292216	1.440117	0.257780
N	3.355235	-1.301132	-0.667576
C	4.571439	1.390171	-0.101992
C	5.154783	0.305361	-0.785451
H	6.194411	0.429015	-1.077312
C	4.619816	-0.982516	-0.940840
C	5.483746	2.536789	0.256805
H	5.418150	2.762962	1.330322
H	5.171320	3.444962	-0.276273
H	6.521303	2.305642	-0.004923
C	5.553772	-2.071884	-1.402147
H	6.536305	-1.663038	-1.659050
H	5.126943	-2.584839	-2.275555
H	5.671860	-2.833167	-0.618439
C	2.847686	2.531557	1.077306
C	2.551641	3.782219	0.497441
C	2.179370	4.833819	1.342850
H	1.939077	5.805699	0.906757
C	2.118799	4.663972	2.719716
H	1.842763	5.499753	3.364933
C	2.389754	3.416199	3.274127
H	2.308488	3.285427	4.353309
C	2.735357	2.325823	2.470981
C	2.586458	4.014593	-1.003515
H	3.043757	3.136990	-1.481058
C	1.157946	4.141492	-1.541462
H	0.706412	5.082803	-1.195439
H	0.513614	3.316154	-1.199752
H	1.156580	4.131804	-2.641663
C	3.398456	5.253450	-1.397663
H	4.422831	5.232657	-0.998167
H	2.921292	6.176720	-1.036130
H	3.461587	5.324871	-2.493101
C	2.999375	0.961945	3.094274
H	2.659192	0.205292	2.368994
C	4.493746	0.721569	3.339162
H	5.067175	0.708407	2.400837
H	4.647629	-0.249706	3.834898
H	4.910820	1.505699	3.990537

C	2.995708	-2.686686	-0.564695
C	2.481391	-3.373136	-1.683430
C	2.079268	-4.702085	-1.516766
H	1.657863	-5.239712	-2.368901
C	2.217852	-5.350573	-0.294848
H	1.900912	-6.388925	-0.186238
C	2.767878	-4.671882	0.786364
H	2.882027	-5.186272	1.743190
C	3.157031	-3.334376	0.678312
C	3.726984	-2.630846	1.901865
H	3.956248	-1.589732	1.627551
C	5.032297	-3.287613	2.366138
H	5.781147	-3.317930	1.562113
H	4.856529	-4.321608	2.700186
H	5.463733	-2.730414	3.211153
C	2.712007	-2.599288	3.049838
H	3.166484	-2.147432	3.944323
H	2.383689	-3.614781	3.318289
H	1.816539	-2.013352	2.791061
C	2.361350	-2.719665	-3.049413
H	2.861769	-1.741988	-3.005060
C	3.045050	-3.547659	-4.142620
H	3.019355	-3.002359	-5.097114
H	2.533223	-4.509386	-4.297899
H	4.095994	-3.764977	-3.899729
C	0.894968	-2.466313	-3.403011
H	0.812248	-1.895105	-4.339397
H	0.383773	-1.879472	-2.626067
H	0.353727	-3.418352	-3.523112
Br	-2.278795	-0.618105	-2.684063
Ga	-2.095149	-0.112698	-0.350989
N	-3.441917	-1.315642	0.480250
N	-3.212185	1.520118	-0.166466
C	-4.738217	-1.056055	0.339494
C	-5.240504	0.192864	-0.062615
H	-6.321475	0.261516	-0.152944
C	-4.539720	1.409476	-0.172940
C	-5.742192	-2.143782	0.630258
H	-5.515214	-2.661181	1.571558
H	-5.690873	-2.898180	-0.169498
H	-6.759117	-1.739326	0.668623
C	-5.381064	2.658653	-0.275018
H	-6.439614	2.425743	-0.121550
H	-5.255265	3.118035	-1.264718
H	-5.059201	3.405832	0.463733
C	-3.014390	-2.502548	1.161999
C	-2.889925	-3.728085	0.480620
C	-2.423417	-4.837313	1.194380
H	-2.311837	-5.792471	0.677243
C	-2.075463	-4.739693	2.535001
H	-1.699762	-5.614002	3.068629

C	-2.203420	-3.521336	3.195718
H	-1.933140	-3.452294	4.250158
C	-2.680810	-2.388948	2.531722
C	-3.180464	-3.876006	-1.002819
H	-3.625906	-2.937366	-1.364374
C	-1.873213	-4.081486	-1.774613
H	-1.385606	-5.023297	-1.474635
H	-1.166447	-3.262161	-1.588992
H	-2.067250	-4.112355	-2.857477
C	-4.157424	-5.020438	-1.291891
H	-5.086332	-4.928926	-0.710040
H	-3.710891	-5.996797	-1.050204
H	-4.418935	-5.033691	-2.359938
C	-2.906430	-1.090574	3.291372
H	-2.845033	-0.267564	2.565065
C	-1.860575	-0.831976	4.374862
H	-1.943672	-1.550266	5.204544
H	-2.010803	0.170597	4.803006
H	-0.840692	-0.882730	3.964436
C	-4.320257	-1.060090	3.887656
H	-5.091182	-1.085818	3.104702
H	-4.472489	-0.142869	4.475762
H	-4.476131	-1.924122	4.552395
C	-2.634569	2.836452	-0.159047
C	-2.586387	3.606655	-1.341538
C	-2.129442	4.924752	-1.250057
H	-2.096204	5.538244	-2.152584
C	-1.695199	5.458958	-0.043013
H	-1.338617	6.489649	0.003134
C	-1.684166	4.666548	1.099150
H	-1.309211	5.080029	2.035721
C	-2.160425	3.351817	1.066848
C	-2.219200	2.524644	2.340985
H	-2.064409	1.476481	2.052571
C	-3.593712	2.610692	3.013035
H	-4.387247	2.185206	2.380923
H	-3.851364	3.657427	3.238822
H	-3.587050	2.049623	3.960573
C	-1.102666	2.872194	3.321029
H	-1.082801	2.139889	4.141528
H	-1.241514	3.866392	3.773961
H	-0.123507	2.855485	2.821047
C	-2.957703	3.047780	-2.705883
H	-3.463715	2.081885	-2.561729
C	-3.890993	3.968657	-3.499528
H	-4.212900	3.466287	-4.422994
H	-3.380928	4.897530	-3.795601
H	-4.790558	4.251432	-2.933259
C	-1.681528	2.778179	-3.512452
H	-1.926607	2.288311	-4.466613
H	-0.986713	2.120311	-2.972201

H	-1.160744	3.725168	-3.728813
C	2.196666	0.747505	4.377598
H	2.272216	-0.299029	4.701957
H	1.133367	0.980651	4.218907
H	2.572044	1.371519	5.202863

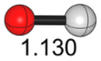

B3LYP-D3(BJ)/def2-svp
Thermal correction to Gibbs Energy = −0.014012
Thermal correction to Enthalpy = 0.008418
Sum of electronic and thermal Free Energies = −113.239753
Sum of electronic and thermal Enthalpies = −113.217322

Atomic Type	Coordinates (Angstroms) X	Y	Z
C	0.000000	0.000000	-0.645938
O	0.000000	0.000000	0.484454

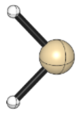

B3LYP-D3(BJ)/def2-svp
Thermal cortion to Gibbs Energy = −0.008186
Thermal correction to Enthalpy = 0.015379
Sum of electronic and thermal Free Energies = −290.548369
Sum of electronic and thermal Enthalpies = −290.524804

Atomic Type	Coordinates (Angstroms) X	Y	Z
Si	0.000000	0.000000	0.133924
H	0.000000	1.098102	-0.937468
H	-0.000000	-1.098102	-0.937468

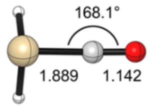

B3LYP-D3(BJ)/def2-svp
Thermal correction to Gibbs Energy = −0.003642
Thermal correction to Enthalpy = 0.027234
Sum of electronic and thermal Free Energies = −403.819247
Sum of electronic and thermal Enthalpies = −403.788371

Atomic Type	Coordinates (Angstroms) X	Y	Z

C	-0.000223	0.707832	-0.000000
O	-0.237719	1.824634	-0.000000
Si	0.003973	-1.180697	0.000000
H	0.985761	-1.151649	1.157568
H	0.985761	-1.151649	-1.157568

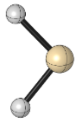

B3LYP-D3(BJ)/def2-svp
Thermal correction to Gibbs Energy = 0.042599
Thermal correction to Enthalpy = 0.077015
Sum of electronic and thermal Free Energies = −369.105858
Sum of electronic and thermal Enthalpies = −369.071443

Atomic Type	Coordinates (Angstroms) X	Y	Z
Si	0.000016	-0.761698	0.000007
C	-1.440448	0.501596	-0.011389
H	-2.412626	0.061975	-0.283146
H	-1.529017	0.868738	1.032472
H	-1.250576	1.391514	-0.637337
C	1.440444	0.501605	0.011390
H	1.528708	0.868705	-1.032509
H	1.250620	1.391529	0.637354
H	2.412697	0.062112	0.283064

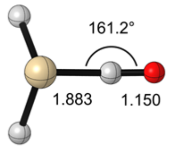

B3LYP-D3(BJ)/def2-svp
Thermal correction to Gibbs Energy = 0.049257
Thermal correction to Enthalpy = 0.089137
Sum of electronic and thermal Free Energies = −482.359511
Sum of electronic and thermal Enthalpies = −482.319631

Atomic Type	Coordinates (Angstroms) X	Y	Z
C	-1.325377	-0.000039	0.100115
O	-2.473888	-0.000035	0.151671
Si	0.427874	-0.000001	-0.587219
C	1.151912	-1.541931	0.303663
H	0.554501	-2.445019	0.102197

H	2.156796	-1.722632	-0.113255
H	1.254309	-1.414787	1.392620
C	1.151806	1.541985	0.303656
H	1.254222	1.414854	1.392612
H	2.156672	1.722762	-0.113272
H	0.554324	2.445027	0.102192

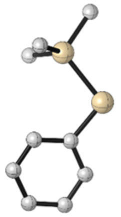

B3LYP-D3(BJ)/def2-svp
Thermal correction to Gibbs Energy = 0.159281
Thermal correction to Enthalpy = 0.217047
Sum of electronic and thermal Free Energies = −929.846457
Sum of electronic and thermal Enthalpies = −929.788691

Atomic Type	Coordinates (Angstroms) X	Y	Z
Si	-0.350232	-1.585722	0.001728
Si	-2.079360	0.105234	0.000027
C	1.241029	-0.545098	0.000956
C	2.452547	-1.278871	0.000245
C	1.344921	0.866380	0.000822
C	3.696285	-0.645127	-0.000438
H	2.407166	-2.372287	0.000304
C	2.583072	1.507117	0.000173
H	0.441265	1.476397	0.001259
C	3.761428	0.751051	-0.000466
H	4.614983	-1.236951	-0.000972
H	2.634425	2.598831	0.000138
H	4.732207	1.253509	-0.001026
C	-3.741313	-0.808623	0.000376
H	-3.840618	-1.452134	0.889977
H	-4.585801	-0.098630	-0.001093
H	-3.839590	-1.454587	-0.887559
C	-2.032763	1.201483	1.554345
H	-2.101940	0.588215	2.467828
H	-1.111668	1.799984	1.624451
H	-2.891645	1.894689	1.552998
C	-2.030103	1.196093	-1.558044
H	-2.890583	1.887298	-1.562263
H	-1.110106	1.796462	-1.626714
H	-2.094419	0.579602	-2.469715

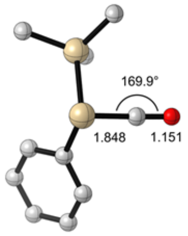

B3LYP-D3(BJ)/def2-svp
Thermal correction to Gibbs Energy = 0.165141
Thermal correction to Enthalpy = 0.227844
Sum of electronic and thermal Free Energies = −1043.116231
Sum of electronic and thermal Enthalpies = −1043.053528

Atomic Type	Coordinates (Angstroms) X	Y	Z
Si	0.367090	0.675125	-1.139910
Si	1.982538	-0.602344	0.089172
C	-1.350821	0.154881	-0.461595
C	-2.391470	1.081202	-0.259564
C	-1.632088	-1.206331	-0.240117
C	-3.652597	0.668058	0.175181
H	-2.219038	2.145576	-0.446379
C	-2.896851	-1.624890	0.182581
H	-0.855357	-1.958917	-0.400280
C	-3.910544	-0.687966	0.398187
H	-4.440480	1.408693	0.335247
H	-3.089172	-2.688380	0.346473
H	-4.898678	-1.012771	0.732670
C	3.611387	0.358591	0.097350
H	3.519479	1.308843	0.647481
H	4.401086	-0.236681	0.586213
H	3.944668	0.588428	-0.927159
C	1.434107	-0.960367	1.861174
H	1.374585	-0.027880	2.444633
H	0.440137	-1.432955	1.883439
H	2.148247	-1.635675	2.363038
C	2.228727	-2.232992	-0.840569
H	3.002243	-2.839988	-0.339861
H	1.304224	-2.831223	-0.874197
H	2.552293	-2.051397	-1.877713
C	0.656383	2.084375	0.019979
O	0.917946	3.054754	0.580137

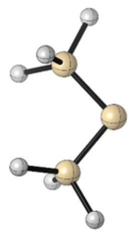

B3LYP-D3(BJ)/def2-svp
Thermal correction to Gibbs Energy = 0.177702
Thermal correction to Enthalpy = 0.240515
Sum of electronic and thermal Free Energies = −1107.446568
Sum of electronic and thermal Enthalpies = −1107.383756

Atomic Type	Coordinates (Angstroms) X	Y	Z
Si	-0.000001	-1.593780	0.000203
Si	-1.798292	-0.013008	0.006848
C	-3.406368	-0.874195	0.522951
H	-3.357800	-1.214732	1.570364
H	-4.268641	-0.191781	0.429499
H	-3.598202	-1.758914	-0.105286
C	-1.607771	1.525189	1.103502
H	-1.492815	1.245283	2.163197
H	-0.740958	2.139657	0.819888
H	-2.512137	2.152412	1.022146
C	-1.978499	0.523858	-1.811502
H	-2.869647	1.166756	-1.915509
H	-1.104863	1.092816	-2.162632
H	-2.116612	-0.342347	-2.478936
Si	1.798300	-0.013013	-0.006826
C	1.978433	0.524516	1.811333
H	2.869519	1.167533	1.915129
H	1.104740	1.093515	2.162255
H	2.116617	-0.341445	2.479068
C	3.406408	-0.874365	-0.522553
H	3.598216	-1.758873	0.105989
H	3.357893	-1.215251	-1.569855
H	4.268673	-0.191915	-0.429285
C	1.607789	1.524774	-1.104056
H	2.512169	2.152011	-1.022957
H	1.492797	1.244478	-2.163643
H	0.740996	2.139362	-0.820643

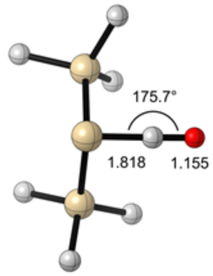

B3LYP-D3(BJ)/def2-svp
Thermal correction to Gibbs Energy = 0.184505
Thermal correction to Enthalpy = 0.251765
Sum of electronic and thermal Free Energies = −1220.734111
Sum of electronic and thermal Enthalpies = −1220.666851

Atomic Type	Coordinates (Angstroms) X	Y	Z
Si	-0.003957	0.418586	-1.293143
Si	1.855120	-0.363249	0.011399
C	3.350891	0.723083	-0.383814
H	3.185012	1.761959	-0.055499
H	4.250922	0.345636	0.130859
H	3.555056	0.738676	-1.466028
C	1.589517	-0.367899	1.884940
H	1.372897	0.646472	2.255832
H	0.754879	-1.022772	2.178378
H	2.497968	-0.730293	2.396038
C	2.177074	-2.133020	-0.576004
H	3.047601	-2.558097	-0.047926
H	1.310994	-2.785251	-0.383796
H	2.386788	-2.156016	-1.657150
Si	-1.886273	-0.314841	0.010484
C	-2.107128	0.537822	1.684137
H	-2.997174	0.137625	2.199346
H	-1.234972	0.382272	2.336370
H	-2.248286	1.623736	1.563117
C	-3.440681	-0.022500	-1.025392
H	-3.587946	1.051947	-1.220702
H	-3.369918	-0.533550	-1.998660
H	-4.335322	-0.399550	-0.500706
C	-1.657327	-2.176115	0.275852
H	-2.558061	-2.602538	0.749178
H	-1.496548	-2.698858	-0.680531
H	-0.798437	-2.391490	0.929832
C	0.059722	1.951935	-0.318139
O	0.115460	2.967913	0.227525

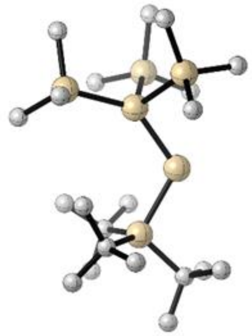

B3LYP-D3(BJ)/def2-svp
Thermal correction to Gibbs Energy = 0.629982
Thermal correction to Enthalpy = 0.751686
Sum of electronic and thermal Free Energies = −2568.328914
Sum of electronic and thermal Enthalpies = −2568.207210

Atomic Type	Coordinates (Angstroms) X	Y	Z
Si	-0.460329	-0.852899	1.073306
Si	-2.479438	-0.023754	0.067994
Si	1.519219	-0.041972	0.084975
Si	2.253488	1.631471	-1.440324
Si	2.558935	-2.075770	-0.567555
Si	2.525888	0.494921	2.170096
C	-2.646692	1.920058	-0.088696
C	-3.724851	2.317833	-1.114731
C	-1.297330	2.517703	-0.516019
C	-2.998142	2.564914	1.265199
C	-2.421564	-0.937456	-1.671558
C	-3.804882	-1.137580	-2.312483
C	-1.529426	-0.162062	-2.653505
C	-1.748426	-2.312955	-1.473439
C	-3.884324	-0.765941	1.224737
C	-5.290215	-0.252835	0.861589
C	-3.891318	-2.304854	1.143352
C	-3.587806	-0.413033	2.698167
C	2.165981	3.366007	-0.678446
C	4.089066	1.277366	-1.780551
C	1.426411	1.674139	-3.145232
C	2.289516	-2.291426	-2.433221
C	1.817226	-3.569605	0.328315
C	4.419913	-2.068921	-0.211741
C	4.261059	1.232427	1.979031
C	1.411260	1.773471	3.024198
C	2.656220	-1.035310	3.275659
H	-3.818461	3.418695	-1.154161
H	-4.715001	1.913919	-0.859081
H	-3.477402	1.978845	-2.130865
H	-1.386113	3.614524	-0.620166

H	-0.938197	2.123047	-1.472201
H	-0.518036	2.314667	0.232348
H	-4.000335	2.291212	1.621063
H	-2.978683	3.664846	1.162219
H	-2.271926	2.299968	2.050227
H	-4.319715	-0.181440	-2.488830
H	-4.465380	-1.765360	-1.698216
H	-3.697777	-1.638520	-3.292538
H	-1.944728	0.817976	-2.925160
H	-1.416224	-0.737456	-3.590143
H	-0.523546	-0.004506	-2.242567
H	-2.307270	-2.984904	-0.812028
H	-0.725297	-2.207766	-1.063689
H	-1.629363	-2.824496	-2.446255
H	-5.387226	0.833206	1.002586
H	-6.046075	-0.731356	1.511028
H	-5.563221	-0.480138	-0.179421
H	-4.195954	-2.676993	0.155497
H	-4.611310	-2.709271	1.877453
H	-2.905848	-2.733396	1.386519
H	-4.336081	-0.895642	3.352886
H	-3.626787	0.663993	2.900196
H	-2.597709	-0.782923	3.012387
H	1.132677	3.704654	-0.520849
H	2.659754	4.086371	-1.352781
H	2.686443	3.404424	0.290792
H	4.507468	2.095533	-2.391721
H	4.223334	0.340145	-2.342024
H	4.679745	1.208790	-0.856081
H	2.010142	2.347774	-3.795753
H	0.394847	2.050800	-3.114611
H	1.413009	0.679279	-3.615975
H	2.765791	-1.479988	-3.005589
H	1.216426	-2.291571	-2.681632
H	2.718948	-3.246856	-2.779770
H	0.755101	-3.716463	0.079059
H	1.886383	-3.453300	1.420277
H	2.363832	-4.484581	0.041264
H	4.613832	-2.005308	0.870282
H	4.931165	-1.226176	-0.698140
H	4.874521	-3.004521	-0.580266
H	4.257267	2.157885	1.384000
H	4.943798	0.517986	1.493838
H	4.672295	1.472589	2.974521
H	0.411389	1.348485	3.219261
H	1.277918	2.669440	2.396931
H	1.832181	2.093897	3.992430
H	1.673415	-1.514846	3.400387
H	3.037845	-0.754421	4.272128
H	3.345417	-1.780359	2.848317

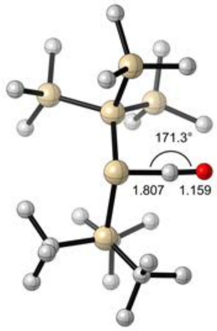

B3LYP-D3(BJ)/def2-svp
Thermal correction to Gibbs Energy = 0.637529
Thermal correction to Enthalpy = 0.763539
Sum of electronic and thermal Free Energies = −2681.609383
Sum of electronic and thermal Enthalpies = −2681.483373

Atomic Type	Coordinates (Angstroms) X	Y	Z
Si	0.434203	-0.420672	-1.041095
Si	2.575069	0.059829	-0.074057
Si	-1.710376	0.020369	-0.068847
Si	-2.290120	2.311643	0.021330
Si	-2.348098	-0.988621	1.970611
Si	-3.078254	-0.912844	-1.772619
O	0.280538	-3.336708	-0.572619
C	0.357768	-2.183160	-0.651150
C	2.992487	1.753108	-0.984258
C	4.167096	2.501995	-0.325104
C	1.764293	2.681946	-0.970547
C	3.329994	1.502408	-2.466449
C	2.478334	0.260471	1.868591
C	3.870195	0.373882	2.519838
C	1.661446	1.515428	2.219009
C	1.735001	-0.935824	2.489161
C	3.822566	-1.363493	-0.602353
C	5.277530	-0.849827	-0.566945
C	3.738170	-2.579325	0.341960
C	3.522551	-1.878805	-2.025998
C	-2.231979	3.062391	-1.715774
C	-4.067286	2.411489	0.678635
C	-1.274307	3.391151	1.199141
C	-1.855466	0.078253	3.457022
C	-1.646562	-2.736024	2.158351
C	-4.239163	-1.149504	1.984051
C	-4.863463	-0.276253	-1.711016
C	-2.376887	-0.490394	-3.478615
C	-3.129055	-2.799323	-1.589560
H	4.395372	3.416671	-0.901541
H	5.086275	1.901025	-0.288669
H	3.931337	2.819439	0.700866

H	1.991069	3.609759	-1.525248
H	1.464054	2.970721	0.041822
H	0.891868	2.222220	-1.462662
H	4.265242	0.943682	-2.602860
H	3.452160	2.471386	-2.982648
H	2.524824	0.955292	-2.981440
H	4.457670	1.212391	2.120362
H	4.464048	-0.542642	2.394946
H	3.758110	0.540216	3.606558
H	2.164900	2.446446	1.924569
H	1.502638	1.564885	3.310959
H	0.669502	1.497144	1.746342
H	2.250324	-1.891732	2.339787
H	0.726329	-1.032339	2.069483
H	1.631331	-0.784151	3.578533
H	5.464659	-0.064275	-1.311386
H	5.966629	-1.682556	-0.796062
H	5.561146	-0.454555	0.419104
H	4.078943	-2.345355	1.359689
H	4.394667	-3.380534	-0.041542
H	2.724591	-2.997702	0.409837
H	4.287418	-2.623044	-2.312314
H	3.533456	-1.086526	-2.783770
H	2.546469	-2.380405	-2.088826
H	-1.228479	2.977810	-2.161205
H	-2.500499	4.131823	-1.682408
H	-2.941533	2.555506	-2.388240
H	-4.419294	3.455545	0.616917
H	-4.107539	2.109768	1.737185
H	-4.769358	1.780701	0.117315
H	-1.765607	4.376319	1.275501
H	-0.246764	3.556479	0.851291
H	-1.230010	2.958219	2.209906
H	-2.347017	1.063073	3.407321
H	-0.770237	0.244574	3.506722
H	-2.170074	-0.407855	4.396147
H	-0.548569	-2.753179	2.198490
H	-1.958627	-3.377048	1.319779
H	-2.027585	-3.185963	3.091039
H	-4.589468	-1.825227	1.188637
H	-4.738573	-0.178811	1.849253
H	-4.567936	-1.569829	2.949941
H	-4.915701	0.798170	-1.945694
H	-5.329432	-0.435495	-0.727258
H	-5.467124	-0.812034	-2.463387
H	-1.371033	-0.917575	-3.609883
H	-2.295655	0.598698	-3.620235
H	-3.033342	-0.891335	-4.269723
H	-2.127177	-3.247943	-1.660713
H	-3.751901	-3.236205	-2.388747
H	-3.564328	-3.099394	-0.623363

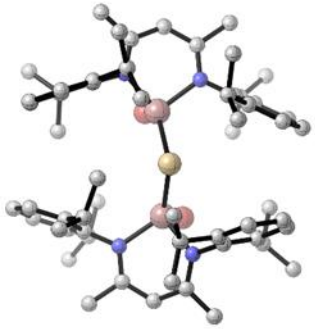

B3LYP-D3(BJ)/def2-svp
Thermal correction to Gibbs Energy = 1.167004
Thermal correction to Enthalpy = 1.356723
Sum of electronic and thermal Free Energies = −11762.772442
Sum of electronic and thermal Enthalpies = −11762.582723

Atomic Type	Coordinates (Angstroms) X	Y	Z
Br	-2.439699	0.021886	-2.515059
Ga	-1.943592	0.517499	-0.223192
Si	-0.061927	-0.441407	1.023850
N	-3.564436	-0.208849	0.723209
N	-2.512967	2.422661	-0.064928
C	-4.719760	0.448474	0.709157
C	-4.831241	1.803457	0.342025
H	-5.837163	2.217066	0.382834
C	-3.805455	2.734794	0.107969
C	-5.994885	-0.255616	1.108817
H	-5.818966	-1.031744	1.863724
H	-6.417343	-0.752750	0.220808
H	-6.740930	0.456886	1.483247
C	-4.210754	4.191268	0.112468
H	-5.284698	4.296981	0.305923
H	-3.969941	4.678093	-0.841378
H	-3.653045	4.738725	0.887619
C	-3.436245	-1.485572	1.362838
C	-3.705657	-2.682289	0.663500
C	-3.504073	-3.900624	1.329556
H	-3.700493	-4.834199	0.798537
C	-3.062728	-3.940686	2.647618
H	-2.912919	-4.900421	3.148093
C	-2.806349	-2.750842	3.329337
H	-2.458722	-2.791140	4.362129
C	-2.977378	-1.510127	2.706278
C	-4.220997	-2.694363	-0.767607
H	-4.320363	-1.652685	-1.099455
C	-3.225203	-3.373056	-1.715952
H	-3.131933	-4.447420	-1.493581

H	-2.227487	-2.924160	-1.638263
H	-3.555909	-3.265465	-2.760571
C	-5.600975	-3.364483	-0.867568
H	-6.330625	-2.910796	-0.180326
H	-5.544540	-4.438135	-0.625218
H	-5.995283	-3.277123	-1.892658
C	-2.712828	-0.216382	3.465758
H	-2.383307	0.520522	2.719348
C	-3.993224	0.338032	4.111741
H	-4.772136	0.549297	3.367683
H	-3.778840	1.278418	4.645380
H	-4.404571	-0.380230	4.839564
C	-1.569105	3.502165	-0.173923
C	-1.405758	4.197358	-1.394552
C	-0.596752	5.342043	-1.399721
H	-0.471682	5.898321	-2.330721
C	0.060290	5.774878	-0.251347
H	0.680762	6.673736	-0.280162
C	-0.041642	5.032598	0.923129
H	0.516200	5.342203	1.808498
C	-0.852615	3.893512	0.980558
C	-0.895502	3.046482	2.240229
H	-1.794227	2.417571	2.189834
C	-0.977864	3.852185	3.541648
H	-1.830453	4.548204	3.523101
H	-0.063980	4.439250	3.722481
H	-1.106920	3.175272	4.400883
C	0.330319	2.127008	2.261382
H	0.311006	1.429253	3.106239
H	1.267965	2.697327	2.302048
H	0.412723	1.579703	1.284749
C	-2.032438	3.719190	-2.695859
H	-2.730517	2.905392	-2.459064
C	-2.815841	4.816940	-3.429954
H	-3.313583	4.395153	-4.317147
H	-2.156509	5.627575	-3.779705
H	-3.591950	5.271708	-2.794360
C	-0.945083	3.126585	-3.607504
H	-1.397766	2.705759	-4.518736
H	-0.394833	2.316611	-3.110374
H	-0.219351	3.900649	-3.907746
Br	1.823291	0.260795	-2.840012
Ga	1.825085	-0.435075	-0.537533
N	3.590214	0.213071	0.123645
N	2.442187	-2.338541	-0.691466
C	4.714893	-0.389348	-0.285095
C	4.752169	-1.658899	-0.878790
H	5.735534	-2.007030	-1.190418
C	3.714494	-2.612974	-0.961888
C	6.032499	0.317717	-0.076219
H	6.179918	0.578057	0.981135

H	6.044643	1.265249	-0.634244
H	6.872831	-0.302464	-0.410387
C	4.119370	-4.006293	-1.381980
H	5.145095	-4.228526	-1.060319
H	4.090275	-4.070441	-2.481189
H	3.439248	-4.771669	-0.989688
C	3.665201	1.367866	0.968774
C	3.667288	2.666790	0.410057
C	3.699792	3.767010	1.278590
H	3.687400	4.775011	0.858865
C	3.759424	3.597657	2.660348
H	3.793982	4.468138	3.320048
C	3.777521	2.311182	3.196854
H	3.829682	2.181317	4.280131
C	3.720754	1.180491	2.371633
C	3.645701	2.898029	-1.092077
H	3.704020	1.919751	-1.584827
C	2.329768	3.549413	-1.529824
H	2.249835	4.574624	-1.142434
H	1.456895	2.988329	-1.168599
H	2.261319	3.586789	-2.627102
C	4.844536	3.734312	-1.565361
H	5.804391	3.292572	-1.254926
H	4.808898	4.760683	-1.165560
H	4.844480	3.807474	-2.664413
C	3.756449	-0.206935	2.999215
H	3.604462	-0.938478	2.194581
C	2.628341	-0.406031	4.019421
H	2.664723	0.345678	4.823403
H	2.709602	-1.396707	4.490730
H	1.643330	-0.348138	3.533905
C	5.125274	-0.500522	3.634079
H	5.941878	-0.396645	2.904427
H	5.154890	-1.529228	4.027701
H	5.334142	0.186403	4.470307
C	1.447750	-3.371744	-0.623349
C	0.845342	-3.874886	-1.800549
C	-0.124572	-4.878514	-1.670997
H	-0.598212	-5.275937	-2.570591
C	-0.510469	-5.358528	-0.424899
H	-1.279791	-6.130155	-0.347159
C	0.076554	-4.842000	0.727336
H	-0.241208	-5.217363	1.698956
C	1.067873	-3.854713	0.657365
C	1.762890	-3.376516	1.927747
H	1.987565	-2.309183	1.790778
C	3.103878	-4.096235	2.146551
H	3.806145	-3.920840	1.321034
H	2.954378	-5.184041	2.242443
H	3.584210	-3.736404	3.071073
C	0.879399	-3.497428	3.172141

H	1.372498	-3.026105	4.033628
H	0.693266	-4.548664	3.443665
H	-0.089952	-3.003036	3.019878
C	1.201648	-3.379604	-3.194887
H	1.997392	-2.629277	-3.098805
C	1.717481	-4.517105	-4.091625
H	2.066771	-4.113188	-5.055185
H	0.922474	-5.248015	-4.310801
H	2.550306	-5.067270	-3.628660
C	0.004653	-2.679555	-3.854204
H	0.298243	-2.260053	-4.829129
H	-0.370038	-1.850506	-3.241130
H	-0.826273	-3.384092	-4.022253
C	-1.598850	-0.338715	4.510269
H	-1.348895	0.656028	4.911926
H	-0.689737	-0.772794	4.070391
H	-1.902133	-0.962056	5.366429

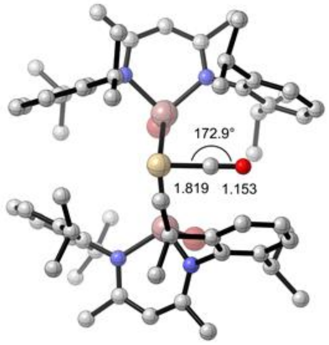

B3LYP-D3(BJ)/def2-svp
Thermal correction to Gibbs Energy = 1.171265
Thermal correction to Enthalpy = 1.368577
Sum of electronic and thermal Free Energies = −11876.060593
Sum of electronic and thermal Enthalpies = −11875.863282

Atomic Type	Coordinates (Angstroms) X	Y	Z
Br	1.908105	0.791871	-2.740777
Ga	2.022069	0.015700	-0.487611
Si	0.003260	-0.095780	0.880194
O	-0.140456	-3.045666	0.600496
C	-0.079676	-1.894403	0.621790
N	3.358621	1.252305	0.338620
N	3.271613	-1.520238	-0.565583
C	4.655601	1.090747	0.076676
C	5.192593	-0.049569	-0.554553
H	6.257976	-0.006576	-0.773671
C	4.576821	-1.291475	-0.763109
C	5.646984	2.163607	0.462319

H	5.320807	2.733650	1.340205
H	5.753080	2.874920	-0.371220
H	6.634218	1.725712	0.657149
C	5.455317	-2.436682	-1.205014
H	6.498378	-2.115612	-1.311922
H	5.100975	-2.831726	-2.168221
H	5.409317	-3.271579	-0.492271
C	2.903546	2.383319	1.096645
C	2.730274	3.640836	0.474521
C	2.285637	4.713388	1.260698
H	2.137443	5.688464	0.792818
C	2.034823	4.558794	2.619400
H	1.695163	5.408893	3.216010
C	2.209113	3.312075	3.218342
H	2.002157	3.200100	4.282453
C	2.631729	2.202862	2.476290
C	3.011445	3.876983	-1.002456
H	3.401466	2.944382	-1.429976
C	1.721346	4.211841	-1.760330
H	1.326521	5.191358	-1.452231
H	0.941618	3.462993	-1.578159
H	1.908348	4.240339	-2.844859
C	4.056042	4.983274	-1.223234
H	4.982721	4.801496	-0.659047
H	3.670590	5.967241	-0.911197
H	4.313951	5.055437	-2.291842
C	2.836801	0.850964	3.150675
H	2.561122	0.082108	2.413730
C	4.310060	0.617614	3.523533
H	4.964264	0.618602	2.641672
H	4.430366	-0.357188	4.022377
H	4.666305	1.398857	4.214491
C	2.808144	-2.877667	-0.481498
C	2.315719	-3.541798	-1.626281
C	1.844787	-4.853558	-1.480028
H	1.444103	-5.374707	-2.351981
C	1.884830	-5.504272	-0.250041
H	1.515018	-6.528318	-0.158785
C	2.392370	-4.842459	0.865489
H	2.416122	-5.355138	1.829446
C	2.849257	-3.522912	0.777277
C	3.364001	-2.822445	2.028725
H	3.533170	-1.767681	1.774282
C	4.712045	-3.401330	2.486770
H	5.474055	-3.326906	1.697009
H	4.615784	-4.464504	2.760667
H	5.088382	-2.858149	3.368558
C	2.340026	-2.862167	3.172255
H	2.740728	-2.349863	4.059881
H	2.100087	-3.894776	3.468983
H	1.399365	-2.367153	2.891999

C	2.283959	-2.883578	-2.996561
H	2.792253	-1.913858	-2.917008
C	3.029089	-3.713146	-4.053288
H	3.069381	-3.164133	-5.007371
H	2.526202	-4.674261	-4.246709
H	4.062950	-3.937730	-3.746185
C	0.843541	-2.600028	-3.439494
H	0.832130	-2.048619	-4.391939
H	0.307825	-1.981703	-2.708061
H	0.276598	-3.534942	-3.574426
Br	-2.260430	-0.499239	-2.704471
Ga	-2.093056	0.021676	-0.383934
N	-3.510069	-1.099793	0.462363
N	-3.107211	1.721626	-0.253002
C	-4.790630	-0.737382	0.350454
C	-5.211564	0.531600	-0.086208
H	-6.288678	0.665428	-0.165229
C	-4.443650	1.695663	-0.267287
C	-5.873709	-1.723206	0.718714
H	-5.631629	-2.263677	1.643115
H	-5.970461	-2.479472	-0.074504
H	-6.840584	-1.218935	0.835499
C	-5.207220	2.980852	-0.483915
H	-6.254337	2.871121	-0.176804
H	-5.186344	3.241718	-1.552984
H	-4.755822	3.822064	0.057021
C	-3.169427	-2.344171	1.097151
C	-3.190339	-3.556347	0.371828
C	-2.835742	-4.735985	1.040370
H	-2.839688	-5.679223	0.490495
C	-2.458150	-4.726246	2.378379
H	-2.177301	-5.656922	2.877102
C	-2.423743	-3.521316	3.076951
H	-2.115303	-3.519680	4.122606
C	-2.775026	-2.315564	2.458779
C	-3.519923	-3.630221	-1.111856
H	-3.819847	-2.628659	-1.448023
C	-2.271911	-4.019085	-1.919593
H	-1.934144	-5.036646	-1.664200
H	-1.440776	-3.332024	-1.726886
H	-2.486815	-3.985813	-2.998861
C	-4.673185	-4.601039	-1.409497
H	-5.574805	-4.368542	-0.822219
H	-4.394149	-5.642426	-1.182307
H	-4.941497	-4.557737	-2.476975
C	-2.750973	-1.013293	3.247986
H	-2.492853	-0.216145	2.537949
C	-1.684051	-0.993715	4.346487
H	-1.920128	-1.689689	5.166849
H	-1.616402	0.011891	4.786578
H	-0.693410	-1.251491	3.944949

C	-4.132925	-0.672057	3.828351
H	-4.884515	-0.526361	3.040716
H	-4.084444	0.259118	4.415088
H	-4.486408	-1.475805	4.494622
C	-2.419309	2.982178	-0.206483
C	-2.166886	3.719690	-1.385385
C	-1.542939	4.968241	-1.258559
H	-1.342126	5.553644	-2.157841
C	-1.157978	5.466414	-0.017708
H	-0.664312	6.438509	0.053684
C	-1.390335	4.716151	1.132485
H	-1.077508	5.106490	2.101381
C	-2.029583	3.472828	1.063058
C	-2.370213	2.712775	2.336720
H	-2.513616	1.661098	2.057778
C	-3.698966	3.202328	2.935383
H	-4.536876	3.063805	2.237806
H	-3.642523	4.273569	3.188907
H	-3.936403	2.646135	3.856251
C	-1.249522	2.758307	3.375889
H	-1.498912	2.114311	4.232394
H	-1.086822	3.773915	3.769269
H	-0.301469	2.409412	2.947292
C	-2.540560	3.214384	-2.771409
H	-3.110179	2.283155	-2.650794
C	-3.417951	4.215360	-3.539762
H	-3.760652	3.770283	-4.487248
H	-2.860976	5.132130	-3.791338
H	-4.305921	4.519090	-2.964202
C	-1.285416	2.868476	-3.585563
H	-1.568068	2.435048	-4.557613
H	-0.656058	2.132417	-3.069721
H	-0.675959	3.767110	-3.774402
C	1.935349	0.642738	4.371026
H	2.030692	-0.388721	4.738035
H	0.879433	0.813277	4.121700
H	2.208462	1.308945	5.204690

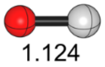

M06-2X-D3/def2-svp
Thermal correction to Gibbs Energy = −0.013825
Thermal correction to Enthalpy = 0.008595
Sum of electronic and thermal Free Energies = −113.199466
Sum of electronic and thermal Enthalpies = −113.177046

Atomic Type	Coordinates (Angstroms) X	Y	Z
C	0.000000	0.000000	1.914012
O	0.000000	0.000000	3.037977

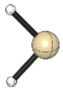

M06-2X-D3/def2-svp
Thermal correction to Gibbs Energy = −0.007815
Thermal correction to Enthalpy = 0.015728
Sum of electronic and thermal Free Energies = −290.499524
Sum of electronic and thermal Enthalpies = −290.475981

Atomic Type	Coordinates (Angstroms) X	Y	Z
Si	0.000000	-0.000000	0.119226
H	0.000000	1.090817	-0.944387
H	-0.000000	-1.090817	-0.944387

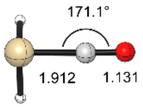

M06-2X-D3/def2-svp
Thermal correction to Gibbs Energy = −0.003313
Thermal correction to Enthalpy = 0.027682
Sum of electronic and thermal Free Energies = −403.723746
Sum of electronic and thermal Enthalpies = −403.692751

Atomic Type	Coordinates (Angstroms) X	Y	Z
C	0.000000	0.714660	-0.000000
O	-0.143250	1.836150	-0.000000
Si	-0.052993	-1.196220	0.000000
H	0.943953	-1.115036	1.137295
H	0.943953	-1.115036	-1.137295

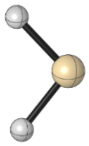

M06-2X-D3/def2-svp
Thermal correction to Gibbs Energy = 0.043401
Thermal correction to Enthalpy = 0.077629
Sum of electronic and thermal Free Energies = −369.003684
Sum of electronic and thermal Enthalpies = −368.969456

Atomic Type	Coordinates (Angstroms) X	Y	Z
Si	0.000008	-0.762935	-0.000000
C	-1.428548	0.502411	-0.011874
H	-2.402365	0.072598	-0.282478
H	-1.508548	0.862413	1.032768
H	-1.229437	1.391009	-0.632447
C	1.428558	0.502409	0.011871
H	1.508262	0.862640	-1.032714
H	1.229466	1.390869	0.632655
H	2.402450	0.072643	0.282239

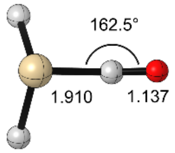

M06-2X-D3/def2-svp
Thermal correction to Gibbs Energy = 0.049845
Thermal correction to Enthalpy = 0.089823
Sum of electronic and thermal Free Energies = −482.209100
Sum of electronic and thermal Enthalpies = −482.169122

Atomic Type	Coordinates (Angstroms) X	Y	Z
C	-1.322354	-0.000021	0.080713
O	-2.456625	-0.000020	0.159319
Si	0.454653	-0.000000	-0.618428
C	1.124990	-1.529581	0.328286
H	0.532285	-2.430001	0.111907
H	2.141235	-1.721812	-0.048581
H	1.187807	-1.391011	1.416566
C	1.124931	1.529611	0.328281
H	1.187758	1.391047	1.416562
H	2.141167	1.721880	-0.048590
H	0.532190	2.430007	0.111902

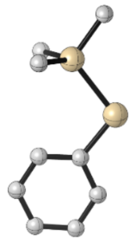

M06-2X-D3/def2-svp
Thermal correction to Gibbs Energy = 0.161023
Thermal correction to Enthalpy = 0.218495
Sum of electronic and thermal Free Energies = −929.543040
Sum of electronic and thermal Enthalpies = −929.485568

Atomic Type	Coordinates (Angstroms) X	Y	Z
Si	-0.352237	-1.589808	-0.000985
Si	-2.067901	0.107513	0.000057
C	1.237152	-0.546265	-0.000500
C	2.445587	-1.274983	0.000045
C	1.335798	0.861304	-0.000610
C	3.686198	-0.639356	0.000488
H	2.404583	-2.367984	0.000099
C	2.570021	1.504952	-0.000254
H	0.429718	1.469766	-0.001016
C	3.747215	0.753375	0.000320
H	4.604676	-1.228413	0.000938
H	2.618065	2.595119	-0.000400
H	4.715354	1.257588	0.000643
C	-3.728060	-0.801084	0.000389
H	-3.825462	-1.444442	0.887952
H	-4.567812	-0.088753	0.001589
H	-3.826722	-1.442935	-0.888124
C	-2.021342	1.194407	1.555293
H	-2.119212	0.577108	2.461107
H	-1.093995	1.777086	1.646610
H	-2.868490	1.898517	1.539126
C	-2.022835	1.196082	-1.554039
H	-2.870399	1.899661	-1.536580
H	-1.095921	1.779466	-1.645251
H	-2.120846	0.579752	-2.460494

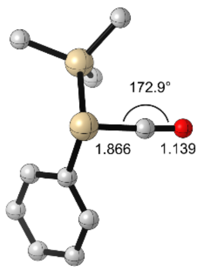

M06-2X-D3/def2-svp
Thermal correction to Gibbs Energy = 0.166500
Thermal correction to Enthalpy = 0.229281
Sum of electronic and thermal Free Energies = −1042.766161
Sum of electronic and thermal Enthalpies = −1042.703381

Atomic Type	Coordinates (Angstroms) X	Y	Z
Si	0.366356	0.660369	-1.224807
Si	1.965260	-0.567444	0.080967
C	-1.337083	0.144912	-0.493007
C	-2.384965	1.064486	-0.327809
C	-1.600379	-1.203959	-0.207214
C	-3.635600	0.659283	0.136097
H	-2.227782	2.119910	-0.569367
C	-2.854322	-1.616929	0.243408
H	-0.814518	-1.953757	-0.337463
C	-3.874762	-0.684446	0.423136
H	-4.429993	1.396303	0.266566
H	-3.032945	-2.672210	0.457282
H	-4.854975	-1.004382	0.779616
C	3.577099	0.417227	0.076698
H	3.468134	1.379100	0.600139
H	4.367117	-0.153888	0.588940
H	3.915024	0.621036	-0.949933
C	1.417136	-0.871003	1.860400
H	1.345355	0.080429	2.408658
H	0.432000	-1.357897	1.901424
H	2.142583	-1.514022	2.383456
C	2.260555	-2.217889	-0.789767
H	3.041449	-2.784077	-0.258028
H	1.355378	-2.841870	-0.819393
H	2.596259	-2.056125	-1.824479
C	0.629437	2.010304	0.036166
O	0.858946	2.903572	0.704712

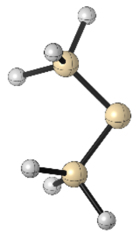

M06-2X-D3/def2-svp
Thermal correction to Gibbs Energy = 0.178779
Thermal correction to Enthalpy = 0.241834
Sum of electronic and thermal Free Energies = −1107.138335
Sum of electronic and thermal Enthalpies = −1107.075280

Atomic Type	Coordinates (Angstroms) X	Y	Z
Si	0.000012	-1.600726	-0.000683
Si	-1.785303	-0.011821	0.007374
C	-3.396700	-0.861700	0.515726
H	-3.353086	-1.200301	1.561856
H	-4.252007	-0.174798	0.416422
H	-3.587343	-1.743994	-0.113103
C	-1.592262	1.521285	1.104366
H	-1.520494	1.238068	2.165543
H	-0.704839	2.117665	0.850433
H	-2.480018	2.163937	0.991678
C	-1.956626	0.519288	-1.808824
H	-2.832072	1.180398	-1.911122
H	-1.072917	1.065312	-2.167757
H	-2.118607	-0.348098	-2.466526
Si	1.785292	-0.011802	-0.007442
C	1.956755	0.517246	1.809352
H	2.832187	1.178271	1.912322
H	1.073061	1.062837	2.168983
H	2.118827	-0.350877	2.466057
C	3.396638	-0.861131	-0.516870
H	3.587311	-1.744129	0.110961
H	3.352946	-1.198558	-1.563376
H	4.251961	-0.174352	-0.416857
C	1.592192	1.522552	-1.102670
H	2.479939	2.165091	-0.989276
H	1.520403	1.240533	-2.164165
H	0.704762	2.118631	-0.848048

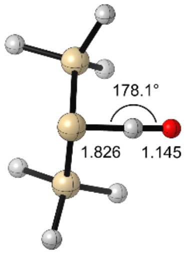

M06-2X-D3/def2-svp
Thermal correction to Gibbs Energy = 0.186864
Thermal correction to Enthalpy = 0.253188
Sum of electronic and thermal Free Energies = −1220.378218
Sum of electronic and thermal Enthalpies = −1220.311894

Atomic Type	Coordinates (Angstroms) X	Y	Z
Si	-0.009041	0.334418	-1.391541

Si	1.818699	-0.356492	0.015699
C	3.327834	0.668948	-0.466297
H	3.172646	1.733629	-0.234762
H	4.216950	0.328567	0.087501
H	3.536097	0.581155	-1.542466
C	1.554124	-0.171771	1.877880
H	1.353474	0.877740	2.143143
H	0.716077	-0.784521	2.241161
H	2.461869	-0.488264	2.415955
C	2.128354	-2.170712	-0.405417
H	2.986376	-2.551479	0.170572
H	1.254811	-2.796442	-0.172702
H	2.354768	-2.283733	-1.475778
Si	-1.861647	-0.288058	0.014215
C	-2.098061	0.740895	1.580252
H	-2.958468	0.356278	2.150502
H	-1.212451	0.707877	2.230840
H	-2.300153	1.794607	1.335983
C	-3.420643	-0.133486	-1.037979
H	-3.577340	0.908715	-1.353417
H	-3.344148	-0.751419	-1.944583
H	-4.306851	-0.457660	-0.469845
C	-1.618463	-2.103969	0.479569
H	-2.516526	-2.479312	0.995208
H	-1.458183	-2.722243	-0.416576
H	-0.758994	-2.245040	1.151357
C	0.085510	1.850845	-0.378638
O	0.169496	2.816114	0.231808

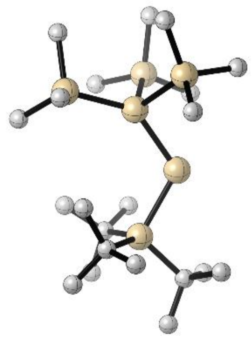

M06-2X-D3/def2-svp
Thermal correction to Gibbs Energy = 0.634170
Thermal correction to Enthalpy = 0.754615
Sum of electronic and thermal Free Energies = −2567.468308
Sum of electronic and thermal Enthalpies = −2567.347863

Atomic Type	Coordinates (Angstroms) X	Y	Z
Si	-0.455404	-0.882343	1.094506

Si	-2.468650	-0.031819	0.083881
Si	1.510800	-0.039150	0.092756
Si	2.205340	1.662261	-1.414074
Si	2.554541	-2.048416	-0.614749
Si	2.546352	0.450904	2.170405
C	-2.632265	1.904820	-0.035601
C	-3.686432	2.321828	-1.073462
C	-1.277176	2.513329	-0.417508
C	-3.016363	2.527363	1.315387
C	-2.393647	-0.898362	-1.670014
C	-3.769583	-1.072240	-2.326904
C	-1.492077	-0.108569	-2.627518
C	-1.730085	-2.279631	-1.512573
C	-3.888279	-0.791615	1.192684
C	-5.283421	-0.273205	0.810902
C	-3.895285	-2.324841	1.082940
C	-3.631141	-0.472765	2.676660
C	2.103874	3.386530	-0.639977
C	4.039929	1.316147	-1.749135
C	1.392107	1.716580	-3.122748
C	2.283915	-2.224012	-2.481486
C	1.815930	-3.557966	0.251297
C	4.414858	-2.046497	-0.269085
C	4.269743	1.207292	1.976031
C	1.437977	1.686777	3.086404
C	2.700701	-1.107127	3.228659
H	-3.790944	3.421171	-1.074354
H	-4.676874	1.896179	-0.854813
H	-3.409051	2.021248	-2.093670
H	-1.384815	3.603834	-0.560033
H	-0.861055	2.091548	-1.341889
H	-0.533579	2.350552	0.378671
H	-4.031407	2.256156	1.635389
H	-2.983547	3.627190	1.227010
H	-2.314935	2.242530	2.115840
H	-4.285904	-0.109966	-2.461892
H	-4.430436	-1.730995	-1.746498
H	-3.649453	-1.526603	-3.326445
H	-1.896292	0.882012	-2.879597
H	-1.382280	-0.666965	-3.574402
H	-0.484368	0.028868	-2.206880
H	-2.265556	-2.953892	-0.832633
H	-0.686284	-2.183426	-1.154813
H	-1.665124	-2.778755	-2.496032
H	-5.388622	0.806537	0.989507
H	-6.049132	-0.779601	1.424649
H	-5.527630	-0.464754	-0.244535
H	-4.180723	-2.678301	0.082489
H	-4.630706	-2.736227	1.795908
H	-2.914733	-2.757883	1.340033
H	-4.411809	-0.952665	3.292522

H	-3.655535	0.600918	2.898600
H	-2.661283	-0.872775	3.014339
H	1.068683	3.739118	-0.539697
H	2.645077	4.100342	-1.281285
H	2.570548	3.409144	0.356235
H	4.458822	2.138810	-2.350920
H	4.174382	0.383672	-2.318046
H	4.625446	1.239103	-0.822018
H	1.981603	2.401696	-3.753031
H	0.357743	2.085023	-3.103582
H	1.394389	0.727586	-3.604434
H	2.777167	-1.410718	-3.035359
H	1.212174	-2.200845	-2.733054
H	2.698128	-3.178511	-2.843117
H	0.755988	-3.704039	-0.006192
H	1.879080	-3.458118	1.344885
H	2.367025	-4.463837	-0.048406
H	4.616011	-2.014349	0.812235
H	4.919842	-1.189182	-0.735522
H	4.866448	-2.969081	-0.667728
H	4.250540	2.143719	1.399365
H	4.952367	0.507464	1.470742
H	4.687029	1.430807	2.970899
H	0.445477	1.245751	3.281038
H	1.290052	2.605589	2.498308
H	1.872228	1.969095	4.058498
H	1.720782	-1.588376	3.364358
H	3.102641	-0.848432	4.221232
H	3.381022	-1.840508	2.770273

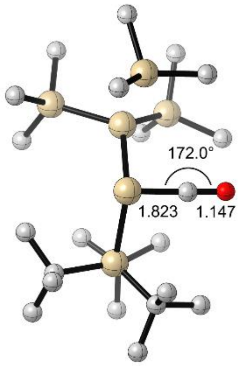

M06-2X-D3/def2-svp
Thermal correction to Gibbs Energy = 0.640158
Thermal correction to Enthalpy = 0.766409
Sum of electronic and thermal Free Energies = −2680.701912
Sum of electronic and thermal Enthalpies = −2680.575662

Atomic Type	Coordinates (Angstroms) X	Y	Z

Si	0.440854	-0.389037	-1.109174
Si	2.561485	0.064270	-0.082836
Si	-1.681982	0.019001	-0.077350
Si	-2.291373	2.296131	0.103817
Si	-2.321551	-1.062013	1.924170
Si	-3.067660	-0.844765	-1.798574
O	0.311205	-3.300785	-0.572054
C	0.373402	-2.160485	-0.683723
C	2.997429	1.760841	-0.955335
C	4.160247	2.492933	-0.265309
C	1.778865	2.697818	-0.948427
C	3.358142	1.539164	-2.432747
C	2.439864	0.242075	1.851319
C	3.819024	0.336143	2.523659
C	1.629069	1.494146	2.211443
C	1.676458	-0.948738	2.447478
C	3.813537	-1.343482	-0.601800
C	5.263567	-0.831076	-0.535556
C	3.720943	-2.569172	0.322530
C	3.537744	-1.844912	-2.030723
C	-2.309749	3.097049	-1.607922
C	-4.048812	2.328808	0.811823
C	-1.276786	3.369188	1.284581
C	-1.864286	-0.031753	3.443837
C	-1.605005	-2.804879	2.071288
C	-4.206571	-1.252667	1.907252
C	-4.853951	-0.224421	-1.694500
C	-2.391455	-0.359992	-3.495026
C	-3.095995	-2.735102	-1.690756
H	4.411315	3.403713	-0.836218
H	5.071185	1.880705	-0.204052
H	3.897232	2.812273	0.753607
H	2.043396	3.647251	-1.445341
H	1.438179	2.938120	0.064694
H	0.927242	2.272539	-1.505565
H	4.294649	0.981492	-2.565539
H	3.489189	2.519512	-2.922390
H	2.558703	1.005304	-2.970951
H	4.429232	1.159238	2.124973
H	4.395599	-0.593769	2.419121
H	3.687105	0.515909	3.604839
H	2.149486	2.427257	1.952419
H	1.447629	1.514464	3.300709
H	0.645897	1.494417	1.712941
H	2.156198	-1.917147	2.256405
H	0.655306	-0.991778	2.043483
H	1.600175	-0.825947	3.542575
H	5.466511	-0.046087	-1.276353
H	5.953243	-1.666121	-0.748970
H	5.523836	-0.435663	0.457503
H	4.044719	-2.347260	1.348564

H	4.388182	-3.357925	-0.065445
H	2.708100	-2.994534	0.367586
H	4.325518	-2.562436	-2.319025
H	3.527431	-1.041959	-2.778119
H	2.576422	-2.375161	-2.101455
H	-1.325809	3.039354	-2.097011
H	-2.590290	4.159134	-1.528932
H	-3.042244	2.600570	-2.262440
H	-4.434244	3.360498	0.775314
H	-4.047743	2.012017	1.866227
H	-4.745142	1.683019	0.260000
H	-1.799706	4.332302	1.399270
H	-0.264418	3.579467	0.917512
H	-1.194990	2.908355	2.280134
H	-2.391125	0.934883	3.425953
H	-0.785263	0.169964	3.499618
H	-2.162189	-0.560212	4.363327
H	-0.507798	-2.819852	2.124923
H	-1.907128	-3.424535	1.213649
H	-1.996285	-3.278084	2.986107
H	-4.531585	-1.919887	1.094513
H	-4.719792	-0.288115	1.783948
H	-4.539477	-1.696760	2.859082
H	-4.913103	0.858247	-1.882770
H	-5.311514	-0.429903	-0.715915
H	-5.456067	-0.731276	-2.465450
H	-1.410589	-0.823716	-3.673877
H	-2.266176	0.729478	-3.584652
H	-3.084926	-0.692851	-4.283631
H	-2.087625	-3.164397	-1.786521
H	-3.713570	-3.145584	-2.505489
H	-3.523452	-3.081399	-0.737532

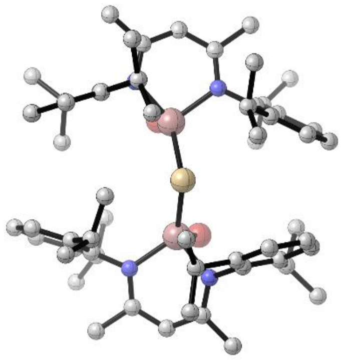

M06-2X-D3/def2-svp
Thermal correction to Gibbs Energy = 1.176387
Thermal correction to Enthalpy = 1.362852
Sum of electronic and thermal Free Energies = − 11761.158145
Sum of electronic and thermal Enthalpies = −11760.971679

Atomic Type	Coordinates (Angstroms) X	Y	Z
Br	-2.493143	-0.212306	-2.538123
Ga	-2.009552	0.295949	-0.236853
Si	-0.020131	-0.463907	0.995536
N	-3.507006	-0.604162	0.746730
N	-2.790693	2.118084	-0.041196
C	-4.735150	-0.108821	0.705513
C	-5.020135	1.209682	0.302690
H	-6.070348	1.489434	0.312253
C	-4.111684	2.262474	0.090805
C	-5.892499	-0.971862	1.140887
H	-5.709644	-1.400298	2.136157
H	-5.994526	-1.817889	0.445718
H	-6.828037	-0.402890	1.153033
C	-4.701517	3.652332	0.059594
H	-5.778072	3.619994	0.253679
H	-4.523657	4.128706	-0.913423
H	-4.214996	4.287304	0.814153
C	-3.247141	-1.847864	1.410032
C	-3.379825	-3.071942	0.727519
C	-3.076826	-4.254071	1.415396
H	-3.167161	-5.209206	0.893355
C	-2.659677	-4.231038	2.738843
H	-2.430763	-5.162694	3.259154
C	-2.518801	-3.011597	3.398015
H	-2.168338	-3.001093	4.430563
C	-2.795544	-1.805722	2.751702
C	-3.787972	-3.149321	-0.732899
H	-4.069754	-2.140866	-1.066948
C	-2.597313	-3.597824	-1.585968
H	-2.343941	-4.647755	-1.371291
H	-1.701072	-2.990265	-1.386299
H	-2.830870	-3.501140	-2.656857
C	-4.981228	-4.083573	-0.959249
H	-5.847629	-3.809518	-0.339690
H	-4.722283	-5.126757	-0.722640
H	-5.290095	-4.051215	-2.014156
C	-2.606532	-0.479067	3.472024
H	-2.269236	0.237795	2.708490
C	-3.917288	0.060439	4.056498
H	-4.660937	0.269808	3.275683
H	-3.733439	1.000985	4.598644
H	-4.351277	-0.662036	4.765239
C	-1.990797	3.309203	-0.136347
C	-1.929493	4.037813	-1.343993
C	-1.271101	5.271858	-1.339901
H	-1.230097	5.853129	-2.262970
C	-0.661137	5.766300	-0.193004
H	-0.161181	6.736187	-0.212569

C	-0.653741	4.999234	0.966667
H	-0.134238	5.364727	1.854014
C	-1.312978	3.767870	1.013599
C	-1.235462	2.910759	2.263026
H	-2.084774	2.212871	2.242501
C	-1.320697	3.700825	3.570022
H	-2.208144	4.349306	3.584638
H	-0.431360	4.329764	3.723726
H	-1.383177	3.010948	4.424376
C	0.067734	2.104135	2.231820
H	0.134405	1.393382	3.066842
H	0.945177	2.768701	2.260429
H	0.172816	1.591817	1.235978
C	-2.493748	3.499708	-2.648138
H	-3.138434	2.638884	-2.419116
C	-3.322456	4.529123	-3.422343
H	-3.782017	4.051679	-4.299507
H	-2.697250	5.355646	-3.792144
H	-4.127637	4.966101	-2.813165
C	-1.339208	2.985747	-3.518508
H	-1.729297	2.501979	-4.426179
H	-0.724269	2.244076	-2.988395
H	-0.687870	3.822686	-3.819313
Br	1.761101	0.443673	-2.881941
Ga	1.862844	-0.242530	-0.570166
N	3.531338	0.611087	0.084702
N	2.714908	-2.047565	-0.701620
C	4.713804	0.167476	-0.344053
C	4.911052	-1.082235	-0.949261
H	5.923874	-1.298373	-1.279911
C	4.001496	-2.161445	-0.996886
C	5.925796	1.040190	-0.137422
H	6.095828	1.216975	0.933735
H	5.756533	2.025385	-0.594999
H	6.819702	0.582768	-0.573564
C	4.555621	-3.507637	-1.390674
H	5.650633	-3.495571	-1.390397
H	4.203767	-3.764476	-2.400262
H	4.195156	-4.295728	-0.715752
C	3.474756	1.753341	0.946168
C	3.292155	3.043794	0.406134
C	3.195517	4.128229	1.285201
H	3.040593	5.129469	0.877104
C	3.306671	3.954029	2.661308
H	3.240484	4.813089	3.331089
C	3.510380	2.678378	3.178325
H	3.607617	2.543977	4.258097
C	3.591124	1.560829	2.340788
C	3.199525	3.278611	-1.090339
H	3.431339	2.333403	-1.599178
C	1.777050	3.682267	-1.484138

H	1.542905	4.682655	-1.093145
H	1.022854	2.982362	-1.086524
H	1.666856	3.695103	-2.578518
C	4.201875	4.332190	-1.572841
H	5.234354	4.080851	-1.288226
H	3.973459	5.325555	-1.157546
H	4.159770	4.414193	-2.668531
C	3.836840	0.191134	2.956758
H	3.842332	-0.552865	2.145737
C	2.723488	-0.193154	3.935220
H	2.615366	0.557627	4.733397
H	2.957235	-1.155469	4.414483
H	1.755255	-0.296327	3.419983
C	5.201547	0.133042	3.653660
H	6.020798	0.394574	2.969408
H	5.391758	-0.879871	4.038419
H	5.239340	0.829552	4.505340
C	1.876526	-3.209209	-0.622137
C	1.363340	-3.805812	-1.794345
C	0.560690	-4.943618	-1.660792
H	0.157471	-5.414891	-2.559615
C	0.241617	-5.461527	-0.413271
H	-0.398655	-6.341731	-0.331195
C	0.724807	-4.843493	0.734869
H	0.451922	-5.247047	1.709247
C	1.557765	-3.722130	0.659443
C	2.146353	-3.123114	1.931277
H	2.187926	-2.032291	1.792659
C	3.584273	-3.598934	2.171601
H	4.263559	-3.275138	1.370214
H	3.626774	-4.697195	2.241324
H	3.966338	-3.182336	3.117155
C	1.276449	-3.392526	3.158536
H	1.643841	-2.814568	4.017239
H	1.301870	-4.454211	3.448887
H	0.230322	-3.105327	2.972155
C	1.599498	-3.243376	-3.186858
H	2.330624	-2.425231	-3.114514
C	2.140854	-4.297493	-4.159785
H	2.399090	-3.825226	-5.118595
H	1.386021	-5.070479	-4.368680
H	3.035962	-4.807588	-3.774868
C	0.299445	-2.644520	-3.735408
H	0.480825	-2.155390	-4.704012
H	-0.119595	-1.885593	-3.060742
H	-0.457748	-3.432569	-3.878718
C	-1.522934	-0.533757	4.548815
H	-1.304115	0.482306	4.911737
H	-0.593811	-0.970498	4.151301
H	-1.842584	-1.123381	5.421638

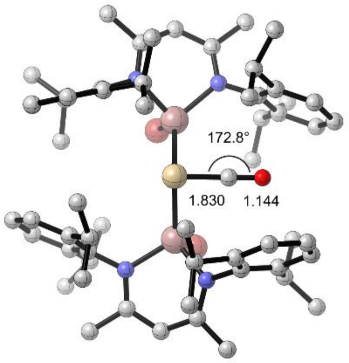

M06-2X-D3/def2-svp
Thermal correction to Gibbs Energy = 1.182329
Thermal correction to Enthalpy = 1.375008
Sum of electronic and thermal Free Energies = −11874.403787
Sum of electronic and thermal Enthalpies = −11874.211107

Atomic Type	Coordinates (Angstroms) X	Y	Z
Br	1.710815	0.941095	-2.782940
Ga	1.994766	0.163063	-0.533073
Si	0.026870	-0.083293	0.900616
O	0.163530	-3.044170	0.742803
C	0.104697	-1.902248	0.715758
N	3.268920	1.476858	0.255200
N	3.362219	-1.259576	-0.676836
C	4.549157	1.442364	-0.102817
C	5.144875	0.366040	-0.788877
H	6.183413	0.501482	-1.079278
C	4.623856	-0.927278	-0.947233
C	5.447936	2.597729	0.261665
H	5.383874	2.813219	1.337548
H	5.121110	3.506630	-0.261578
H	6.487302	2.382337	-0.006188
C	5.569932	-2.006191	-1.408143
H	6.547700	-1.586305	-1.665390
H	5.148679	-2.524774	-2.280987
H	5.696776	-2.765299	-0.623679
C	2.812069	2.559143	1.079753
C	2.500394	3.808103	0.504800
C	2.122235	4.853869	1.354764
H	1.870529	5.824543	0.922555
C	2.070152	4.679558	2.731515
H	1.789957	5.511010	3.380424
C	2.352744	3.432101	3.280720
H	2.275348	3.296577	4.359604
C	2.703991	2.347193	2.472616
C	2.523127	4.041540	-0.995981
H	2.991560	3.171545	-1.476499
C	1.089092	4.144839	-1.524096

H	0.623737	5.077021	-1.172063
H	0.461269	3.307570	-1.180127
H	1.080168	4.137600	-2.624244
C	3.312742	5.292825	-1.395114
H	4.340255	5.286724	-1.003092
H	2.824134	6.208488	-1.029571
H	3.366831	5.365562	-2.490927
C	2.975446	0.982113	3.089506
H	2.646839	0.227372	2.357082
C	4.469920	0.752872	3.343015
H	5.049275	0.753729	2.408111
H	4.629467	-0.221560	3.830662
H	4.875766	1.534810	4.003985
C	3.016954	-2.648967	-0.576518
C	2.509327	-3.337776	-1.696694
C	2.119931	-4.670891	-1.533340
H	1.702829	-5.210205	-2.386482
C	2.264969	-5.320954	-0.312999
H	1.957619	-6.362390	-0.206702
C	2.807750	-4.639237	0.769913
H	2.925492	-5.154256	1.725943
C	3.183198	-3.297536	0.665194
C	3.739681	-2.589531	1.892197
H	3.956813	-1.545049	1.620745
C	5.051378	-3.230468	2.360237
H	5.803466	-3.249627	1.558892
H	4.887410	-4.267185	2.691724
H	5.472538	-2.669216	3.207751
C	2.719363	-2.573615	3.035904
H	3.166138	-2.123942	3.935279
H	2.397672	-3.593312	3.296241
H	1.820357	-1.993141	2.777096
C	2.379669	-2.680228	-3.059644
H	2.874111	-1.699623	-3.013683
C	3.063462	-3.499199	-4.159221
H	3.029325	-2.950374	-5.111416
H	2.557605	-4.463870	-4.315637
H	4.117104	-3.709693	-3.921939
C	0.909853	-2.434379	-3.403876
H	0.817602	-1.858209	-4.336295
H	0.400030	-1.854932	-2.620516
H	0.373642	-3.389082	-3.525286
Br	-2.285926	-0.654456	-2.679490
Ga	-2.091518	-0.135724	-0.350747
N	-3.411476	-1.356315	0.497358
N	-3.230935	1.479510	-0.162774
C	-4.712609	-1.117505	0.366987
C	-5.237442	0.121332	-0.038385
H	-6.319983	0.172605	-0.120738
C	-4.556700	1.348048	-0.160272
C	-5.696987	-2.218400	0.673953

H	-5.452123	-2.726691	1.615742
H	-5.642572	-2.976645	-0.121924
H	-6.719409	-1.829026	0.720850
C	-5.417442	2.583725	-0.263745
H	-6.472099	2.334783	-0.108766
H	-5.299239	3.043853	-1.254132
H	-5.106326	3.336684	0.473826
C	-2.957350	-2.534555	1.177075
C	-2.821117	-3.759442	0.496845
C	-2.325948	-4.858829	1.206596
H	-2.204456	-5.812973	0.689836
C	-1.959959	-4.751759	2.541641
H	-1.560654	-5.617834	3.071462
C	-2.098771	-3.533897	3.201111
H	-1.813068	-3.457002	4.250899
C	-2.605695	-2.411815	2.541562
C	-3.125344	-3.915024	-0.983053
H	-3.589020	-2.984190	-1.341845
C	-1.823093	-4.101998	-1.767972
H	-1.318646	-5.036176	-1.471957
H	-1.126657	-3.272182	-1.590417
H	-2.027620	-4.136914	-2.848774
C	-4.087074	-5.075222	-1.259432
H	-5.011666	-4.996493	-0.668904
H	-3.622855	-6.043625	-1.019138
H	-4.358538	-5.095667	-2.324869
C	-2.840919	-1.115007	3.300586
H	-2.798040	-0.293509	2.571432
C	-1.787282	-0.839336	4.372203
H	-1.852494	-1.555579	5.205199
H	-1.945593	0.162909	4.798220
H	-0.771044	-0.878291	3.951442
C	-4.248916	-1.102023	3.911033
H	-5.027100	-1.138983	3.135705
H	-4.407368	-0.185970	4.499267
H	-4.386875	-1.966954	4.578485
C	-2.674039	2.804658	-0.161903
C	-2.640836	3.570533	-1.347470
C	-2.207651	4.897034	-1.262187
H	-2.186482	5.507392	-2.167144
C	-1.782125	5.444016	-0.057669
H	-1.444696	6.481267	-0.016018
C	-1.753628	4.656020	1.087214
H	-1.384000	5.079347	2.021508
C	-2.205325	3.332533	1.060465
C	-2.242343	2.507124	2.336115
H	-2.075809	1.461136	2.047131
C	-3.611707	2.575211	3.020050
H	-4.404736	2.138987	2.394590
H	-3.881440	3.618455	3.247719
H	-3.589364	2.014558	3.967567

C	-1.120897	2.870684	3.304202
H	-1.084500	2.141533	4.126947
H	-1.266052	3.864838	3.755111
H	-0.146792	2.862993	2.794367
C	-3.002152	2.996734	-2.707993
H	-3.494627	2.024929	-2.557220
C	-3.947452	3.897736	-3.509628
H	-4.259866	3.384226	-4.430208
H	-3.451319	4.832465	-3.810824
H	-4.852365	4.169673	-2.946451
C	-1.720130	2.738694	-3.509038
H	-1.955258	2.237992	-4.460049
H	-1.018002	2.094443	-2.961742
H	-1.211471	3.691075	-3.730572
C	2.163151	0.753789	4.364371
H	2.244718	-0.293580	4.684442
H	1.099346	0.977901	4.195761
H	2.524377	1.377178	5.196311

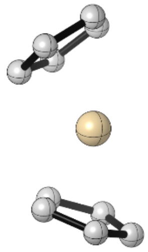

M06-2X-D3/def2-svp
Thermal correction to Gibbs Energy = 0.129779
Thermal correction to Enthalpy = 0.179251
Sum of electronic and thermal Free Energies = −675.815345
Sum of electronic and thermal Enthalpies = −675.765873

Atomic Type	Coordinates (Angstroms) X	Y	Z
C	2.432995	0.984270	-0.518611
C	1.752522	0.963897	0.725248
C	1.576748	-0.401286	1.099505
C	2.119406	-1.199673	0.057132
C	2.661489	-0.339637	-0.920948
C	-2.125568	1.199219	0.046259
C	-2.663583	0.327760	-0.923677
C	-2.428284	-0.991377	-0.509384
C	-1.747779	-0.956298	0.734180
C	-1.578181	0.413247	1.095464
H	-2.674648	-1.893138	-1.066153
H	-3.109974	0.629929	-1.868493
H	-2.128832	2.286819	0.008210
H	-1.150654	0.786193	2.024235

H	-1.453778	-1.816535	1.332897
H	3.106021	-0.652716	-1.863087
H	1.462253	1.831001	1.315832
H	2.683244	1.879643	-1.083880
H	1.148144	-0.763474	2.032022
H	2.117872	-2.287595	0.029359
Si	0.000126	-0.000061	-0.440854

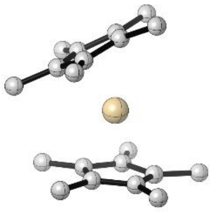

M06-2X-D3/def2-svp
Thermal correction to Gibbs Energy = 0.385654
Thermal correction to Enthalpy = 0.472576
Sum of electronic and thermal Free Energies = −1068.235645
Sum of electronic and thermal Enthalpies = −1068.148723

Atomic Type	Coordinates (Angstroms) X	Y	Z
C	1.892341	1.028082	0.670212
C	1.777816	-0.306821	1.160727
C	2.073626	-1.186436	0.077365
C	2.404688	-0.401159	-1.056793
C	2.279749	0.959294	-0.699973
C	-2.080406	1.186830	0.060238
C	-2.407738	0.383070	-1.061742
C	-2.274331	-0.971219	-0.684937
C	-1.886594	-1.017466	0.686297
C	-1.779064	0.325294	1.156474
Si	-0.000088	0.000784	-0.335258
C	-1.518569	0.779727	2.562642
H	-2.419594	1.246342	2.990537
H	-1.237894	-0.058532	3.212555
H	-0.709338	1.523863	2.611645
C	-1.704347	-2.284244	1.471629
H	-2.594218	-2.926058	1.384094
H	-0.843327	-2.873071	1.115063
H	-1.550886	-2.080465	2.539797
C	-2.468746	-2.184604	-1.543728
H	-1.684056	-2.933304	-1.357051
H	-3.437363	-2.669446	-1.340618
H	-2.441078	-1.934454	-2.612497
C	-2.735519	0.927116	-2.419114
H	-2.863623	0.125119	-3.157462
H	-3.668403	1.512013	-2.399355

H	-1.939645	1.594948	-2.785708
C	-2.105170	2.686223	0.088327
H	-1.545744	3.118392	-0.756117
H	-3.137190	3.066759	0.022132
H	-1.669584	3.077682	1.018079
C	2.091209	-2.685439	0.127204
H	1.514104	-3.127342	-0.700282
H	3.119906	-3.072179	0.047498
H	1.671039	-3.060760	1.070600
C	1.517948	-0.738038	2.574309
H	0.708938	-1.481336	2.636121
H	2.419357	-1.197339	3.009315
H	1.237635	0.110796	3.210495
C	2.729376	-0.967954	-2.405548
H	3.661704	-1.553476	-2.377773
H	1.932130	-1.640992	-2.759344
H	2.856880	-0.178612	-3.157500
C	2.479272	2.158933	-1.576652
H	3.443826	2.650656	-1.370873
H	2.464506	1.890968	-2.641302
H	1.689884	2.907760	-1.411224
C	1.714576	2.307441	1.435903
H	1.559407	2.120714	2.506926
H	2.606967	2.944458	1.339302
H	0.856193	2.894140	1.069505

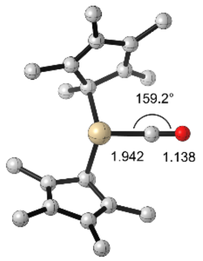

M06-2X-D3/def2-svp
Thermal correction to Gibbs Energy = 0.396125
Thermal correction to Enthalpy = 0.483414
Sum of electronic and thermal Free Energies = −1181.384600
Sum of electronic and thermal Enthalpies = −1181.297311

Atomic Type	Coordinates (Angstroms) X	Y	Z
C	-2.517539	-0.646844	0.602568
C	-1.548696	0.471791	0.820208
C	-2.046428	1.550564	-0.097175
C	-3.057160	1.051111	-0.859597
C	-3.347482	-0.319671	-0.429149
C	2.495476	-1.176922	-0.085471
C	3.298752	-0.371412	-0.842836

C	3.163677	1.002785	-0.391479
C	2.275223	1.047603	0.648218
C	1.717793	-0.328231	0.865855
Si	0.078322	-0.105659	-0.255210
C	-0.415255	-1.978546	-0.399076
O	-0.700236	-2.969586	-0.880776
C	1.604769	-0.817647	2.303685
H	2.610926	-0.952022	2.733554
H	1.065794	-0.099239	2.933375
H	1.072812	-1.777998	2.371353
C	1.919564	2.242606	1.481565
H	1.919245	1.993290	2.554477
H	2.646607	3.051060	1.331368
H	0.926745	2.653729	1.242517
C	3.895140	2.140796	-1.033946
H	3.569294	2.276144	-2.077644
H	3.727390	3.089215	-0.508260
H	4.979568	1.952443	-1.058911
C	4.190108	-0.790605	-1.970331
H	3.882159	-0.316673	-2.915874
H	5.233923	-0.491140	-1.787067
H	4.170023	-1.878305	-2.117956
C	2.441096	-2.678229	-0.142824
H	2.051313	-3.055881	-1.101575
H	3.448118	-3.104009	-0.016045
H	1.813324	-3.093616	0.657721
C	-1.409269	2.900855	-0.180168
H	-0.389948	2.839387	-0.602547
H	-1.986672	3.580235	-0.819861
H	-1.325379	3.367227	0.815063
C	-1.344806	0.865981	2.280602
H	-0.534611	1.595300	2.415234
H	-2.273102	1.315588	2.669822
H	-1.111796	-0.009431	2.901854
C	-3.784504	1.711559	-1.989533
H	-4.874331	1.675600	-1.836987
H	-3.493795	2.763173	-2.105792
H	-3.576780	1.200405	-2.943330
C	-4.432855	-1.153337	-1.036681
H	-5.424854	-0.712693	-0.847390
H	-4.316839	-1.219375	-2.129623
H	-4.438193	-2.174386	-0.633718
C	-2.597780	-1.847318	1.497957
H	-1.608069	-2.137312	1.884858
H	-3.232670	-1.636848	2.374643
H	-3.023759	-2.719279	0.981548

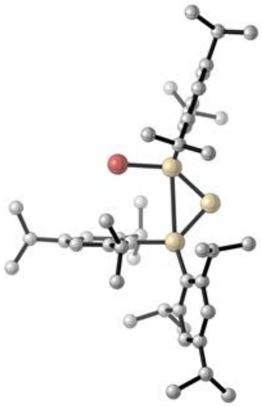

BP86-D3(BJ)/def2-svp
Thermal correction to Gibbs Energy = 0.897582
Thermal correction to Enthalpy = 1.064530
Sum of electronic and thermal Free Energies = −5196.902919
Sum of electronic and thermal Enthalpies = −5196.735972

Atomic Type	Coordinates (Angstroms) X	Y	Z
Br	-1.676752	-1.991327	0.763390
Si	1.208055	0.304782	-0.979111
Si	-0.640249	1.058173	-2.042186
Si	-1.893728	-0.239787	-0.693653
C	1.506907	-1.359777	-0.122812
C	1.466124	-2.581665	-0.849111
C	1.652888	-3.791034	-0.155385
H	1.613192	-4.744955	-0.707376
C	1.877598	-3.828168	1.231590
C	1.918245	-2.608177	1.933809
H	2.078668	-2.617994	3.024105
C	1.724719	-1.379252	1.283862
C	1.197781	-2.617269	-2.352613
H	1.235813	-1.560441	-2.714132
C	-0.218846	-3.130490	-2.659646
H	-0.384049	-4.140906	-2.230848
H	-0.390853	-3.183288	-3.755207
H	-0.980461	-2.452076	-2.223757
C	2.265849	-3.408775	-3.128212
H	3.286942	-3.037650	-2.908656
H	2.095453	-3.325503	-4.221654
H	2.240505	-4.488395	-2.870562
C	2.023307	-5.156874	1.960597
C	0.800970	-5.416800	2.864388
H	0.867955	-6.413516	3.349356
H	-0.142091	-5.374232	2.282450
H	0.731297	-4.653014	3.667727
C	1.715313	-0.087401	2.096252
H	1.344425	0.716292	1.418099
C	0.723065	-0.136326	3.270737

H	-0.289323	-0.421184	2.921457
H	0.658535	0.855335	3.765546
H	1.036539	-0.871335	4.041688
C	3.132720	0.310218	2.542400
H	3.565943	-0.463808	3.211102
H	3.120297	1.272038	3.096461
H	3.806254	0.429286	1.670439
C	2.718762	1.440001	-0.723317
C	4.005670	0.971773	-1.119968
C	5.148015	1.733639	-0.821307
H	6.137496	1.353776	-1.124009
C	5.059289	2.966280	-0.148841
C	3.783199	3.428234	0.215489
H	3.706277	4.395461	0.738229
C	2.612038	2.694430	-0.053441
C	4.175624	-0.352574	-1.858497
H	3.153138	-0.728114	-2.093286
C	4.857886	-1.408650	-0.971651
H	5.882346	-1.085788	-0.689472
H	4.942873	-2.379828	-1.502351
H	4.280009	-1.579447	-0.041733
C	4.889801	-0.177443	-3.210352
H	4.374114	0.575099	-3.840947
H	4.914358	-1.137972	-3.766217
H	5.940341	0.155960	-3.076078
C	6.307606	3.770956	0.187359
H	5.964539	4.707168	0.681705
C	7.202661	3.014272	1.188778
H	7.584065	2.068622	0.748445
H	6.642152	2.752429	2.109342
H	8.081161	3.627937	1.479510
C	7.089225	4.169691	-1.079074
H	7.960727	4.807604	-0.821802
H	6.446870	4.730425	-1.788318
H	7.476143	3.275154	-1.611579
C	1.265077	3.271775	0.376401
H	0.525010	2.437491	0.358074
C	0.770370	4.312130	-0.644956
H	1.473201	5.169751	-0.711853
H	-0.228812	4.701750	-0.358066
H	0.680846	3.861172	-1.654540
C	1.278876	3.837217	1.806014
H	1.647237	3.087032	2.535202
H	0.256071	4.139765	2.109426
H	1.922880	4.737150	1.893104
C	-3.662982	0.351472	-0.374238
C	-4.704016	-0.089946	-1.228922
C	-6.010082	0.388319	-1.014927
H	-6.829278	0.056382	-1.674707
C	-6.305193	1.283750	0.030340
C	-5.255743	1.702081	0.873374

H	-5.473595	2.398682	1.699919
C	-3.937552	1.250773	0.687242
C	-4.391653	-1.043278	-2.377777
H	-3.352480	-1.426646	-2.196470
C	-4.354570	-0.294460	-3.721784
H	-5.348499	0.142040	-3.956879
H	-4.073015	-0.976687	-4.551175
H	-3.617924	0.534648	-3.689962
C	-5.316224	-2.269802	-2.410007
H	-5.304679	-2.803062	-1.437872
H	-4.997845	-2.980223	-3.200930
H	-6.366158	-1.980730	-2.628010
C	-7.728215	1.783687	0.247246
C	-7.822912	3.312398	0.076767
H	-7.445109	3.630357	-0.916332
H	-7.223086	3.840112	0.848309
C	-8.282743	1.333173	1.612832
H	-8.235017	0.230488	1.721965
H	-9.340247	1.648717	1.735418
H	-7.701464	1.778181	2.448098
C	-2.821556	1.706528	1.621108
H	-1.870770	1.262950	1.228149
C	-3.017600	1.161706	3.045715
H	-3.947782	1.561110	3.502947
H	-2.167250	1.447616	3.699148
H	-3.090607	0.055370	3.034603
C	-2.635284	3.231281	1.588634
H	-2.412329	3.577500	0.558665
H	-1.798782	3.539663	2.249253
H	-3.548224	3.759079	1.936293
H	2.042175	-5.952050	1.182138
C	3.341575	-5.244423	2.751694
H	4.217930	-5.070448	2.094345
H	3.454439	-6.243483	3.222516
H	3.375990	-4.488762	3.564811
H	-8.357764	1.316721	-0.542803
H	-8.873438	3.658109	0.175459

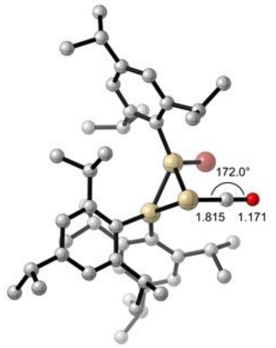

BP86-D3(BJ)/def2-svp

Thermal correction to Gibbs Energy = 0.911722
Thermal correction to Enthalpy = 1.076610
Sum of electronic and thermal Free Energies = −5310.169438
Sum of electronic and thermal Enthalpies = −5310.004550

Atomic Type	Coordinates (Angstroms) X	Y	Z
Br	1.045924	-3.466870	-0.102178
Si	-0.653799	-0.279681	0.501885
Si	0.458927	-0.621303	2.663275
Si	1.415907	-1.301580	0.527903
O	0.259721	-3.538317	3.236094
C	-2.158641	-1.033744	-0.388450
C	-2.948739	-2.101080	0.123437
C	-4.211218	-2.355678	-0.445008
H	-4.831680	-3.167539	-0.030220
C	-4.710702	-1.611536	-1.526235
C	-3.877316	-0.624051	-2.081195
H	-4.222795	-0.061346	-2.963628
C	-2.609742	-0.331891	-1.545919
C	-2.461870	-2.999878	1.251362
H	-1.408831	-2.716363	1.432053
C	-2.450223	-4.480469	0.832889
H	-3.477719	-4.878067	0.691444
H	-1.954044	-5.093539	1.613122
H	-1.893692	-4.617943	-0.115654
C	-3.235427	-2.789696	2.564410
H	-3.153003	-1.744205	2.922281
H	-2.835417	-3.450844	3.360862
H	-4.314570	-3.021104	2.439721
C	-6.107318	-1.865659	-2.076254
C	-6.083928	-2.235750	-3.571144
H	-7.104481	-2.479480	-3.933864
H	-5.431139	-3.112606	-3.758473
H	-5.703857	-1.395099	-4.189431
C	-1.742113	0.693102	-2.273164
H	-0.771892	0.761636	-1.730024
C	-1.415138	0.201292	-3.695862
H	-0.918100	-0.789798	-3.671792
H	-0.745710	0.918224	-4.213073
H	-2.336313	0.100207	-4.306884
C	-2.368526	2.097836	-2.291483
H	-3.305599	2.106484	-2.887733
H	-1.672362	2.831108	-2.748587
H	-2.608289	2.447250	-1.268920
C	-0.932687	1.597726	0.724602
C	-2.148446	1.968992	1.373415
C	-2.525506	3.322811	1.423020
H	-3.478767	3.589407	1.907864
C	-1.722530	4.337708	0.874713

C	-0.496586	3.963530	0.300221
H	0.155837	4.754613	-0.101791
C	-0.081697	2.620420	0.218755
C	-3.050203	0.946741	2.056304
H	-2.587748	-0.051729	1.909393
C	-4.455455	0.882853	1.436767
H	-5.007826	1.835270	1.583969
H	-5.050473	0.071365	1.904476
H	-4.400784	0.680272	0.348906
C	-3.085023	1.179795	3.577614
H	-2.060112	1.157591	4.002854
H	-3.685985	0.393492	4.081406
H	-3.536510	2.162480	3.829447
C	-2.168216	5.793401	0.890441
H	-1.329822	6.392213	0.469412
C	-3.394587	5.999896	-0.021105
H	-4.268255	5.424269	0.351754
H	-3.186590	5.658476	-1.055687
H	-3.686975	7.070432	-0.060168
C	-2.435362	6.301409	2.319734
H	-2.696913	7.380340	2.313406
H	-1.546254	6.164232	2.968409
H	-3.281515	5.757149	2.789962
C	1.303349	2.307245	-0.328747
H	1.275092	1.261017	-0.707085
C	2.330977	2.353518	0.819040
H	2.371904	3.373053	1.257728
H	3.342635	2.078598	0.459959
H	2.055591	1.652031	1.632935
C	1.751655	3.211714	-1.484984
H	1.004518	3.234320	-2.305002
H	2.714565	2.846168	-1.895571
H	1.919617	4.256968	-1.150875
C	3.123082	-0.750709	-0.050076
C	4.181366	-0.590984	0.885112
C	5.341253	0.104803	0.489303
H	6.156187	0.255048	1.216949
C	5.490492	0.622283	-0.806971
C	4.474481	0.361006	-1.749023
H	4.606904	0.702690	-2.786944
C	3.304308	-0.334433	-1.405060
C	4.124174	-1.190202	2.286337
H	3.144846	-1.708147	2.378847
C	4.179243	-0.118739	3.387591
H	5.130497	0.453311	3.351111
H	4.101902	-0.583742	4.392379
H	3.343243	0.602201	3.282453
C	5.214551	-2.263949	2.465327
H	5.135593	-3.044656	1.681665
H	5.116176	-2.756365	3.455360
H	6.233476	-1.826022	2.406633

C	6.716633	1.439921	-1.188298
C	6.321104	2.893474	-1.517465
H	5.771077	3.360324	-0.674971
H	5.662487	2.934910	-2.410940
C	7.507157	0.793880	-2.341854
H	7.801215	-0.246595	-2.094944
H	8.429294	1.371921	-2.560626
H	6.905733	0.761274	-3.274884
C	2.316482	-0.750161	-2.491946
H	1.322812	-0.895534	-2.003767
C	2.753802	-2.115742	-3.066327
H	3.728916	-2.017525	-3.589064
H	2.005128	-2.491740	-3.794610
H	2.864839	-2.872615	-2.266219
C	2.137858	0.270883	-3.623332
H	1.781497	1.248347	-3.247384
H	1.401563	-0.104079	-4.360362
H	3.085520	0.440940	-4.175812
C	0.308548	-2.412585	2.917671
H	-6.520223	-2.735792	-1.518705
C	-7.027170	-0.657728	-1.806983
H	-7.062484	-0.414437	-0.725498
H	-8.062958	-0.860350	-2.152104
H	-6.663463	0.245695	-2.341118
H	7.379774	1.463783	-0.295036
H	7.218232	3.511588	-1.731805

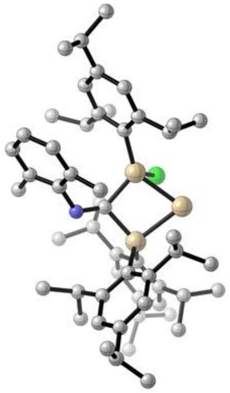

BP86-D3(BJ)/def2-svp
Thermal correction to Gibbs Energy = 1.047479
Thermal correction to Enthalpy = 1.227351
Sum of electronic and thermal Free Energies = −3485.641581
Sum of electronic and thermal Enthalpies = −3485.461708

Atomic Type	Coordinates (Angstroms) X	Y	Z
Si	0.147098	0.188158	-2.611417
Cl	-0.568222	-2.257447	-2.073382

Si	-1.523343	-0.503494	-1.066863
Si	1.264164	-0.057234	-0.466992
N	-0.627730	-0.035511	1.691244
C	-0.397719	-0.405265	0.475364
C	-1.936313	0.171240	2.163327
C	-2.426257	-0.669325	3.196431
C	-3.735003	-0.458176	3.667756
H	-4.127289	-1.114152	4.462171
C	-4.536298	0.569306	3.144595
H	-5.560103	0.716847	3.521610
C	-4.013387	1.430988	2.168569
H	-4.623299	2.263374	1.782544
C	-2.705505	1.264960	1.678002
C	-2.090965	2.264043	0.729769
H	-1.071858	2.550738	1.063653
H	-2.718190	3.173406	0.652338
H	-1.968557	1.877552	-0.306776
C	-1.543592	-1.767806	3.725777
H	-0.537931	-1.373202	3.980174
H	-1.376489	-2.555824	2.961100
H	-1.983398	-2.249422	4.620970
C	-4.160004	0.455302	-1.549800
C	-5.518775	0.568533	-1.213315
H	-6.107714	1.397735	-1.633111
C	-6.147258	-0.346682	-0.347759
C	-5.393613	-1.422664	0.150350
H	-5.890432	-2.153596	0.806291
C	-4.031367	-1.581858	-0.158841
C	-3.395811	-0.609522	-0.984362
C	-3.534211	1.417110	-2.560220
H	-2.477323	1.580504	-2.250462
C	-4.200391	2.797338	-2.624625
H	-5.223890	2.747111	-3.052477
H	-3.611586	3.476015	-3.275394
H	-4.273270	3.261862	-1.619957
C	-3.473511	0.745948	-3.946791
H	-2.903018	-0.204518	-3.901709
H	-2.973577	1.405977	-4.686019
H	-4.494865	0.512881	-4.315523
C	-7.609209	-0.175623	0.041281
H	-7.866865	-1.027399	0.709323
C	-8.538588	-0.244093	-1.185789
H	-9.603748	-0.179441	-0.879363
H	-8.396237	-1.190641	-1.746075
H	-8.341505	0.594381	-1.886909
C	-7.822492	1.125094	0.839916
H	-7.169075	1.152809	1.734688
H	-8.876903	1.217745	1.174908
H	-7.585642	2.017418	0.222603
C	-3.278564	-2.826037	0.300932
H	-2.217301	-2.524327	0.471627

C	-3.806681	-3.448532	1.601168
H	-3.913837	-2.693340	2.401899
H	-3.116526	-4.242270	1.953109
H	-4.795463	-3.928874	1.444969
C	-3.296144	-3.881878	-0.827569
H	-4.335040	-4.235972	-0.996525
H	-2.670845	-4.758027	-0.556229
H	-2.916914	-3.471486	-1.782394
C	2.642442	-1.369589	-0.469401
C	3.850439	-1.016098	-1.142378
C	4.963415	-1.872191	-1.087523
H	5.891808	-1.572443	-1.600937
C	4.922084	-3.094366	-0.393169
C	3.713830	-3.452619	0.224348
H	3.663567	-4.419497	0.752769
C	2.572136	-2.625484	0.198527
C	3.973810	0.276193	-1.944232
H	2.991273	0.789728	-1.891140
C	4.224348	-0.012072	-3.434842
H	5.193722	-0.529420	-3.594288
H	4.248578	0.931484	-4.020389
H	3.422044	-0.655547	-3.851059
C	5.006204	1.247686	-1.348373
H	4.758327	1.491292	-0.296034
H	5.024474	2.198369	-1.921271
H	6.029923	0.817451	-1.372322
C	6.145749	-3.996624	-0.308117
H	5.839222	-4.903437	0.259844
C	7.283255	-3.314670	0.477555
H	8.153158	-3.995412	0.591689
H	6.944996	-3.010249	1.489030
H	7.637115	-2.401070	-0.045673
C	6.617618	-4.454375	-1.701403
H	6.954492	-3.592161	-2.315107
H	5.801475	-4.961408	-2.255456
H	7.471605	-5.159286	-1.619417
C	1.319235	-3.139600	0.907254
H	0.515608	-2.392450	0.748033
C	0.824661	-4.467076	0.302839
H	0.673658	-4.376463	-0.790323
H	-0.144071	-4.761791	0.759649
H	1.543974	-5.293577	0.484284
C	1.537141	-3.274803	2.425238
H	2.369483	-3.973908	2.651663
H	0.625968	-3.669164	2.919864
H	1.779069	-2.299155	2.889593
C	1.889834	1.606086	0.245341
C	1.740731	2.849419	-0.435630
C	2.392667	3.995495	0.057335
H	2.285213	4.947426	-0.487389
C	3.176932	3.962314	1.224846

C	3.249445	2.750750	1.929848
H	3.822151	2.720320	2.871969
C	2.608736	1.582060	1.473939
C	0.895279	3.022689	-1.698297
H	0.268278	2.087436	-1.796496
C	-0.127708	4.166390	-1.588561
H	-0.706546	4.107613	-0.647770
H	-0.836699	4.134256	-2.441470
H	0.376411	5.155027	-1.614420
C	1.750966	3.170088	-2.967143
H	1.113035	3.265793	-3.869638
H	2.419769	2.300442	-3.115072
H	2.386843	4.076954	-2.891790
C	3.906975	5.204495	1.717962
H	4.432301	4.917378	2.656045
C	2.927729	6.343669	2.060847
H	2.379023	6.687577	1.158405
H	3.468228	7.219887	2.476680
H	2.174880	6.015304	2.806183
C	4.974602	5.662206	0.705044
H	5.689665	4.844927	0.479466
H	5.548862	6.528515	1.095772
H	4.508543	5.973765	-0.253886
C	2.679578	0.327873	2.339781
H	2.037252	-0.437792	1.858503
C	4.103818	-0.246950	2.428051
H	4.505692	-0.482811	1.423094
H	4.109639	-1.184418	3.022184
H	4.796395	0.469276	2.919385
C	2.067888	0.578335	3.730127
H	2.653115	1.321779	4.311559
H	2.050918	-0.363644	4.318803
H	1.026186	0.940611	3.630524

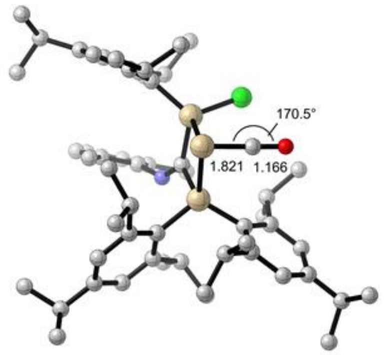

BP86-D3(BJ)/def2-svp
Thermal correction to Gibbs Energy = 1.055849
Thermal correction to Enthalpy = 1.239022
Sum of electronic and thermal Free Energies = −3598.903923
Sum of electronic and thermal Enthalpies = −3598.720750

Atomic Type	Coordinates (Angstroms) X	Y	Z
Si	0.233889	-0.925521	-2.490798
Cl	-0.750042	-3.842182	-0.355794
Si	-1.248834	-1.818296	-0.791434
Si	1.269038	-0.118158	-0.438820
N	-0.606411	-0.161022	1.640367
C	-0.306561	-0.745125	0.550149
C	-1.721190	0.013552	2.447955
C	-1.727828	-0.607095	3.730867
C	-2.708817	-0.223759	4.659944
H	-2.715423	-0.705632	5.651062
C	-3.675465	0.742911	4.337953
H	-4.436446	1.035896	5.077096
C	-3.663925	1.331886	3.062654
H	-4.412373	2.098473	2.803960
C	-2.696814	0.992635	2.098291
C	-2.637767	1.676632	0.759159
H	-1.616236	2.062933	0.555381
H	-3.348467	2.523818	0.712733
H	-2.896738	0.979296	-0.065246
C	-0.687975	-1.649267	4.034021
H	0.329474	-1.244557	3.856815
H	-0.788068	-2.532349	3.364700
H	-0.752432	-2.001674	5.081630
C	-3.670510	-0.837751	-2.080123
C	-4.927601	-0.213129	-1.961248
H	-5.335969	0.326240	-2.824886
C	-5.677136	-0.260932	-0.774119
C	-5.187741	-1.051159	0.279621
H	-5.804077	-1.164101	1.183864
C	-3.939859	-1.691005	0.215716
C	-3.115009	-1.506500	-0.940113
C	-2.979516	-0.868078	-3.443395
H	-1.955964	-0.448606	-3.315420
C	-3.649740	-0.021457	-4.532047
H	-4.657898	-0.405189	-4.796771
H	-3.035065	-0.045312	-5.454952
H	-3.757005	1.039544	-4.227458
C	-2.797766	-2.323092	-3.918232
H	-2.225982	-2.927930	-3.183895
H	-2.246788	-2.356542	-4.881044
H	-3.782458	-2.817258	-4.057311
C	-6.966315	0.526853	-0.584093
H	-7.628943	-0.095952	0.059016
C	-7.720055	0.826391	-1.887668
H	-8.696961	1.304640	-1.669669
H	-7.912230	-0.096913	-2.471771
H	-7.152820	1.527653	-2.535741

C	-6.658521	1.825572	0.193757
H	-6.152775	1.604702	1.155312
H	-7.588147	2.393140	0.409479
H	-5.983025	2.479749	-0.397317
C	-3.554718	-2.674005	1.316302
H	-2.457308	-2.587071	1.488518
C	-4.266192	-2.439780	2.655092
H	-4.236606	-1.381582	2.967987
H	-3.787674	-3.043573	3.452172
H	-5.329340	-2.757003	2.601720
C	-3.852201	-4.109478	0.823000
H	-4.946359	-4.235576	0.680806
H	-3.511598	-4.856942	1.569687
H	-3.351829	-4.331071	-0.137531
C	3.027174	-0.818066	-0.235968
C	4.034840	-0.196545	-1.033323
C	5.380952	-0.562224	-0.867347
H	6.148123	-0.060448	-1.479557
C	5.771241	-1.549035	0.055992
C	4.762683	-2.190992	0.790570
H	5.054190	-2.988291	1.494370
C	3.399395	-1.856307	0.659980
C	3.695338	0.855240	-2.085904
H	2.590877	0.966065	-2.102021
C	4.089343	0.389784	-3.498782
H	5.188475	0.269872	-3.599805
H	3.758256	1.127253	-4.259648
H	3.620249	-0.586644	-3.738537
C	4.270741	2.237407	-1.735466
H	3.901461	2.580061	-0.748294
H	3.969210	2.992240	-2.491395
H	5.380597	2.215499	-1.702644
C	7.236949	-1.913300	0.249142
H	7.269494	-2.730457	1.004005
C	8.035032	-0.721790	0.814303
H	9.089735	-1.007110	1.012157
H	7.590749	-0.354981	1.762044
H	8.045149	0.127603	0.098680
C	7.867593	-2.450740	-1.049776
H	7.879475	-1.673108	-1.842652
H	7.301478	-3.320284	-1.441490
H	8.917519	-2.768631	-0.878522
C	2.391570	-2.653123	1.483128
H	1.379443	-2.251103	1.262322
C	2.393596	-4.138143	1.072512
H	2.224659	-4.256747	-0.014465
H	1.592891	-4.689609	1.606878
H	3.363441	-4.618465	1.322008
C	2.641596	-2.508364	2.995448
H	3.643280	-2.897670	3.273779
H	1.889834	-3.081970	3.574415

H	2.593657	-1.450087	3.319576
C	1.243181	1.770184	-0.107806
C	0.804582	2.770824	-1.027222
C	0.755654	4.116129	-0.619523
H	0.404277	4.874346	-1.335829
C	1.140052	4.524156	0.669249
C	1.589596	3.536484	1.556640
H	1.904844	3.840724	2.567763
C	1.642703	2.174231	1.201595
C	0.341500	2.441862	-2.444698
H	0.843034	1.497766	-2.747997
C	-1.172214	2.169639	-2.472897
H	-1.448810	1.343039	-1.787075
H	-1.510447	1.896559	-3.493534
H	-1.741680	3.063452	-2.142235
C	0.737114	3.490805	-3.496434
H	0.521543	3.107032	-4.515013
H	1.818240	3.731440	-3.439910
H	0.171945	4.439429	-3.380745
C	1.059048	5.984490	1.092174
H	1.462500	6.042997	2.127733
C	-0.403872	6.469004	1.132363
H	-0.861736	6.438539	0.120794
H	-0.467082	7.515289	1.498849
H	-1.019170	5.830016	1.798096
C	1.929380	6.888022	0.198105
H	2.984515	6.546208	0.187394
H	1.908863	7.938631	0.556609
H	1.565510	6.886622	-0.851257
C	2.183938	1.193688	2.241017
H	1.907891	0.170356	1.912107
C	3.721744	1.263576	2.313814
H	4.184645	1.075018	1.325985
H	4.116516	0.504616	3.021191
H	4.051400	2.264432	2.664831
C	1.551336	1.384005	3.630175
H	1.861936	2.341451	4.098194
H	1.879124	0.573609	4.314673
H	0.445993	1.364379	3.574327
C	1.312886	-2.379471	-2.296280
O	2.045380	-3.284470	-2.358472

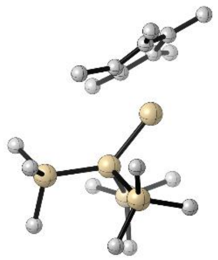

BP86-D3(BJ)/def2-svp
Thermal correction to Gibbs Energy = 0.468460
Thermal correction to Enthalpy = 0.583886
Sum of electronic and thermal Free Energies = −2195.672292
Sum of electronic and thermal Enthalpies = −2195.556865

Atomic Type	Coordinates (Angstroms) X	Y	Z
Si	-1.345421	-0.004763	0.751781
Si	0.983861	-0.002524	0.043479
Si	1.678484	2.191289	0.552368
Si	2.177846	-0.716231	-1.859669
Si	1.736632	-1.349571	1.829075
C	-2.395146	-0.845481	-0.928660
C	-3.550502	-1.208631	-0.122789
C	-4.246899	-0.027293	0.241304
C	-3.536426	1.090731	-0.285654
C	-2.398680	0.615018	-1.044032
C	-1.641488	-1.800247	-1.816559
C	-3.903243	-2.617941	0.245579
C	-5.415637	0.075376	1.178883
C	-3.896878	2.527542	-0.059438
C	-1.615015	1.420818	-2.044763
C	0.429438	3.000363	1.739564
C	1.754437	3.233988	-1.040435
C	3.400638	2.227852	1.360893
C	2.173278	-2.617578	-1.967339
C	3.986049	-0.140269	-1.694149
C	1.530783	-0.014038	-3.509161
C	1.305155	-0.523899	3.488658
C	0.785477	-2.998542	1.722780
C	3.604134	-1.711948	1.788816
H	-1.392109	-2.743243	-1.289173
H	-0.689844	-1.358189	-2.164678
H	-2.242683	-2.065671	-2.714502
H	-2.996155	-3.224283	0.442490
H	-4.453607	-3.119498	-0.581988
H	-4.544054	-2.665582	1.147481
H	-5.913459	-0.902895	1.327378
H	-6.182929	0.782712	0.799828
H	-5.102423	0.443597	2.181864
H	-4.141082	2.726042	1.004582
H	-3.076941	3.210911	-0.354980
H	-4.794636	2.808747	-0.653979
H	-0.594109	1.017586	-2.181192
H	-2.118301	1.421399	-3.036823
H	-1.509327	2.476496	-1.723318
H	0.744006	4.032880	2.005892
H	0.332516	2.415157	2.676926
H	-0.580036	3.055411	1.279182

H	2.044586	4.282124	-0.810106
H	0.772289	3.255018	-1.555414
H	2.500525	2.824514	-1.752841
H	3.722928	3.273976	1.555568
H	3.399941	1.685225	2.328470
H	4.156827	1.749837	0.705224
H	1.143508	-3.020507	-2.049945
H	2.639097	-3.064649	-1.065061
H	2.749400	-2.960613	-2.853677
H	4.435780	-0.490740	-0.743683
H	4.056741	0.966830	-1.715640
H	4.597407	-0.536538	-2.533986
H	0.508528	-0.367437	-3.754330
H	1.512831	1.095175	-3.491936
H	2.202187	-0.330414	-4.336618
H	0.219356	-0.296680	3.538831
H	1.857877	0.428664	3.625694
H	1.558749	-1.192264	4.339974
H	-0.307713	-2.813098	1.804864
H	1.075598	-3.697210	2.537116
H	0.970356	-3.502670	0.751593
H	3.898645	-2.333300	2.662407
H	3.889202	-2.263538	0.869244
H	4.194042	-0.773015	1.819792

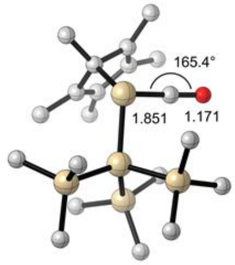

BP86-D3(BJ)/def2-svp
Thermal correction to Gibbs Energy = 0.478907
Thermal correction to Enthalpy = 0.594723
Sum of electronic and thermal Free Energies = −2308.920772
Sum of electronic and thermal Enthalpies = −2308.804957

Atomic Type	Coordinates (Angstroms) X	Y	Z
Si	-0.632468	-0.427833	-1.568916
Si	1.129257	0.055140	-0.032957
Si	2.401472	-1.954790	-0.173455
Si	0.687647	0.353162	2.258997
Si	2.523616	1.686799	-1.012724
O	-1.423197	-2.944448	-0.141377
C	-2.382522	0.594523	-1.418478

C	-3.345716	-0.522662	-1.060487
C	-3.785355	-0.316174	0.239771
C	-3.209049	0.925463	0.752441
C	-2.385990	1.478928	-0.216117
C	-2.631194	1.294134	-2.765425
C	-3.835167	-1.537997	-2.051773
C	-4.678154	-1.209817	1.048393
C	-3.517970	1.470952	2.116693
C	-1.624038	2.762201	-0.157548
C	-1.228638	-1.877577	-0.583617
C	2.291302	-2.684215	-1.923840
C	1.791865	-3.273110	1.054863
C	4.226611	-1.600491	0.236396
C	0.189656	2.117349	2.755004
C	2.287831	-0.041100	3.216462
C	-0.676639	-0.871922	2.747937
C	3.297876	0.969279	-2.593598
C	1.613170	3.279189	-1.514591
C	3.875057	2.160833	0.238983
H	-2.626270	0.570932	-3.606162
H	-1.851000	2.052862	-2.982902
H	-3.616072	1.809743	-2.753444
H	-3.025095	-1.890126	-2.725863
H	-4.627441	-1.108692	-2.706332
H	-4.267238	-2.427483	-1.551029
H	-4.979274	-2.115493	0.486905
H	-5.607236	-0.685106	1.362288
H	-4.175616	-1.544830	1.982448
H	-3.085089	0.841624	2.924777
H	-3.123973	2.497296	2.252752
H	-4.613922	1.506387	2.297093
H	-0.530922	2.577117	-0.082175
H	-1.776923	3.376131	-1.071157
H	-1.914075	3.379665	0.714493
H	2.937132	-3.585102	-2.007550
H	2.613534	-1.951038	-2.690234
H	1.249847	-2.980644	-2.166119
H	2.420127	-4.184739	0.954998
H	0.740008	-3.562365	0.862571
H	1.866910	-2.925811	2.105254
H	4.804458	-2.549916	0.222298
H	4.689937	-0.911151	-0.498086
H	4.334894	-1.150479	1.244236
H	-0.820429	2.375362	2.388115
H	0.904079	2.867710	2.357182
H	0.185135	2.204554	3.863009
H	3.105537	0.648095	2.919756
H	2.635629	-1.078402	3.038073
H	2.120232	0.078952	4.308765
H	-1.571871	-0.710802	2.113674
H	-0.343886	-1.918460	2.598566

H	-0.967403	-0.747820	3.813103
H	2.504707	0.635543	-3.295223
H	3.947756	0.097283	-2.378467
H	3.913447	1.737115	-3.109779
H	0.773627	3.069267	-2.208966
H	2.323348	3.958486	-2.034265
H	1.205999	3.821637	-0.637306
H	4.585034	2.890904	-0.206146
H	3.424066	2.630319	1.138370
H	4.452920	1.275650	0.571459

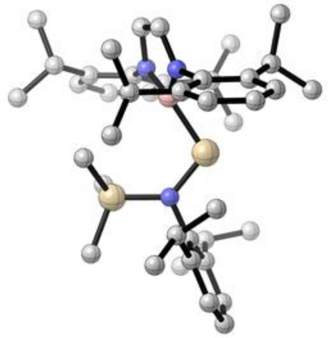

BP86-D3(BJ)/def2-svp
Thermal correction to Gibbs Energy = 0.832056
Thermal correction to Enthalpy = 0.975528
Sum of electronic and thermal Free Energies = −2365.912050
Sum of electronic and thermal Enthalpies = −2365.768579

Atomic Type	Coordinates (Angstroms) X	Y	Z
Si	-0.633289	-0.225859	0.960457
Si	-0.202346	-1.756862	-1.855480
N	2.294188	1.027295	0.017465
N	0.500188	2.422556	0.198741
N	-1.052542	-1.395232	-0.296754
C	2.730827	2.358435	-0.083631
H	3.790909	2.606354	-0.201692
C	1.652563	3.195937	0.019625
H	1.615971	4.290738	-0.013591
C	3.218460	-0.060360	0.027481
C	3.958741	-0.376096	-1.145991
C	4.833775	-1.478717	-1.104269
H	5.408291	-1.743651	-2.005423
C	4.969951	-2.257213	0.052997
H	5.645152	-3.127143	0.057480
C	4.251990	-1.917608	1.206615
H	4.379171	-2.516694	2.122295
C	3.383156	-0.808100	1.225030
C	2.695787	-0.415974	2.528449
H	2.057024	0.466044	2.317707

C	3.726625	0.012907	3.590101
H	4.396613	-0.827122	3.871475
H	3.214611	0.358826	4.512434
H	4.362626	0.841470	3.217250
C	1.776812	-1.533254	3.043200
H	1.007435	-1.796725	2.285618
H	1.237048	-1.219287	3.959657
H	2.341484	-2.460939	3.275770
C	3.840209	0.445363	-2.426703
H	2.950734	1.099694	-2.321098
C	5.073573	1.354138	-2.611396
H	5.996124	0.747103	-2.729766
H	5.228432	2.022801	-1.740999
H	4.964761	1.988980	-3.515736
C	3.628970	-0.430567	-3.674951
H	4.526449	-1.041584	-3.907001
H	3.425365	0.203873	-4.562431
H	2.774485	-1.121450	-3.543203
C	-0.781833	2.999062	0.469433
C	-1.579643	3.467851	-0.605283
C	-2.823637	4.056194	-0.302603
H	-3.454588	4.439020	-1.120000
C	-3.275869	4.145258	1.020595
H	-4.257446	4.595039	1.237813
C	-2.482689	3.657326	2.068872
H	-2.846770	3.730994	3.104715
C	-1.217625	3.089956	1.821129
C	-0.313133	2.665395	2.973510
H	0.414618	1.933972	2.563575
C	0.498117	3.875174	3.482369
H	1.196619	3.568007	4.289074
H	-0.174309	4.659965	3.889823
H	1.096739	4.328970	2.666842
C	-1.068125	1.963343	4.111514
H	-1.644301	1.099719	3.721137
H	-1.764446	2.649062	4.639433
H	-0.349968	1.583552	4.867732
C	-1.094110	3.371393	-2.046409
H	-0.241503	2.662335	-2.052446
C	-2.169773	2.810862	-2.992397
H	-1.751040	2.654085	-4.007935
H	-3.034692	3.499496	-3.095569
H	-2.556657	1.838725	-2.627216
C	-0.571650	4.734648	-2.542696
H	0.247021	5.112604	-1.897851
H	-1.380645	5.495963	-2.539622
H	-0.181110	4.654844	-3.579077
C	-2.212446	-2.218308	-0.030703
C	-3.480380	-1.784373	-0.514498
C	-4.604750	-2.602314	-0.299664
H	-5.589085	-2.275521	-0.668176

C	-4.492879	-3.824977	0.378446
H	-5.382451	-4.455466	0.534034
C	-3.245326	-4.233632	0.863516
H	-3.160460	-5.187277	1.409059
C	-2.090142	-3.447103	0.671771
C	-0.763870	-3.918779	1.254918
H	0.012035	-3.195222	0.933434
C	-0.353728	-5.304226	0.724302
H	-0.337746	-5.324195	-0.385014
H	-1.056620	-6.096216	1.059103
H	0.658214	-5.580603	1.088042
C	-0.812624	-3.892118	2.794808
H	-1.582012	-4.592339	3.183615
H	-1.065027	-2.875948	3.160626
H	0.165525	-4.186990	3.229088
C	-3.628865	-0.424431	-1.186995
H	-2.642520	-0.174428	-1.632340
C	-4.670135	-0.399602	-2.316121
H	-4.486547	-1.201589	-3.061003
H	-4.639608	0.575548	-2.845207
H	-5.704365	-0.525544	-1.932299
C	-3.927453	0.650548	-0.124419
H	-4.863029	0.411015	0.423225
H	-4.033569	1.654677	-0.581456
H	-3.111097	0.709092	0.631320
C	1.372024	-2.719212	-1.468516
H	1.118782	-3.731346	-1.094987
H	1.981611	-2.209315	-0.697676
H	2.004445	-2.842475	-2.371783
C	0.135322	-0.156517	-2.793008
H	0.840483	0.500886	-2.248339
H	-0.810351	0.401475	-2.939883
H	0.559122	-0.379856	-3.793814
C	-1.339289	-2.841107	-2.906280
H	-0.822159	-3.117478	-3.850000
H	-2.279986	-2.316926	-3.170433
H	-1.614628	-3.774887	-2.375328
B	0.845063	1.006770	0.207659

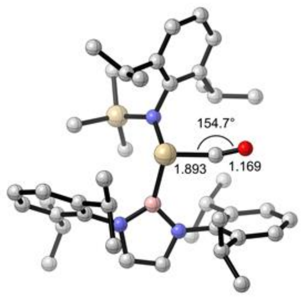

BP86-D3(BJ)/def2-svp

Thermal correction to Gibbs Energy = 0.837405
Thermal correction to Enthalpy = 0.986279
Sum of electronic and thermal Free Energies = −2479.161211
Sum of electronic and thermal Enthalpies = −2479.012338

Atomic Type	Coordinates (Angstroms) X	Y	Z
Si	0.437681	-0.107724	-0.788753
Si	0.805891	-2.198634	1.597066
N	-2.535806	0.353141	0.231957
N	-1.255875	2.247385	0.260828
N	1.415301	-1.240973	0.212706
C	-3.352416	1.461660	0.504285
H	-4.429930	1.349855	0.662287
C	-2.588971	2.596477	0.511903
H	-2.889141	3.637883	0.673796
C	-3.115768	-0.944369	0.063065
C	-3.659179	-1.618785	1.188556
C	-4.237164	-2.888220	0.986256
H	-4.660433	-3.431062	1.845460
C	-4.256676	-3.479746	-0.282062
H	-4.694238	-4.481337	-0.416865
C	-3.721893	-2.795098	-1.383457
H	-3.755514	-3.262883	-2.378038
C	-3.162601	-1.510431	-1.242724
C	-2.699683	-0.716050	-2.460978
H	-1.800200	-0.140152	-2.157866
C	-3.777650	0.307361	-2.874455
H	-4.713451	-0.206359	-3.181708
H	-3.423869	0.919382	-3.730460
H	-4.022542	0.997427	-2.042242
C	-2.272127	-1.589805	-3.646062
H	-1.514006	-2.338816	-3.340905
H	-1.819225	-0.958131	-4.437145
H	-3.128290	-2.128181	-4.105640
C	-3.631865	-1.010169	2.587704
H	-2.928633	-0.151646	2.560555
C	-5.022415	-0.472418	2.985496
H	-5.765074	-1.297251	3.027737
H	-5.400609	0.276965	2.262226
H	-4.988050	0.006673	3.986397
C	-3.121275	-2.000057	3.650783
H	-3.836134	-2.833843	3.813298
H	-2.989431	-1.485749	4.625186
H	-2.148753	-2.439270	3.359542
C	-0.256053	3.247165	0.050277
C	0.691375	3.508231	1.078982
C	1.674710	4.481601	0.828571
H	2.432972	4.698798	1.594338
C	1.710806	5.176367	-0.391303

H	2.494213	5.929876	-0.568269
C	0.762640	4.908638	-1.386239
H	0.810169	5.451721	-2.342617
C	-0.240982	3.939368	-1.187787
C	-1.224388	3.593214	-2.303630
H	-2.070065	3.038838	-1.848234
C	-1.812997	4.844542	-2.978869
H	-2.600016	4.555561	-3.705893
H	-1.043701	5.415364	-3.540520
H	-2.266806	5.531132	-2.234944
C	-0.572417	2.653196	-3.338787
H	-0.259604	1.691070	-2.880094
H	0.328539	3.116394	-3.793240
H	-1.286775	2.415885	-4.154953
C	0.638158	2.719842	2.382905
H	0.461773	1.661972	2.090709
C	1.944202	2.767465	3.184797
H	1.890783	2.063192	4.040091
H	2.135133	3.777738	3.605331
H	2.817291	2.484982	2.565472
C	-0.546059	3.155819	3.271662
H	-1.517941	3.043397	2.753878
H	-0.436236	4.218590	3.575258
H	-0.581859	2.538355	4.193591
C	2.698184	-1.619570	-0.328618
C	3.877221	-0.985376	0.164443
C	5.131262	-1.432190	-0.298178
H	6.047831	-0.961499	0.090831
C	5.234054	-2.455120	-1.249822
H	6.223617	-2.792388	-1.596476
C	4.068979	-3.032103	-1.771318
H	4.149498	-3.818683	-2.539081
C	2.793070	-2.629176	-1.330487
C	1.555199	-3.267529	-1.951246
H	0.672563	-2.850839	-1.424666
C	1.530993	-4.795074	-1.762483
H	1.628421	-5.072755	-0.692751
H	2.361186	-5.289404	-2.310215
H	0.579293	-5.222779	-2.142159
C	1.426723	-2.876289	-3.435818
H	2.303295	-3.221257	-4.024202
H	1.351463	-1.774594	-3.542029
H	0.518673	-3.329433	-3.886739
C	3.814184	0.167359	1.166313
H	2.760036	0.522517	1.175939
C	4.162678	-0.278407	2.600612
H	3.443632	-1.024135	2.988302
H	4.155652	0.588140	3.294017
H	5.175176	-0.733694	2.637193
C	4.722702	1.340215	0.750023
H	5.796978	1.079512	0.853355

H	4.540425	2.221869	1.399633
H	4.545314	1.648850	-0.297685
C	-0.634886	-3.284838	1.038023
H	-0.258560	-4.085181	0.369440
H	-1.403661	-2.715140	0.482873
H	-1.138211	-3.775625	1.896820
C	0.289860	-1.007728	2.970282
H	-0.546221	-0.361137	2.631707
H	1.138831	-0.349609	3.245340
H	-0.039718	-1.548084	3.881319
C	2.165800	-3.384819	2.157052
H	1.784077	-4.001289	2.998858
H	3.097812	-2.886924	2.483772
H	2.432791	-4.069466	1.326102
B	-1.158970	0.808864	0.071107
C	1.630478	1.354329	-0.940333
O	2.450507	1.998447	-1.468671
